# Gallium Nitride‐Based Electrode Materials for Supercapacitors: From Wide Band Semiconductor to Energy Storage Platform

**DOI:** 10.1002/smll.72944

**Published:** 2026-02-26

**Authors:** Farasat Haider, Batool Esmat, Ali Raza Kashif, Muhammad Shahid Khan, Akif Safeen, Basit Ali Khan, Karim Khan, Basit Ali

**Affiliations:** ^1^ Graduate School of Nanoscience and Technology Chulalongkorn University Bangkok Thailand; ^2^ Institute of Chemistry The Islamia University of Bahawalpur Bahawalpur Punjab Pakistan; ^3^ School of Materials Science and Engineering Huazhong University of Science & Technology Wuhan P. R. China; ^4^ IRC Sustainable Energy Systems King Fahd University of Petroleum and Minerals Dhahran Saudi Arabia; ^5^ Department of Physics University of Poonch Rawalakot Pakistan; ^6^ School of Materials Science and Engineering Shanghai University Shanghai P. R. China; ^7^ Centre For Clean Energy Technology School of Mathematical and Physical Sciences Faculty of Science City Campus University of Technology Sydney Broadway New South Wales Australia; ^8^ Department of Chemistry and Materials Science School of Chemical Engineering Aalto University Aalto Finland

**Keywords:** composite electrodes, energy storage, gallium nitride, nitride semiconductors, supercapacitors

## Abstract

Gallium Nitride (GaN) is transforming power electronics and optoelectronics; not only that, but it is also quickly becoming a primary component of the new generation of supercapacitors. GaN is a unique material that combines excellent electrochemical stability and tunable nanostructures with the best electrical properties. This review analyzes recent developments in GaN‐based supercapacitor technology, emphasizing the rationale behind the increased research in this area and presenting the main challenges. The enhancement of the field of pure and porous GaN to more elaborate hybrids, including GaN with carbon materials, or transition metal oxides or nitrides, or doping with some metal. The main parameters that have been summarized here include specific capacitance up to 1915.5 mF cm^−2^, energy and power density up to 13.3 mWh cm^−2^ and 1000 mW cm^−3^, and cycle stability remains high at 99 % despite 10 000–50 000 cycles. Chemical view brings together the connections between the different methods of synthesis, which include Chemical Vapor Deposition (CVD), hydrothermal processes, and electrochemical etching, and how these have been applied to affect electrochemical performance. When comparisons are made between electrodes, electrolytes, and device designs, then a better understanding of how GaN accumulates charge and which factors deplete it is achieved.

## Graphical abstract

This review focuses on capability of Gallium Nitride (GaN)‐based supercapacitors, bordering the advancement from porous architecture to novel hybrid nanostructures. It systematically investigates synthesis approaches and charge storage mechanisms that generate remarkable energy densities and competent cyclic stability. The discussion extends to overcoming scalability challenges, promoting GaN as key technology for future wearable and integrated power electronics.

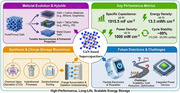

**Abbreviations**
ALD: Atomic Layer deposition
ASC: Asymmetric Supercapacitor
CC: Carbon Cloth
CNTs: Carbon Nanotubes
CV: Cyclic Voltammetry
CVD: Chemical Vapor Deposition
DFT: Density functional theory
DOS: Density of States
EDLC: Electric Double Layer Capacitance
EIS: Electrochemical Impedance Spectroscopy
ESG: Electrochemical solution growth
ESR: Equivalent series resistance
FCC: Face Centered Cubic
GaN: Gallium Nitride
GaNMM: Gallium Nitride Mesoporous Membrane
GCD: Galvanostatic Charge‐Discharge
HCP: Hexagonal Close Packing
HEVs: Hybrid Electric Vehicles
HSC: Hybrid Supercapacitor
HVPE: Hybrid Vapor Phase Epitaxy
ILs: Ionic Liquids
IoT: Internet of Things
LIBs: Lithium‐Ion Batteries
MBE: Molecular beam epitaxy
MEMS: Microelectromechanical Systems
MOCVD: Metal Organic Chemical Vapor Deposition
MOVPE: Metal Organic Vapor Phase Epitaxy
MSCs: Micro Supercapacitors
MWCNT: Multi‐walled Carbon Nanotubes
PANI: Polyaniline
PECVD: Plasma‐enhanced chemical vapor deposition
PPy: Polypyrrole
PVA: Polyvinyl Alcohol
PVA: Polyvinyl alcohol
rGO: Reduced Graphene Oxide
SCs: Supercapacitors
SiC: Silicon Carbide
TEM: Transmission electron microscopy
TMGa: Trimethylgallium
TMNs: Transition Metal Nitrides
VN: Vanadium Nitride
WBGS: Wide bandgap semiconductors

## 1 Introduction

Electric vehicles and electronic devices are on the rise, and the market of large‐scale energy storage is growing. This has led to a rush that has forced the industry to find materials that give them great power, thermal stability, and good carrier mobility [1]. The supercapacitors form the latest options as well as bridge the gap between the batteries and traditional capacitors, which improves properties such as fast charging and discharging, remarkable power density, and a long cycle life [2, 3]. The increased necessity of supercapacitors is because energy systems should be more efficient. Particularly, gallium nitride (GaN) is considered because its material properties allow many advanced technologies [4, 5]. GaN is suitable for high‐performance photodetectors, including photodetectors operating in environmental monitoring and security systems, because of its high bandgap and its exceptional sensitivity to UV light [6].

In the energy storage technology, a lot of advantages of GaN can be shown, such as: high breakdown field, high density of charge carriers, superior thermal conductivity, and high chemical stability, which make it the most promising material in the achievement of efficient and durable functionality of gadgets [7, 8]. GaN is proving to be a promising material as an energy storage candidate with significant properties, including the large bandgap, allowing good power operation, thermal stability, and high electron mobility, which in turn provides specific benefits to high temperature operation [9]. These characteristics can be attributed to the high density of the solid state of GaN, which improves the transport of charges because its storage mechanisms are better. In fact, large values of electron mobilities and saturation velocities allow fast cycles of charge and discharge, which would be problematic in advanced storage systems [10, 11], and the large bandgap also allows reliable operation at rising applied voltages and temperatures.

The natural limitation of the use of GaN as a supercapacitor in application is the necessarily small specific surface of GaN [12], which limited electrochemical capacitance. To overcome this, a lot of research is being conducted on the preparation of the porous GaN nanostructures and GaN‐based composite materials, which would result in a colossal increment of the available surface area and hence enhancement in general electrochemical properties. The reduced graphene oxide (rGO) is highly proclaimed to have a conductance of charge, high mechanical strength, high surface area, and chemical stability, thus can be used in supercapacitors, batteries, fuel cells, and bio‐source applications [13, 14, 15, 16]. Scientists have prepared nanocomposites of transition metal oxides and conducting polymers to empower the supercapacitive quality of rGO [17, 18]. Gallium nitride GaN has a very high potential as an additive to enhance the capacitive characteristics of rGO due to its large bandgap, high melting point, and increased thermal stability [19]. Graphitic carbon nitride (g‐C_3_N_4_) is also important due to its high specific surface area, two‐dimensional structure, and low cost, among other reasons, making it a suitable alternative to the established carbon‐based materials [20].

Transition Metal nitrides (TMNs) comprise gallium nitride (GaN), which has excellent electrical conductivity, exclusive electronic structure, and is remarkably good in electrochemical reactions, suggesting that they can augment energy storage processes [21]. The individual capacity, however, is commonly constrained by their metallic‐nitrogen bonds (M‐N), restricting the supply of d‐electrons to undergo electrochemical reactions with adsorbates. To address these issues, alternate methods are being implemented, like designing specific high surface area materials or the incorporation of metal ions that are highly oxidized to enhance pseudocapacitive contribution. Hierarchically structured porous materials have some benefits in this respect, including a high specific surface area, hierarchical pore structures that provide good penetration of electrolytes and can sustain expansion of the volume, and a synthesis of the rapid kinetics of nanomaterials with the packing density of micro‐scale materials.

Another trend for the latest development of energy storage based on GaN is to enhance the characteristics of a specific device through the enhancement of the performance of new heterostructures with the synergetic effect existing between the elements. Transition metals oxides like nickel cobalt oxide (NiCoO_2_) [22] have also been increasingly considered in energy storage, when used in combination with other materials, including graphite, MXenes, Carbon Nanotubes (CNTs) [23], transition metal oxides, carbon nanofibers, or Metal Nitrides [24, 25]. Doping NiCoO_2,_ especially with GaN, is of interest due to the increased electrochemical properties. In that regard, the porous structure of GaN is used as a scaffold, highly conductive, and NiCoO_2_ is the source of the various active sites, which will bring more capacitance and efficiency to the overall device. This means that the next generation of supercapacitors must be oriented towards the future aim of enhancing specific energy without compromising the parameters presented by specific power or cycle lifetime. Subsequently, the advancements of the next‐generation supercapacitors are guided towards the specific energy enhancement, devoid of degradation of either specific power or cycle life. According to this perspective, electrode materials used must have a high electric conductivity, high ionic conductivity, high surface area available to the ions, and high electrochemical stability.

Next, there is the production of GaN‐based heterostructures of high temperature supercapacitors (SCs), which has garnered a lot of interest owing to its applicability in high temperature conditions [26]. The performance traits of supercapacitors (SCs) include high‐power density, rapid energy harvesting, and exceptionally long cycle life, which provide them with behaviors that are optimum in extreme environments [27, 28]. In the hybrid electric vehicle, the engine temperature may rise to more than 140°C, and subsurface exploration, certain medical rescue, and military operations demand that it be able to work in temperatures above 250°C. The traditional organic battery substances cannot withstand this condition because the boiling points of the electrolyte materials used are low [29]. So, the necessity of cheaper, uniform high‐performance Supercapacitors which work in temperatures that are higher than extremes.

This review provides a profound and comprehensive analysis of the material based on gallium nitride (GaN) in relation to its use in supercapacitors and the specific discussion of its crystal structure, its natural features, its electrochemical performance, and prognosis. This review assesses the incentive behind the significant developments in the topic and the need for composite materials in enhancing the operation of the GaN‐based supercapacitors. It will also totally compare the different composite materials depending on GaN, and the research will be conducted on how different combinations of materials can be utilized as a three‐electrode or a two‐electrode combination with GaN. The problems that arose in the actualization of the GaN‐based supercapacitor during the practical implementation will be listed and discussed in the review.

## 2 Charge Storage Mechanism and Electrolytes in Supercapacitors

### 2.1 Charge Storage Mechanism

**FIGURE 1:**
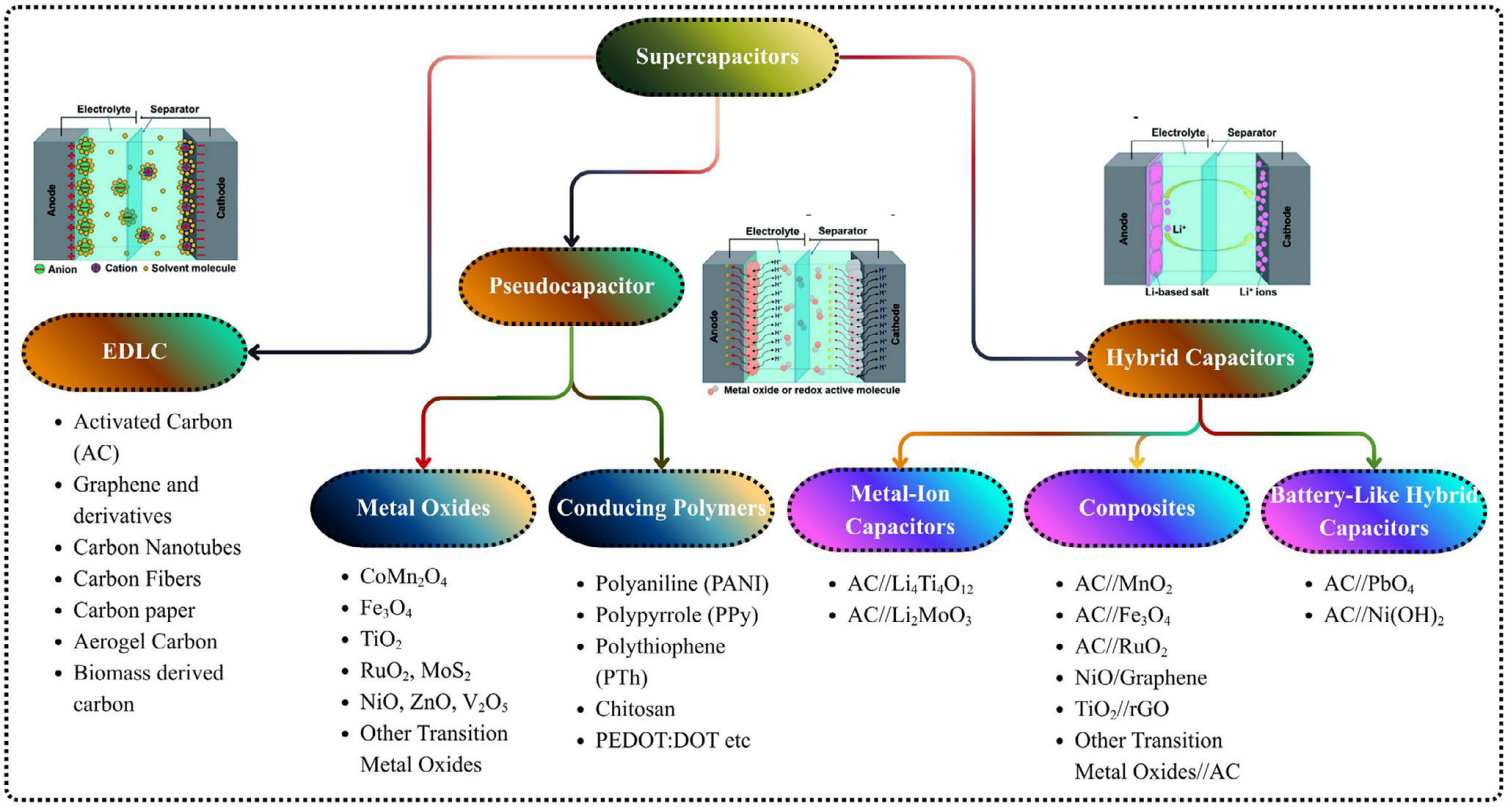
Types of supercapacitors in terms of electrode materials and the charge storage mechanism.

Building supercapacitors is easier than other components, especially when it comes to keeping them small. Supercapacitors require separators to prevent short circuits, electrolyte leakage, and self‐discharge. Due to such incidents, Supercapacitors must be sealed. Supercapacitors are classified into three categories, as shown in Figure 1, according to the type of electrode materials and their charge storage mechanisms. The charge storage mechanisms of semiconductor‐based electrodes are influenced differently depending on their structure, composition, and electrolyte ion interaction. Supercapacitors operate with three mechanisms: electric double‐layer capacitance (EDLC), pseudocapacitance, and the hybrid or asymmetric (ASC) capacitance, which is governed by different physicochemical processes.

#### 2.1.1 Electric Double‐Layer Capacitors (EDLCs)

Electric Double‐Layer Capacitors (EDLCs) store energy by accumulating charges statically at the interface of the electrolytes and electrodes [30, 31]. Performing this capacitance does not involve any chemical alterations or redox reactions. The assembly of the electric double‐layer, which stores charge, occurs when a voltage is applied. Electrolytes have opposite charges, free and bind around the electrode, and attach themselves to the electrode surface. This interface does not involve the crossing of electrons and direct Faradaic processes; therefore, they can be charged and discharged rapidly [32, 33]. This cycle can be performed infinitely. When it comes to EDLC electrodes, carbon‐based materials like graphene, carbon nanotubes, and activated carbon, EDLC electrodes are the principal materials, since they are chemically stable, conductive, and have high surface area materials [34, 35, 36, 37]. Nevertheless, due to the absence of faradaic reactions, their energy density is substantially limited and would only reach a few Wh kg^−1^ [38].

#### 2.1.2 Pseudocapacitors

Conversely, Pseudocapacitors use energy as fast and reversible faradaic redox reactions that take place at or close to the electrode surface [39]. These responses constitute charge transfer between the electrode and the electrolyte species, which offer a significantly higher capacitance than EDLCs. Transition‐metal oxides (e.g., MnO_2_, RuO_2_, NiCo_2_O_4_) [40, 41, 42, 43, 44], metal nitrides (e.g., GaN, TiN, VN) [45, 46, 47], and conducting polymers like Polyaniline (PANI) and Polypyrrole (PPy) [48, 49, 50, 51]. The pseudocapacitance mechanism is intermediate between that of EDLCs and batteries; while it relies on redox reactions, it does not involve deep intercalation or bulk phase transformations. Instead, charge storage takes place through surface or near‐surface processes, maintaining fast kinetics and long cycle life. This mechanism allows Pseudocapacitors to deliver both high power and relatively high energy density.

#### 2.1.3 Hybrid or Asymmetric Supercapacitors (HSCs)

Hybrid or asymmetric supercapacitors (HSCs) exploit the advantages of EDLC and pseudocapacitive systems by combining two electrodes of different charge storage mechanisms, typically an EDLC‐type carbon electrode and a faradaic‐type semiconductor electrode [52, 53]. The energy delivered from this type of HSC is theoretically equivalent to that available from batteries. The EDLC electrode offers high capability as well as fast response time; however, it is usually associated with low energy density. The pseudocapacitive or battery‐type electrode stores energy based on redox reactions, thereby contributing extra energy to the cell. When combined, they significantly expand the working voltage window and increase total energy output from the device. A synergetic combination like NiCo_2_O_4_//graphene [54, 55], MXenes//PANI [56], NiZrSe_3_//rGO [57], NbSe_2_//PPy [58], and MoS_2_//activated carbon [59] define practically attainable hybrid supercapacitor systems giving energy densities in the 40–60 Wh kg^−1^ range at a power density greater than 10 kW kg^−1^ [60]. Hybrid systems are thus one of the practical paths around the roadblock of an energy‐power trade‐off in single‐mechanism devices. All three types of supercapacitors are listed in Figure 1, while EDLC, Pseudocapacitors, and battery‐type are listed in Table 1 based on their Electrochemical parameters.

**TABLE 1:** Comparison of EDLC, Pseudocapacitors, and battery‐like electrodes based on different electrochemical parameters [61, 62, 63, 64].

Electrochemical parameters	EDLC	Pseudocapacitors	Battery‐like electrodes
Charge storage mechanism	Electrostatic charge separation occurs mostly at the electrode‐electrolyte interface	Faradaic reversible redox reactions and electron transfer between the electrode and electrolyte	Diffusion‐controlled reactions and transfer of ions between the positive and negative electrodes.
Materials	Carbon‐based materials like Activated Carbons, carbon nanotubes, MWCNT, MXenes, Graphene, and its derivatives	Transition Metal Oxides, Transition Metal Sulfides, metal hydroxides, and Conducting Polymers	Lithium‐Ion, Potassium‐ion, Sodium‐ion, and Aluminum‐Ion
GCD curves	Perfectly triangular and symmetric	Quasi‐triangular with slight curvature	Contains distinct potential plateaus
CV curves	Nearly ideal rectangular	Quasi‐rectangular or weakly redox‐shaped curve	Contains distinct redox peaks or plateau‐like features
Energy density	Lower than batteries (typically, 1–10 Wh kg^−1^)	Higher than supercapacitors but lower than batteries (10–50 Wh kg^−1^)	Maximum (100‐250 Wh kg^−1^ for LIBs).
Power density	Higher than batteries	High, but lower than EDLCs	Lower than supercapacitors
Specific capacitance	Typically, it ranges from 100 to 300 F g^−1^	Higher than EDLC Typically, it ranges from 200 to 1000 F g^−1^	No concept of capacitance due to the absence of charge.
Charge/Discharge	Very high	Very high	Slower compared to supercapacitors
Cycle life	Very high	Very high	Slower compared to supercapacitors

### 2.2 Electrolytes and Their Role in Charge Storage

Electrolytes are central to supercapacitor function, dictating voltage range, ionic conductivity, safety, and overall electrochemical performance [65]. They serve as conduction channels of ions, allowing efficient charge movement in the course of operating the device. Electrolyte systems may be classified as either aqueous, organic, ionic liquid, or solid‐state. Aqueous‐based electrolytes like KOH [66], Na_2_SO_4_ [67, 68], K_2_SO_4_ [69], and H_2_SO_4_ [70], are commonly used due to their high ionic conductivity and low internal resistance, which allows them to support fast charge/discharge cycles and high power density. However, the maximum energy density is limited by their ability to be stably electrochemically only to the range of about 1 volt. To overcome this, aqueous alternatives such as Na_2_SO_4_ [71] and Li_2_SO_4_ [72] have been investigated, aiming to extend voltage windows and minimize issues like electrode corrosion or gas evolution.

Organic electrolytes, such as those of solvents such as acetonitrile (AN) or propylene carbonate (PC), with salts such as TEABF_4_ [73], allow much broader voltage windows to 2.7–3.0 V, leading to much higher energy densities. Nevertheless, these have the disadvantage of low ionic conductivity, combustibility, and toxicity, bringing about safety and cost issues. The most remarkable feature of ionic liquid electrolytes, such as EMIMBF_4_ [74] and BMIMPF_6_ [75], is the wide range of their electrochemical stability (they can generally be over 4 V), non‐volatility (notable), and thermal stability. They can operate at high voltages and have a long lifespan in the cycle, but have a high viscosity and cost, thus restricting ion‐mobility and practice.

**FIGURE 2:**
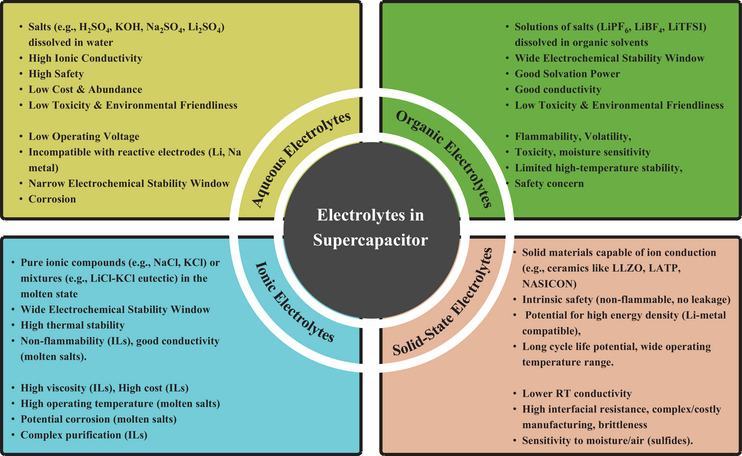
Classification of the different types of electrolytes in SCs and their characteristics.

Recent developments in supercapacitors have shown that their electrochemical performance may be greatly improved by substituting solid‐state electrolytes for liquid electrolytes [22, 76]. Flexible and wearable energy storage is a promising potential degree of solid‐state and gel polymer electrolyte. A typical example is polyvinyl alcohol (PVA) as a polymer with several salts, like H_3_PO_4_ [77], NASICON [78], and LiCl [79] which form quasi‐solid electrolytes that have mechanical flexibility but have a fair ionic conductivity [80]. Such systems provide device safety (by removing leakage hazards) and are compatible with small or novel device systems. The overall performance of the supercapacitors is, finally, determined by the close attention to the choice of the combinations of electrolytes and electrodes [81]. This dictates stability, compatibility with the electrode‐electrolyte interface, and overall energy output. In the case of electrodes that are highly reactive to the surface, e.g., semiconductors, it is important to use electrolytes that do not lead to a degradation of the material and that nevertheless ensure efficient ionic flow (Figure 2).

### 2.3 Need for Supercapacitors in Energy Storage

**FIGURE 3:**
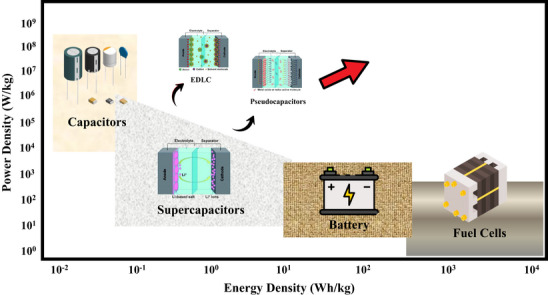
Ragone plot for energy storage devices.

The growing international need to have an effective and sustainable energy storage mechanism is a pressing matter in the face of growing global energy consumption. The energy mix remains dominated by fossil fuels, as projections of the world's primary energy consumption up to 2050 still indicate that petroleum and natural gas continue to be key contributors, even though they see a rise in renewables [1, 82]. Though renewable energy sources like solar and wind have high potential in decarbonization, their intermittency, coupled with unpredictable generation, creates instability in the current grids. Therefore, efficient and fast‐response energy storage technologies would be necessary to enable the integration of renewables and uphold grid stability [83, 84]. Figure 3 (Ragone plot) shows how the supercapacitors are placed in the energy‐power density space in relation to each other. Supercapacitors are used in the middle between the high‐power, yet low‐energy dielectric capacitors and high‐energy, yet low‐power batteries. In particular, SCs have energy and power densities of 10–180 Wh kg^−1^ and 100–10 000 W kg^−1^, respectively, which makes them convenient in fast energy transfer and short‐performance scenarios.

Traditional electrochemical batteries, including lithium‐ion [85] and lead‐acid, have high energy densities but have a limited cycle life, low charge–discharge rates, and are not as safe as desired in high‐power, short‐duty applications [86]. These shortcomings have stimulated the study of supercapacitance (SC), also referred to as ultracapacitors or electrochemical capacitors, that can close the performance gap between traditional capacitors and batteries [87, 88]. Supercapacitors have a high‐power density of capacitors with a moderate energy density of batteries, which allows rapid energy access, high reversibility, and long operation life [89]. Table 2 summarizes the performance of supercapacitors, lead‐acid batteries, and lithium‐ion batteries in comparison with each other.

**TABLE 2:** Comparison between supercapacitors and li‐ion batteries [90, 91, 92].

Parameters	Supercapacitors	Li‐ion batteries
Efficiency (%)	> 98	∼ 85–95
Charge time (s)	1–60	3600–18 000
Device voltage (V)	2.3–2.8	12–24
Hot temperature (°C)	+70 to 85	+45
Cold temperature (°C)	−40	−20
Operating temperature (°C)	−40–85	−20–65
Energy density (Wh kg^−1^)	10–180	250
Power density (W kg^−1^)	∼10 000	50–200
Cyclic life	> 1 000 000	10 000
Cost per W h^−1^	$20	$0.50–1.00
Lifetime (Years)	5–20	3–10
Charge rate	> 1500	˂ 40
Discharge per month	60	4

Supercapacitors show more than 98 % efficiency compared to lead‐acid (∼70 %), and lithium‐ion batteries (85–95 %) [93]. They have a cycle life of more than one million charge–discharge cycles, which is a great modification compared to conventional battery technologies. Another important benefit is the power density, which reaches 10 000 W L^−1^ with SCs and 1000 W L^−1^ with conventional batteries. Nonetheless, the energy density of supercapacitors (10–180 Wh L^−1^) is still part of lithium‐ion (100‐250 Wh L^−1^) and lead‐acid batteries (30–50 Wh L^−1^), but hybrid systems are gradually closing the gap [94]. Moreover, SCs are stable over a wide temperature range (−40°C to +85°C), which gives them resilience in harsh conditions where traditional batteries fade too quickly [95].

Nevertheless, the high‐power density and life cycle of the supercapacitors have a fundamental limitation in energy density. The energy storage through electric double‐layer capacitors (EDLCs) is limited by the amount of electrically charged particles that can be held in a defined unit of mass or volume (electrostatic charge storage). The lack of bulk faradaic reaction and the narrow electrochemical stability window of electrolytes are responsible. As a result, enhancement of the electrode surface area, the development of redox‐active materials, and the formation of hybrid configurations are major strategies that are important to address this energy density gap so as to increase the overall energy storage capacity of supercapacitors.

**FIGURE 4:**
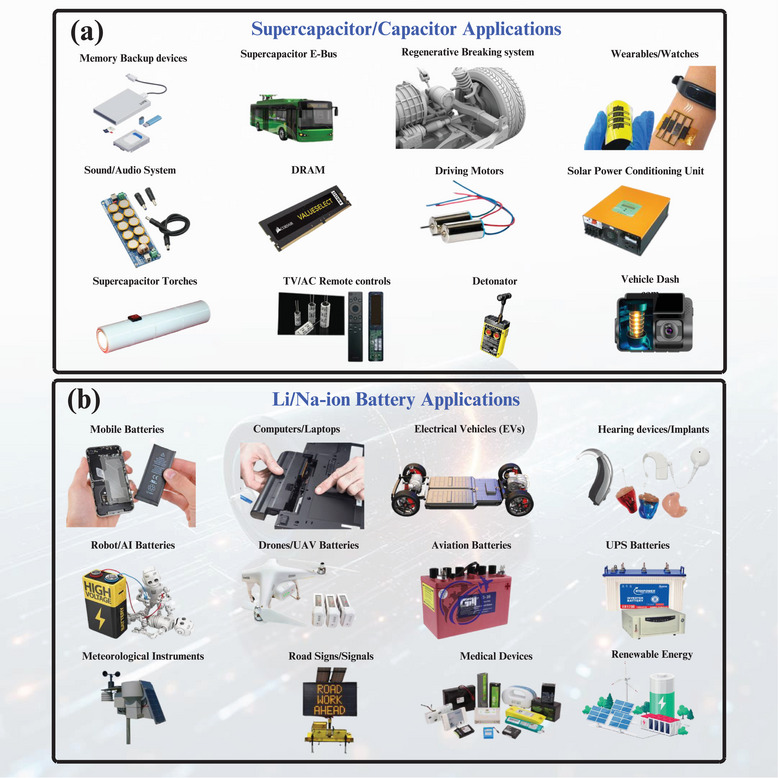
Applications for (a) supercapacitors, and (b) batteries across different sectors.

Supercapacitors are being used in sophisticated energy systems due to their high‐power production and good cycle life. They have become an essential part of electric vehicles (EVs) and hybrid electric vehicles (HEVs) in the transportation industry, where quick charge–discharge dynamics are useful in offering regenerative braking and aiding acceleration [96]. SCs improve both energy efficiency and battery life by harnessing and recycling kinetic energy that would go to waste as heat. They can be used to alleviate the intermittent generation of solar and wind systems in renewable energy networks as an energy buffer with the rapid‐response feature. They have the ability to absorb excess power fast in times of overproduction and release it in times of underproduction to even out voltage and frequency variations in microgrids. Likewise, the SCs can be used in portable electronics and Internet of Things (IoT) systems to provide immediate bursts of power to data transmission, sensors, and wireless modules, providing higher autonomy and reliability in terms of energy [97]. Applications for supercapacitors and batteries in different sectors are illustrated in Figure 4a,b. Hybrid approaches, which combine lithium‐ion batteries and SCs, merge the benefits of both systems: SCs are used in high‐power situations and batteries in long‐duration power supplies. This constructive interaction focuses on the maximum performance of the system, its lifespan, and solves the power and energy needs.

The newer trends of supercapacitor technology revolve around the innovation of materials. The studies are aimed at nanostructured electrodes (including graphene, MXenes, metal oxides, and hybrid carbon‐metal composites) to improve the capacitance and energy density [98]. Faradaic and non‐faradaic hybrid Pseudocapacitors are under development to realize higher specific energy, and cycling stability is preserved [99]. Another developing solution to create hybrid renewable energy ecosystems that can balance power and energy needs is integration with fuel cells and photovoltaic systems. The industry still focuses on scalable fabrication, cost‐cutting, and flexible architecture of devices in smart grids, autonomous systems, and wearable electronics. They are very important in the evolving world of energy storage technologies, which is one means of bridging the gap between short‐term power delivery and long‐term storage of energy. Their real‐time charge–discharge capacity and stability render them indispensable in transportation, renewable integration, and next‐generation electronic systems. Further developments in materials and hybrid structures will guarantee that the supercapacitors will continue to play a key part in the global shift toward sustainable, efficient, and decarbonized energy systems [100].

## 3 Crystal Structures of GaN

### 3.1 Cubic (Zinc Blende) and Hexagonal (Wurtzite) Gallium Nitride

**FIGURE 5:**
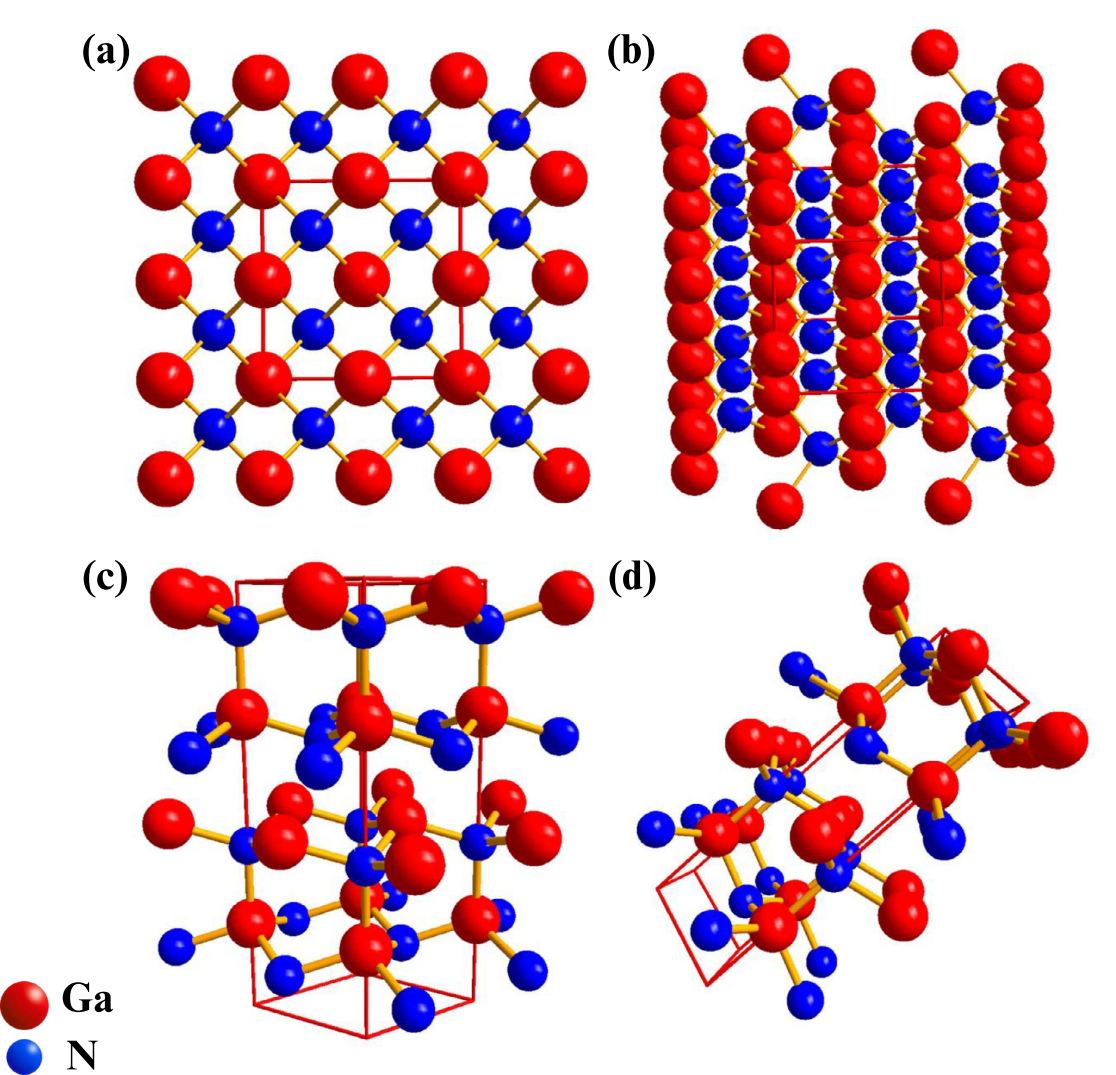
(a,b) Cubic Zinc Blende, and (c,d) Hexagonal Wurtzite crystal structures of gallium nitride showing the atomic arrangement of gallium (Red) and nitrogen (Blue) atoms (Data generated from Copyright Materials Project and drawn in Diamond 5).

Gallium nitride (GaN) is a material with a hexagonal wurtzite (w‐GaN) and cubic zinc blende (z‐GaN) structure [101], as illustrated in Figure 5. The zinc blende structure also follows a face‐centered cubic (FCC) lattice structure, where every gas atom is tetrahedrally coordinated with four nitrogen atoms (and the same way round). This configuration is highly symmetrical, and this is typical of a mineral, zinc blende (ZnS). This structure consists of atoms that are twelve nearest neighbors. This is because the cubic crystal exhibits high symmetry that makes its physical properties isotropic, meaning it does not depend on which direction one is in in all the crystallographic directions. The wurtzite hexagonal structure comprises alternating layers of Ga and N atoms along the c‐axis. The tetrahedrally coordinated atoms are all Ga atoms, which are tetrahedrally coordinated to N atoms, and vice versa. The hexagonal phase is more asymmetric than the cubic phase and has hexagonal close packing (HCP), which causes the material to have anisotropic properties, as the properties of the material depend on the crystallographic orientation. The numerous structural characteristics of GaN, and especially the ability to exist in an isotropic and anisotropic form, are what have made it a material of choice in high‐temperature and high‐power electronics and storage processes [102]. GaN is also better than the traditional semiconductors such as silicon (Si), gallium phosphide (GaP), silicon carbide (SiC), and gallium arsenide (GaAs) in that it is thermally more stable and has higher electron mobility, breakdown voltages, and will be the material of choice in the next generation of electronic and optoelectronic devices.

### 3.2 Defects and Impurities

Such defects and impurities are significant in the determination of properties of the GaN material, particularly when such a compound is applied in storing energy of energy in the form of supercapacitors. The common imperfections in GaN are vacancies, interstitials, and dislocations [103] and the common impurities are silicon (Si), carbon (C), and oxygen (O) [104]. The defects of the structure would be incredibly beneficial in the improvement of the electric feature and optical attribute of GaN and can cause the failure of electronic and opto‐electronic equipment. One significant impurity that has been identified to be added to GaN in the growth processes includes carbon, which is added when using the metal‐organic chemical vapor deposition (MOCVD) and metal‐organic vapor phase elimination deposition (MOVPE) methods. Carbon introduction is also important because it forms carbon on nitrogen (C‐N) or carbon on gallium (C‐Ga) defects [105], which may lead to complex forms of compensation and defect clustering of the GaN lattice. It has been proven that these carbon‐related defects form parasitic leakage paths in the epitaxial layers and cause phenomena such as current collapse, increased power consumption, and low efficiency of the devices. The number of intrinsic defects will be introduced into silicon impurities in GaN, and their effects on the electric properties are extremely undesirable. Substitution of a lattice site of gallium by silicon makes silicon a deep acceptor, which traps the charge and will have an unfavorable impact on the inner quantum efficiency of optoelectronic devices, including LEDs. In cases where these defects are latent in the usage of a supercapacitor, literal translation of these defects is the elevated equivalent series resistance (ESR or R_s_) and diminished specific capacitance (C_s_), which will end up damaging the performance, efficiency, and the lifecycle of the item in terms of energy mis‐deposit. These defect complexes in supercapacitor applications block adsorption sites of ions and form localized insulating areas, causing a decrease in electrochemically active surface area. This destruction causes a direct reduction in the specific capacitance, one of the performance limits in any energy storage system, because it reduces the ability to collect charge and restricts the availability of ions at the electrode‐electrolyte interface.

## 4 Epitaxial Growth of Gallium Nitride

### 4.1 Metal‐Organic Vapor Phase Epitaxy (MOVPE or MOCVD)

**FIGURE 6:**
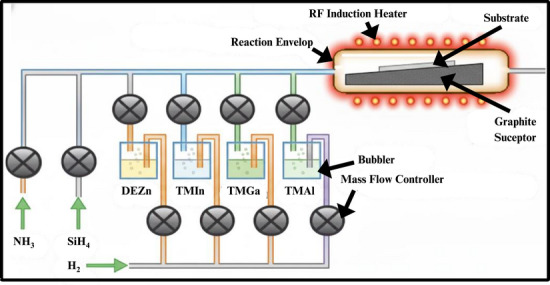
A representation of the MOCVD/MOVPE reactor.

The most recent technique of growing Gallium Nitride by epitaxial growth is that of Metal‐Organic Vapor Phase Epitaxy (MOCVD or MOVPE), which uses organometallic sources of gallium (ex, trimethylgallium (TMGa)) and nitrogen (ex, ammonia (NH_3_)). MOVPE, as a large‐scale way of commercializing the process, is suitable for considering the energetics of the growth of III‐Nitride semiconductors, as it provides substantial control of the key parameters of crystal quality, doping, and layer thickness, which are very important to state‐of‐the‐art electronic and optoelectronic devices [106]. The ammonia (NH_3_) reacts with trimethylgallium (TMGa) in a high‐temperature reactor to allow the growth of GaN. The metalorganic precursors have to be transported in the form of carrier gas, e.g., hydrogen (H_2_). The reaction is done at elevated temperatures of about 1000‐115°C and the process offers good crystalline growth through thermal breakdown of the raw materials and subsequent surface reaction. Figure 6 represents a schematic drawing of the MOCVD system. This heat processing environment is conducive to great mobility of the adatoms upon the surface, which leads to smooth morphologies and high‐quality crystals to use in device applications. The method requires a constant flow of gases, which are chemically reacted in a high‐temperature chamber to form the layer of GaN. MOVPE finds applications in optoelectronics, such as LEDs, and Energy storage, such as supercapacitors and battery electrodes.

### 4.2 Hydride Vapor Phase Epitaxy (HVPE)

**FIGURE 7:**
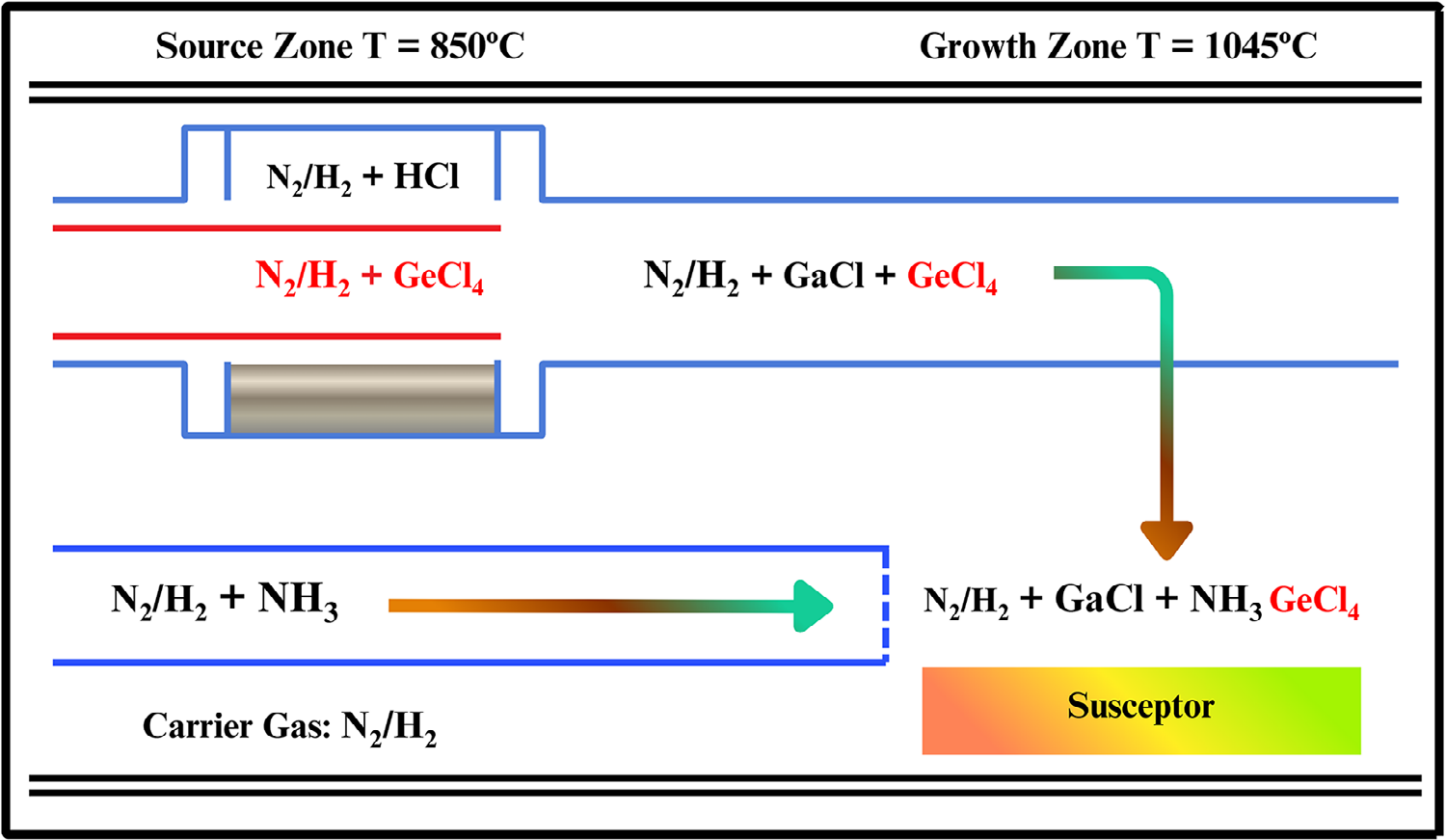
A representation of the HVPE reactor.

HVPE is the market leader in terms of generating large GaN films in the shortest time and bulk crystals. It is popular because the equipment is simple, the conditions do not require extreme measures to grow, and the rates are simply amazing [107]. The first step we can perform is the preparation of gallium chloride (GaCl) and the reaction of HCl gas with liquid gallium at 800–900°C. Subsequently, the substrate is reacted with GaCl and ammonia on the surface [108]. This is done in a quartz HVPE reactor, which consists of two distinct zones, one being the source, and another being the growth because they require different temperatures. Figure 7 shows the layout; the ammonia nozzle lies parallel with the susceptor, and GaCl and Germanium tetrachloride (GeCl_4_) descend on top via a spray nozzle. The substrate is located on a rotating susceptor disc, which is located on the right side of the reactor. You make GaCl, in which gallium reacts with hydrochloric acid at 850°C. The GaCl is introduced in the growth zone, where the temperature remains at 1045°C through carrier gas. It reacts at that point with ammonia to produce GaN. HVPE has enjoyed wide usage by researchers to produce bulk GaN crystals and freestanding GaN substrates, and this has been well recorded in the literature.

## 5 Characteristics of Gallium Nitride for Energy Storage

### 5.1 Electronic Properties of Gallium Nitride (GaN)

Gallium nitride (GaN) is a special material that is important for supercapacitor applications due to its electronic nature. It loads charge carriers into the unit of volume to a high degree, and the carrier mobility reaches as high as 495 cm^2^ V^−1^s^−1^. Charge flow on the electrode is high, which enhances electric conductivity and allows the device to charge and discharge at amazing speeds, exactly what supercapacitors are expected to do. The large bandgap of GaN is approximately 3.4 eV [109], so it is highly voltage tolerant with a high temperature stability, such that even as the temperature rises, supercapacitors remain operational. Hall effect and Mott‐Schottky measurements have proved that GaN is an n‐type semiconductor [110]. This n‐type helps aid reliable charge storage, both in pseudocapacitance and electric double‐layer capacitance (EDLC). Another benefit comes with porous GaN films: the structure of porous films generates numerous active sites, in which electrolyte ions can absorb, which enhances capacitance. Besides, GaN has defects and surface states that place an additional pseudocapacitive effect, pushing the energy storage of GaN even higher. To have a closer examination of the electronic properties, one can refer to Table 3 [111], for cubic and wurtzite GaN.

**TABLE 3:** Electronic properties of GaN [111].

Electronic properties	Wurtzite GaN	Zinc‐Blende GaN
Hole thermal velocity (m/s)	9.4 × 10^4^	9.5 × 10^4^
Holes	5	9
Hole mobility (cm^2^/V. s)	≤ 200	≤ 350
Electron mobility (cm^2^/V. s)	≥ 1000	≥ 1000
Electrons	25	25
Light	0.3	0.2
Breakdown field (V/cm)	∼5 × 10^6^	∼5 × 10^6^
Electron affinity (eV)	4.1	4.1
Heavy	1.4	1.3
Effective electron mass (m_o_)	0.20	0.13
Electron thermal velocity (m/s)	2.6 × 10^5^	3.2 × 10^5^

### 5.2 Thermal Properties of Gallium Nitride (GaN)

GaN possesses very good thermal properties, and this improves its application in energy storage systems. GaN also exhibits thermal conductivity ranging between 210 and 230 W m^−1^K^−1^, and this implies that it can cool quickly in various rapid charge and discharge cycles. This makes the components cool and reduces the chances of overheating, besides the life of supercapacitors in such a case as when the supercapacitors are under high‐power output. There is no added value in some form of thermal management in this case, but a stabilizing capability when the system is powered with high power, such as charging batteries quickly and large‐scale energy storage. Something that does not prevent thermal runways, nor has an efficient working system, will not be safe and effective in such conditions. To add to that, GaN supports excessive heat. Even at temperatures exceeding 600°C, it maintains its structure and electronic performance. This type of stability implies that the energy storage systems based on GaN can work well in harsh conditions, withstand wear and tear, and just need to live longer. To have a closer examination of the electronic and physical properties, refer to Tables 3 and 4, respectively, for cubic and hexagonal GaN.

**TABLE 4:** Physical properties of gallium nitride (GaN) [111].

Physical properties	Wurtzite GaN	Zinc‐Blende GaN
Density (g/cm^3^)	6.15	6.15
Thermal diffusivity (cm^2^/s)	0.43	0.43
Bandgap energy (eV)	3.39	3.2
Dielectric constant (Static)	8.9	9.7
Refractive index	2.33	2.33
Lattice constant (Å)	a = 3.189, c = 5.186	4.52
Density (g/cm^3^)	6.15	6.15
Coefficient (1/K)	Δc/c = 3.17 × 10^−6^ K	—
Bulk modulus (GPa)	210	210
Thermal expansion	Δa/a = 5.59 × 10^−6^ K	N/A
Melting point (°C)	2500	2500
Thermal conductivity (W/cm. K)	2.1	2.1
Heat capacity (j/mol)	35.3	35.3

### 5.3 Chemical Stability and Electrolyte Compatibility

GaN can be distinguished by its storage of energy since it simply does not respond much. It is not susceptible to the destructive electrolytes, organic solvents, and those repugnant ionic liquids that you will find in the high‐tech batteries. This resistance means that its electrodes do not ever discharge in any of those charge and discharge cycles, and this is why the batteries do not lose their capacity and have a longer life in cases of such applications as lithium‐ion and solid‐state. Combine its chemical and thermal durability, and GaN will begin to appear to be a genuine game changer, as far as energy storage is concerned, in the future. Using these strengths, engineers will be in a position to come up with small, efficient machines that can still be used in a rough environment. It is titanic to renewable energy and electrification when the only factor that matters is reliability. GaN electrodes can not only survive in an extreme environment in electrolytes but also be sustained in electrochemical activity [112]. Diffusion of the ions and charge storage are still effective. A porous GaN membrane is an example. They can hold about 98–99% of their capacitance following 10 000 cycles in ionic liquids as electrolytes. There is little degradation and fluid ion transfer.

### 5.4 Optical Properties and Photonic Integration

Gallium nitride, or GaN, is an element that is interesting due to its weird optoelectronic properties. This material does not merely work with light; in reality, when mixed with the technology that uses light with energy storage, new opportunities are opened. It has a direct bandgap of approximately 3.4 eV and a super‐high rate of UV absorption, making it a fast and efficient absorber of photons. They will therefore immediately be used in applications such as solar‐powered hydrogen generation or light charging batteries that will directly convert sunlight into stored energy using GaN electrodes. There is also the GaN that literally glows and has a strong photoluminescence that can be adjusted between UV and visible light [113]. That paves the way to energy devices that are not only energy storage devices. Imagine a solid‐state battery with in‐built GaN photodetectors; it can display a real‐time image to you of how much charge the battery is holding. Or consider the possibility of a supercapacitor, which in fact glows to indicate its remaining amount of juice. And to top it all, GaN can withstand heat and even withstand the effects of chemicals, and thus it is prepared to work hard. It can be easily integrated into the hybrid systems that combine harvesting solar energy and storing it in high‐density. Such multi‐talented designs are a game‐changer for small spaces, such as wearables or self‐powered IoT devices. In the case of GaN, you have combined the functionality of components, and devices are made simpler and more autonomous.

## 6 Gallium Nitride in the Context of Other Wide‐Bandgap Semiconductors

Traditional wide‐bandgap semiconductors, bearing bandgap energies above 2 eV, have received much interest recently as the foundation of future energy storage and related power electronics [114, 115]. The high electronic bandgap promotes high breakdown fields, high chemical/thermal durability, and high flexibility in their electronic properties. These properties are quickly becoming more mandatory in the function of high‐voltage‐operated supercapacitors. Although other semiconducting materials, such as titanium dioxide (TiO_2_) [116], zinc oxide (ZnO) [117], silicon carbide (SiC), aluminum nitride (AlN), and boron nitride (BN), have been scrutinized systematically, gallium nitride (GaN) holds a unique position in simultaneously encompassing high‐quality semiconducting properties, optoelectronics, and SIE properties. Table 5 lists the major parameters of popular wide‐bandgap semiconducting materials with significant applications in supercapacitors. Although the bandgap values of 3.0‐3.3 eV in both TiO_2_ [118, 119] and ZnO [120] materials have attracted much interest due to their surface redox properties, their inherently low conductivity makes them unsuitable. SiC and AlN, with high thermal conductivity and high‐temperature stability, exhibit admirable mechanical and dielectric durability, whereas their low surface activity and poor charge transfer properties restrict their applicability. Hexagonal Boron Nitride (h‐BN) with an extraordinarily wide bandgap energy of 5.5 eV [121]. Although chemically inert, it is mostly used in the role of the dielectric barrier or protective coating, rather than being utilized in high‐capacitance applications. GaN, with balanced properties between both ends, exhibits wide‐bandgap durability, semi‐conductivity, high optical interactions, and decent conductivity.

**TABLE 5:** Properties of common wide‐bandgap semiconductors relevant to supercapacitor applications.

Material	Bandgap (eV)	Key features	Role in SCs	Refs.
GaN	3.2–3.4	Direct bandgap, high electron mobility, tunable conductivity, photoactive	Hybrid and photo‐assisted electrodes	[122]
ZnO	3.3	Easy synthesis, oxygen vacancies, surface redox activity	Pseudocapacitive composites with rGO or polymers	[123]
TiO_2_	3.0–3.2	Chemically stable, abundant, n‐type	Pseudocapacitive; limited conductivity	[124]
SiC	3.0	High thermal and electrical robustness	Solid‐state and dielectric‐layer applications	[125]
AlN	6.0	Ultra‐wide bandgap, thermally conductive	Solid‐state devices, limited surface activity	[112]
BN (h‐BN)	5.5–6.2	Chemically inert, high dielectric constant	Dielectric or insulating layer	[126]

What makes GaN unique among other WBGS materials is the fact that it has a direct bandgap, the wurtzite structure, and the ability to be doped in n‐type, allowing it to take advantage of multiple functionalities in both the electrochemical and the optically aided charging of the storage device [127]. The spontaneous and piezoelectric polarization in the wurtzite structure of GaN also creates natural fields along surfaces and interfaces, squeezing the benefits of high ion adsorption and charge separation in both electrochemical and optically aided charging. Moreover, GaN has high electron mobility, with 1000–1500 cm^2^ V^−1^s^−1^, and high purity with lower defect diffusivity, ensuring suitable devices with stable cycles of charging and discharging. Doping with Si, oxygen, or transition metals can further enhance the conducting properties of GaN [128]. Table 6 presents the electrochemical performance of common wide bandgap semiconductors that are already discussed in Table 5, including their properties for Supercapacitors.

**TABLE 6:** Comparison of electrochemical performance of common wide‐bandgap semiconductors.

Material	Synthesis method	Specific capacitance	Cyclic stability	Energy density	Power density	Refs.
Porous GaN membrane	Calcination	52.58 mF cm^−2^	86.2 % after 10 000 cycles	13.3 mWh cm^−2^	67.5 mW cm^−2^	[129]
ZnO tetrapods	Oxidative‐metal‐vapor‐transport	160.4 F g^−1^	94.3 % after 1000 cycles	22.3 Wh kg^−1^	563.6 W kg^−1^	[130]
TiO_2_ nanotube arrays	Anodic oxidation	3.24 mF cm^−2^	92 % after 10 000 cycles	0.8 mWh cm^−2^	17.5 mW cm^−2^	[131]
SiC nanowires	Pyrolysis	23.6 mF cm^−2^	80 % after 10 000 cycles	—	—	[132]
AlN	Hydrothermal	240 mF cm^−2^	98.14 % after 10 000 cycles	3.88 µW h cm^−2^	4.25 mW cm^−2^	[112]
BN (h‐BN)	Ball Milling	615 F g^−1^	95 % after 5000 cycles	85.4 Wh kg^−1^	10.25 kW kg^−1^	[133]

The presence of optimized processes of nano‐structuring of GaN, including MOVPE, MBE, and HVPE, also contributes towards the fabrication of single‐crystal nanostructures, which have a regulated defect density and crystal orientation. The other important merit of the usage of GaN as compared to TiO_2_ or ZnO when it comes to practical application is the ability to be photoactive. That is, as long as they are illuminated, their ability to produce additional pairs of electrons and holes comes in to help them in the application of photo‐assisted supercapacitors [134]. Among the wide‐bandgap semiconductors group, it is worth mentioning gallium nitride (GaN), which is multifunctional, as the material can be used both in terms of the chemical resistance of ceramic oxides and regarding the ability to be constructed in terms of the electronic properties, alongside the optical properties of semiconductors. In fact, the strength of GaN lies in the fact that, beyond the fact that it is related to optoelectronics, the material signifies the beginning of a new era concerning energy storage.

## 7 Bandgap Engineering of GaN and Related Alloys (AlGaN, InGaN) for Energy Storage

Band‐gap engineering, or the controlled modification of the electronic properties of semiconducting materials by alloying, doping, or strain, is highly useful in the design of multiple functionalities in wide bandgap semiconducting materials [135, 136]. In particular, bandgap engineering is used in gallium nitride (GaN) semiconducting materials, where the simultaneous control of the electrical conductivity, carrier concentration, and optical properties of these materials is highly important in designing electrodes in supercapacitors [137, 138]. The intrinsic bandgap of GaN crystals, with either wurtzite or zinc‐blende structures, is broad (∼3.4 and ∼3.2 eV, respectively) and offers high thermal and chemical stabilities. However, their high bandgap strongly inhibits their conductivity and optical absorbability in the visible region.

Aluminizing Gallium Nitride with Aluminum, yielding Al*_x_* Ga_1_ *_‐x_* N, with a bandgap ranging from 3.4 eV in Gallium Nitride to 6.2 eV in Aluminum Nitride [139]. An increase in Aluminum enhances the bandgap, dielectric strength, chemical resistance, and breakdown voltage, which is desirable in high‐voltage devices and solid‐state supercapacitors. However, at high Al concentrations, the mobility is reduced by both lattice mismatch and scattering effects [140]. In any case, the AlGaN layers can become useful in dielectric barriers or protective layers, with both an improvement in interface integrity and electrolyte resistance. Gradients in AlGaN also enhance polarization at the interface layers, useful in ion adsorption and storage. On the other hand, with Indium, the ternary material created is In*_x_* Ga_1_ *_‐x_* N, decreasing the bandgap from 3.4 eV in GaN to 0.7 eV in InN with the addition of Indium, thus enhancing conductivity properties [141]. This renders InGaN helpful in the fabrication of visible‐light‐enabled photo‐assisted supercapacitors, wherein the generated charge carriers due to illumination help in storing charge. The addition of Indium increases local states, along with enhancing the concentration of charge carriers. Thus, electrodes with properties about various parts of the electromagnetic spectrum can be designed.

### 7.1 Electrochemical Insights of GaN Alloys

For electrochemical study, AlGaN nanoalloys with transition metal dopants as energy storage has been studied recently by Mollaamin [142]. He proposed a viable ternary semiconductor composed of aluminum gallium nitride, doped with silicon, germanium, palladium, or platinum, and conducted molecular modeling that considered the geometrical parameters of doping atoms on the surface of AlGaN. The study examined the absorption characteristics and the current charge density related to energy storage. He observed that energy storage using hetero clusters has specified that the frame of the overcoming cluster is connected to AlGaPdN or AlGaPtN in large levels of frequency. This feature renders AlGaPdN or AlGaPtN potentially favorable for some high‐frequency applications for energy storage due to hydrogen adsorption via production of H‐AlGaN, H‐AlGaPdN, H‐AlGaSiN, H‐AlGaPtN, or H‐AlGaGeN.

The benefits of platinum or palladium over aluminum gallium nitride include its better electron and hole mobility, allowing platinum or palladium doping devices to function at higher frequencies than silicon or germanium doping devices [143, 144]. In another paper, Mollaamin and Monajjemi [145] hypothesized that Manganese doping can change GaN materials into p‐type semiconductors, which is critical for creating p‐n junctions or p‐type layers in GaN based devices. They mentioned that Energy storage using heteroclusters has specified that the frame of the overcoming cluster is connected to MnGaN, MnAlGaN or MnInGaN in the large quantities of frequency. This feature makes these alloys potentially useful for certain high‐frequency applications needing batteries for energy storage owing to hydrogen adsorption via production of Mn(In, Al)GaN‐H. Additionally, Rodriguez et al. [146] explored the pseudocapacitive behavior of InN/InGaN QDs nanostructures as supercapacitor electrodes. They achieved an areal capacitance of 9 mF cm^−2^ for the InN/InGaN QD electrode in 1 M KCl aqueous electrolyte solution. These findings emphasize the controllability and variability of the bandgap in GaN‐based semiconductors, giving new potential uses for GaN as a material in optoelectronic devices and batteries.

### 7.2 Strain and Defect‐Induced Bandgap Modulation

Apart from compositional alloying, strain modulation and defect engineering provide dual approaches to modify the band structure of GaN. Controllably introduced nitrogen vacancies, gallium interstitials, or extrinsic dopants (Si, Mg, O) result in gap states that promote charge percolation and ion coupling at the electrode surface. Simultaneously, engineered junctions involving layers of GaN grown in a heteroepitaxial process, combined with conducting layers of material such as graphene, MXenes, or conducting polymers, promote band bending effects, thereby increasing the flow of both electron and ion current in galvanostatic systems [147, 148]. Therefore, this leads to the use of the wide‐bandgap supporting material GaN in a flexible photo‐sensitive electrochemical environment.

Bandgap engineered GaN and ternary alloys, therefore, offer a new design horizon toward optoelectronics and multifunctional supercapacitor integration [149]. The AlGaN layers act as high‐potential window substrate layers in solid‐state cells [150], whereas the optically active layers of InGaN and defect‐engineered GaN layers act as the charge storage layers [151]. Therefore, the integrative functionalities of optical flexibility, spontaneous polarization, and chemical robustness offer a new paradigm in which optoelectronic and electrochemical energy conversion devices could be monolithically integrated. Enhancement of epitaxial growth capabilities toward defect engineering, therefore, will offer a new horizon in the development of high‐performance, high‐energy density, visible‐light‐sensitive, high charge/discharge rate, and high‐power density GaN electrodes.

In an experiment, the researchers have demonstrated the results of an investigation on the Al_0.1_Ga_0.9_N/GaN solar cells with a Mn‐doped active layer [152]. The solar cells under illumination showed enhancement in the conversion efficiency with a magnitude of 5 orders in comparison to those solar cells without Mn doping in the active layer. This large enhancement in the solar cell's conversion efficiency is because the Mn introduces energy levels below the GaN's bandgap. These energy levels take part in the absorption of sub‐bandgap photocurrent. The experiment done on Mn in GaN showed that Mn's impurity level could be formed at the center of GaN's energy gap. This can be a fact that doping in GaN alloys can enhance the photocurrent and can be suitable for photo supercapacitors [153, 154].

### 7.3 Defect Engineering and Surface States in GaN for Energy Storage

Defect engineering is one of the most significant approaches to modulating electrochemical behavior in semiconducting materials [155, 156]. Doping and surface state have a potent effect on the electrochemical behavior of Gallium Nitride (GaN) by completely changing the electronic structure, carrier density, surface energy, and adsorption properties of the material. The electrochemical activity of GaN is highly sensitive to its defect chemistry, and this can be effectively controlled to maximize its properties in multiple applications, such as photo‐assisted supercapacitors and sensing [157]. Some of the defects observed in GaN include intrinsic defects, extrinsic defects, vacancies, interstitial defects, doping defects, as well as dislocation defects [158], whose importance in the determination of the electrochemical properties of a material is apparent since the defects influence the modulation of the bandgap of semiconducting materials as well as the electrochemical charge transfer between the electrodes and electrolytes in a supercapacitor [159]. However, in pristine GaN, the primary properties include low intrinsic conductivity with high ionic binding, but with suitable defects, the electrochemical properties can be significantly improved [160].

Natural defects in GaN include nitrogen vacancies (V_N_), gallium vacancies (V_Ga_), as well as the presence of atoms occupying interstitial positions. These function as acceptors as well as donors, depending on the type of defect. In this respect, nitrogen vacancies are of immense importance since they form shallow donor levels near the bottom of the conduction band in Gallium nitride, thereby increasing the overall n‐type conductivity of the material. Gallium vacancies act as acceptors; as a result, they possess the ability to cause any compensatory effects [161]. The concentration of the defects is of significant importance in adjusting the position of the Fermi level as well as the charge transport phenomenon. Control of defect chemistry is beneficial in achieving semiconducting to metallic transition states, thereby increasing ion diffusion as well as charge storage properties in supercapacitor electrodes. Calculations of first‐principles density functional theory (DFT) prove that V_N_ is energetically preferred to gallium vacancies (V_Ga_) in bulk and nonpolar (1010) surfaces, both in the case of a Ga‐rich environment and surfaces with a N‐rich environment [162]. The stable 3+ charge state of V_N_ within the bandgap significantly influences Fermi‐level positioning and electrical transport.

**FIGURE 8:**
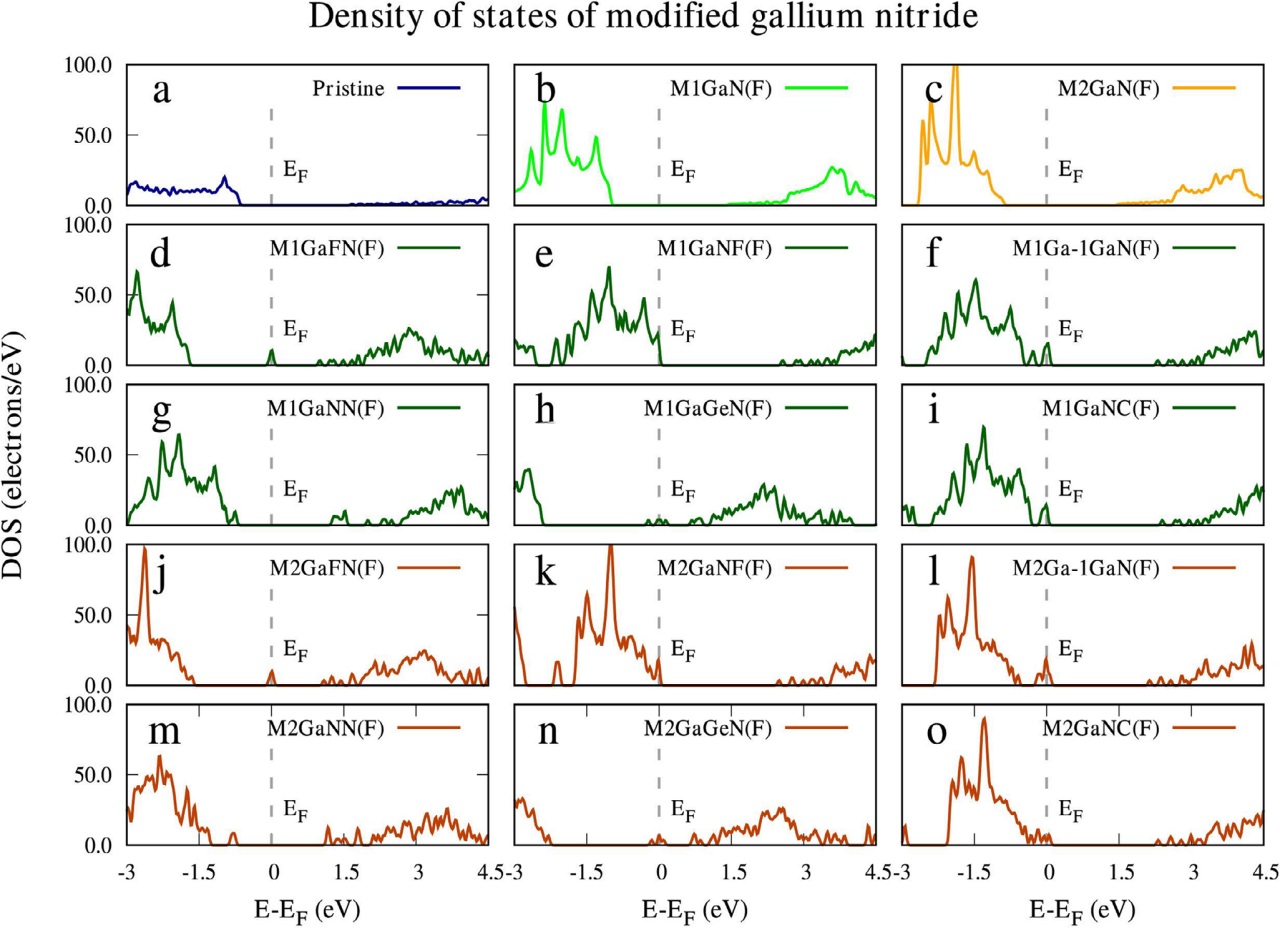
Density of states (DOS) of pristine and modified GaN monolayers showing the effects of fluorine substitution, co‐doping with Ge and C, and vacancy creation on electronic states near the Fermi level (E‐E_F_) (Reproduced with permission from ref. [164] Copyright 2024 Elsevier).

Extrinsic doping is another way that allows greater flexibility in modifying the electrochemical properties of GaN. Si, Mg, as well as oxygen, in addition to varying the charge carrier concentration, have been employed as a dopant. Controlled doping offers a critical approach that helps to control these inherent characteristics. Silicon (Si) donors increase n‐type conductivity, and magnesium (Mg) acceptors increase p‐type behavior, but may change the formation energies of gallium vacancies and complexes simultaneously [163]. Incorporation of dopant may alter the band structure, may introduce impurity levels, and may alter the Fermi energy level and allowing the electrical and optical properties of the material to be tuned selectively. Theoretical models in Figure 8 show that substitution of fluorine and vacancy and co‐doping with Ge atoms or C atoms have some major perturbations in the projected density of states, i.e., a greater band hybridization and a better level of carrier delocalization [164]. Using transition metals as a dopant allows the creation of localized states having redox characteristics, termed redox‐active sites in the literature [165]. These redox‐active sites can be employed, in addition to the capacitance contribution of the conventional EDL, to provide additional charge storage. These systems in particular demonstrate greater charge transfer resistance, specific capacitance, as well as rate performance properties than undoped GaN. Moreover, in instances of co‐doping, the presence of a vacancy‐co‐dopant pair has been observed to induce certain defect structures in particular, showing greater charge–discharge reversibility.

The direct effect of carrier density modulation caused by defects and dopants is on the electrochemical kinetics of GaN. Vacancies of nitrogen increase the density of electrons and charge‐transfer ability, and the negatively charged V_Ga_ can decrease the concentration of free carriers or add compensating acceptor states [166]. Interestingly, some charged V_Ga_ states are reported to enhance electrical conductivity six times, which demonstrates the complexity of defect‐charge‐state interactions. The surface‐sensitive investigations indicate that the chemical etching and plasma treatments significantly change the surface stoichiometry and GaN/electrolyte interface, which alters the surface energy and reactivity [167].

Electrochemical properties of GaN are surface‐sensitive, where surface states, characterized by the presence of dangling bonds, adsorbates, or surface reconstruction, are important in controlling the density of the active sites. Moreover, in the wurtzite phase of GaN, the (0001) planes as well as the (000‐1) planes, which possess the spontaneous polarization field, could alter the adsorption of ions [168]. Nevertheless, in the wurtzite phase of GaN, the natural polarization field assists in charge separation, thereby enhancing charge transfer kinetics. Moreover, the introduction of surface defects in GaN electrodes by employing plasma treatment, annealing, and chemical etching techniques can increase the electrolyte wettability in GaN [169]. Using rGO, conducting polymers, as well as transition metal oxides as electrodes, in combination with GaN, the efficiency of charge transfer in the combination is also surface‐sensitive and defect‐dependent in the composed interface [7, 170]. Moreover, in the interface, defects induce band bending as well as a built‐in electric field that assists in the flow of electrons in a mechanical environment. Using defect‐based charge transfer properties in GaN/rGO [171], Au/rGO/GaN [172], and GaN/PANI [173] hybrids can enhance the conductivity of the hybrids. In addition, the oxygen‐rich surface of GaN enables high‐level contact between GaN and metal oxides, eventually achieving a well‐synced pseudocapacitive property.

### 7.4 Theoretical and Computational Insights (DFT and Molecular Simulations) GaN‐Based Electrodes for Supercapacitors

**FIGURE 9:**
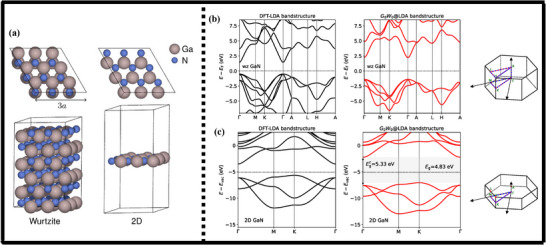
(a) Side and Top visualization of the supercell of hexagonal GaN (left) and slab model of the two‐dimensional GaN (right), calculated band structures of (b) bulk wurtzite (wz) GaN and (c) two‐dimensional (2D) GaN using DFT–LDA and GW@LDA methods (Reproduced from ref. [175] Copyright MDPI, 2023).

The Density functional theory (DFT) and its associated many‐body solutions can be used to provide a predictive approach that correlates atomic‐scale defects in GaN to macroscopic electrochemical properties. First‐principles studies consistently show that nitrogen vacancies (*V_N_*) are thermodynamically favored over gallium vacancies (*V_Ga_*) in bulk and at the nonpolar $\left(10\bar{1}0\right)$ surface, where *V_N_* acts as a shallow donors that raise electron density and facilitate charge transfer [162]. Energies of formation of defects are highly dependent on Fermi‐level position, establishing stable charge states and equilibrium concentrations; this dependence has been established on wide‐gap gallates (e.g., LiGa_5_O_8_) to support Fermi‐level engineering plans of GaN electrodes [174]. In addition to bulk, dimensional confinement provides additional information to the electronic structure: GW calculations indicate an indirect K→ Γ quasiparticle gap of ∼4.83 eV in 2D GaN, ∼1.24 eV wider than bulk (as shown in Figure 9), involving thickness‐controlled band alignment to store charge photo‐assist [175].

At electrolyte interfaces, DFT converts adsorption structures, charge transfer, and interfacial band bending, which controls ion uptake and ion kinetics. Gallium adsorption on GaN (0001) is stabilized in a Fermi‐level dependence on ammonia‐rich conditions in a manner directly proportional to surface carrier density and, consequently, to the formation of two layers on the surface and to pseudocapacitive reactions [176]. Calculations of the electronic structure of Ag‐modified hexagonal monolayers of Ag‐based materials display dopant‐induced states (Ag 5s/4p hybridization with N 2p and Ga 4d), redistributing the density of states around *E_F_* predicting reduced charge‐transfer barriers and increased interfacial reactivity ‐design scalable to supercapacitor operation [177]. In the case of hybrid structures, the models based on DFT describe the role of rGO scaffolds in supporting percolation channels of photocarriers out of GaN nanoparticles and inhibiting recombination, which is consistent with experimentally established capacitance and rate capability enhancements in rGO‐GaN hybrid [19]. In impedance and cycling measures, convergence is observed in the computation‐experiment convergence. DFT‐derived surface‐state densities are consistent with electrochemical indicators of Fermi‐level pinning and charge‐transfer resistance in aqueous conditions, and plasma or chemical interventions that alter the surface stoichiometry and defect populations result in predictable changes of band bending and Schottky behavior [157, 178].

In hierarchical and porous networks of conducting polymers and carbons in multicomponent electrodes, interface charge redistribution at GaN/graphene/polymer interfaces is predicted through DFT, explaining the low interface resistance and long‐term cyclability of flexible rGO/PEDOT/PANI systems [179]. Taken together, these computational lessons, such as defect energetics versus *E_F_* Dimensionality‐controlled band structures and atomistic adsorption/PDOS investigations, allow optimization of GaN‐based supercapacitor electrodes by mechanism control, dopant selection, control over surface conditioning, and heterointerface design in terms of high power and light‐sensitive operation.

## 8 Surface Functionalization and Interface Engineering of GaN for Enhanced Charge Storage

Surface functionalization and interface engineering are two important ways to improve the electrochemical properties of wide bandgap semiconductors like gallium nitride [180, 181]. Because of its intrinsically poor electrical conductivity and low electrochemical activity, pristine GaN needs surface modification to enhance charge transfer, ion accessibility, and active site density [182]. Through tailored surface chemistry and engineered heterointerfaces, significantly optimized electrochemical performances of GaN‐based electrodes have been realized toward high‐performance supercapacitors, hybrid capacitors, and photo‐assisted energy storage devices [7, 26, 183, 184]. Surface functionalization seeks to attach chemical groups, defects, or nanostructured coatings to change surface electronic states and interfacial compatibility of GaN with the electrolyte. These modifications may improve surface polarity, electrolyte wettability, and ion diffusion, increasing the efficiency of charge storage. For example, hydroxylation or oxygen‐rich functionalization of GaN surfaces improve hydrophilicity, enhancing ion transport and improving electrolyte penetration. Besides, functional groups act as anchoring sites for subsequent hybridization with conductive or redox‐active materials, thereby enabling efficient charge transfer at the electrode‐electrolyte interface.

### 8.1 Interface Engineering with Conductive Additives

Interface engineering involves the construction of heterojunctions between GaN and materials that have high electrical conductivity, such as graphene, carbon nanotubes (CNTs), conducting polymers (e.g., Polyaniline or Polypyrrole), and transition metal oxides. The interface helps in creating an internal electric field, which promotes charge separation and electron transfer. For example, in the GaN/rGO composites, the *π*–*π* interactions between the layers of graphene and the GaN nanoparticles create a high electron transport path while maintaining the integrity of the composite. Also, in the GaN/PPy and GaN/PANI interfaces, the electric double‐layer and pseudocapacitive contributions result in high areal and volume capacitances.

Functionalization of the GaN surface can also be conducted by plasma treatment, acid etching, or atomic layer deposition. Indeed, oxygen plasma treatment, in addition to forming Ga─O bonds, increases the surface reactivity and suppresses charge traps due to defects. Furthermore, ultrathin layers of Al_2_O_3_ or TiO_2_ deposited by ALD can be utilized as surface protection layers, which do not degrade the quality of the GaN surface even during electrochemical redox cycles [185, 186]. Moreover, they also suppress charge recombination. Hence, enhancement of the GaN surface, along with its protection against chemical corrosion in aqueous solutions, must be accomplished with caution.

### 8.2 Photo‐Active Interface Design for Hybrid Supercapacitors

Being a semiconductor, it exhibits high levels of photo‐resistivity when exposed to UV illumination, hence applicable for charge storage and photocatalysis [187]. The modification of the interface with photosensitization/conductive materials makes way for the exploitation of the photon energy available from the GaN electrodes, taking advantage of an added electrochemical capacity [188]. The photosensitivity of the material gets highly boosted by utilizing metal oxide nanoparticles, along with graphitic carbon, for instance. Upon illumination, the photosensitive charge generated in the GaN and GaN‐based composites resides in the conducting region and gets involved in redox reactions, hence contributing differently to the photo supercapacitance [189, 190]. One of the major issues in GaN‐based composites regards the stability of the interfaces during repeated electrochemical cycles. Variations in lattice constants, surface energy, or chemical properties between GaN and other compounds might cause peeling off or deteriorating interfaces. Therefore, optimization of the interface is a multifaceted task that may involve strain relaxation, binding, and thickness of the coating layer. MLD and amine/carboxyl functional group matching are effective ways of forming high‐quality interfacial bonds. As a result of optimizing the interface, not only is the disintegration of the structure avoided, but a high charge transfer resistance as well as a high specific capacitance is ensured over a high number of electrocyclic processes.

### 8.3 Chemical Kinetics in GaN Electrodes

To evaluate the rate‐determining steps, capacitive effect of an electrode, and the nature of charge storage in GaN‐based electrodes, a power‐law analysis is typically employed. The relationship between the measured current (*i*) and the scan rate (*v*) is expressed by the following equation [191, 192, 193]:

(1) $$i=av^{b}$$

where *a* and *b* are adjustable parameters. The b‐value is particularly instructive: the value of *b* = 0.5 indicates a diffusion‐controlled process typically characteristic of battery‐like intercalation, whereas *b* = 1 signifies a purely surface‐controlled capacitive nature of the electrode [193]. For GaN nanostructures, such as porous GaN or GaN nanowires, the b‐values reported in the literature often reside in the range of 0.70 to 0.95 [26]. This indicates that while the system is not purely EDLC‐based, the charge storage is dominated by fast surface‐controlled pseudocapacitance [194]. This is attributed to the high surface area of the GaN nanostructures and the efficient charge transfer enabled by the wide‐bandgap semiconductor's electronic properties. Furthermore, the contribution of the capacitive current (*k_1_v*) and diffusion‐limited current (*k_2_v* ^1/2^) can be quantified using the following relationship [195]:

(2) $$i\left(v\right)=k_{1}v+k_{2}v^{1/2}$$

Here, *k*_1_ *v* and *k*_2_ *v* ^1/2^ correspond individually to the capacitive and diffusive contributions to the total charge storage at a definite potential and given rate. By deconvolving these contributions, it becomes evident that the high‐rate performance of GaN is a direct result of the dominant *k_1_* component, allowing for rapid ion adsorption/desorption and fast surface redox reactions even at high scan rates. This kinetic profile distinguishes GaN from conventional bulk metal oxides, which often suffer from slower diffusion‐limited processes.

## 9 Preparation Methods of Gallium Nitride

Gallium Nitride is available in many forms, which are powder, nanowires, and thin films, and each has its own application in energy storage. The synthesis of GaN in such various shapes requires particular synthesis methods, as each of them has its own strengths and difficulties. Researchers focus on improving efficiency, purity, and overall performance and are always willing to extend the limits of GaN. By enhancing these techniques, they can access the potential of GaN in challenging uses in energy storage and optoelectronics and make full use of its impressive thermal, electronic, and optical characteristics.

### 9.1 Gallium Nitride Nano‐Powders

**FIGURE 10:**
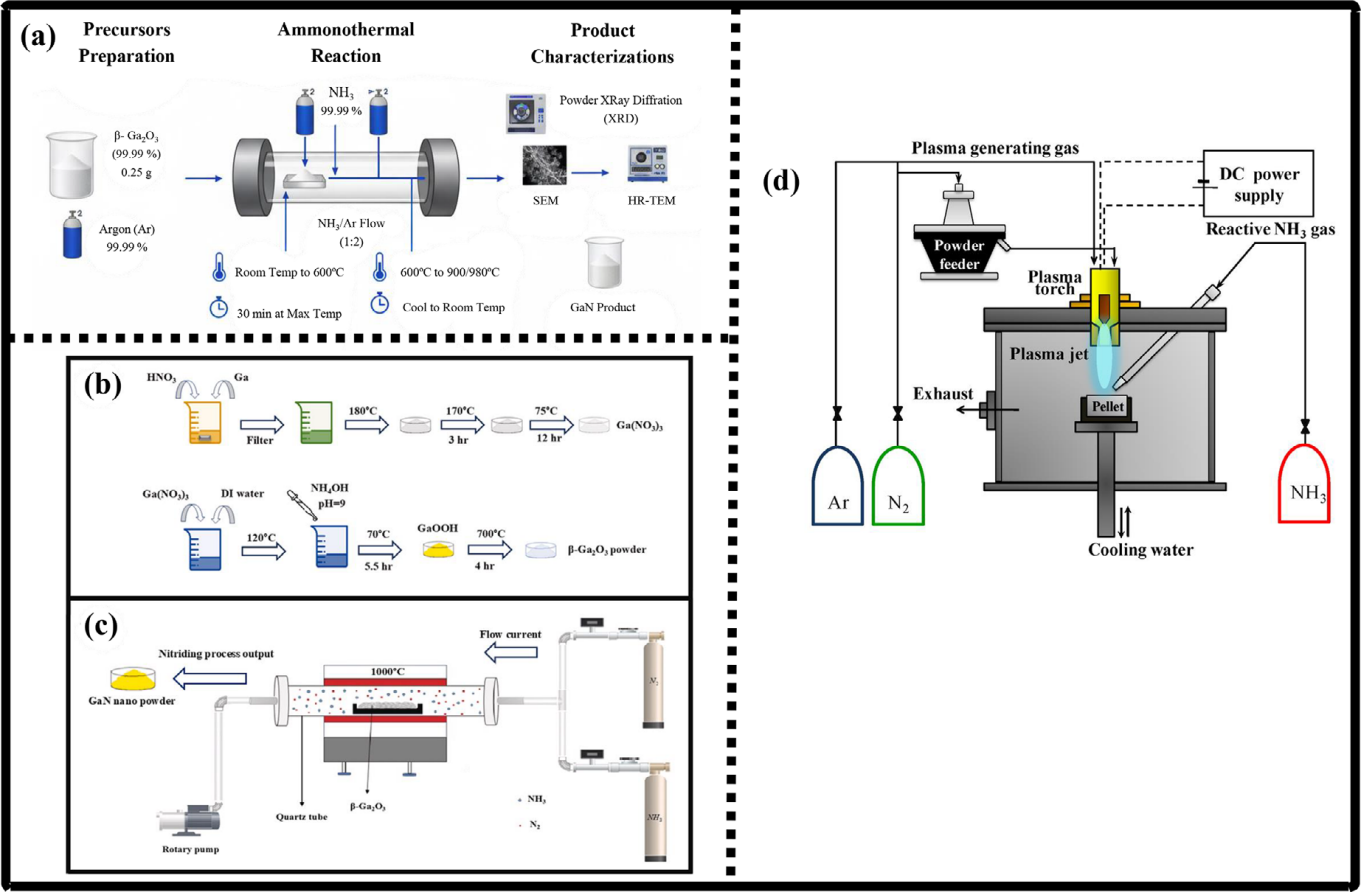
(a) Ammonothermal reduction nitridation method, (b,c) GaN Nano powder synthesis via nitridation (Reproduced from ref. [197], Copyright Elsevier 2025) (d) Thermal plasma synthesis of crystalline gallium nitride nanopowder (Reproduced from ref. [199] Copyright MDPI 2016).

Researchers have experimented with a wide variety of techniques for synthesizing gallium nitride (GaN) powder, each presenting its own distinct advantages and limitations that shape its suitability for different applications. Kang et al. employed an ammonothermal reduction‐nitridation method to convert gallium oxide to gallium nitride [196] as illustrated in Figure 10a. In it, β‐Ga_2_O_3_ is heated in the ammonia atmosphere under feverish temperatures—usually between 900°C and 980°C—and mixes with NH_3_ to give GaN. Ammonolysis under such high temperatures is not exceedingly difficult and can be made on a large scale to manufacture large quantities of ammonia. The process, however, has significant flaws: the conversion of Ga_2_O_3_ to GaN is not always complete, and the remaining oxide and the rest of the oxygen are still present in the product, which can also facilitate decomposition and sublimation of GaN. To reduce the oxygen content, high temperatures (950°C–975°C) are required. Consequently, gallium nitrate powders prepared at high temperatures retain some impurities of Ga_2_O_3_ (4.5–2.9 wt.% oxygen), but further nitridation at ≥ 975°C enhances purity but raises the crystallite size and results in more material losses. Incomplete nitridation of gallium oxide is a significant problem, which can also often lead to the production of GaN of unsatisfactory purity. Chemical and incomplete precursors may interfere with the electronic and optical characteristics of the resulting powder, which is a major concern for high‐performance applications.

Conversely, the carbothermal reduction nitridation process incorporates carbon as a catalyst to promote the formation of GaN crystals [197]. Such an approach was optimized by Mahdi et al., a multi‐step synthesis, which led to great improvements in the purity and the structural integrity of the obtained GaN, as in Figure 10b,c. These improvements are especially important to optoelectronic devices and power electronics, with material quality directly affecting the efficiency and reliability of the devices. Moreover, Mahdi introduced a new path of the production of gallium nitride nanoparticles: once the process of nitrifying the substance is completed, the nanoparticles are transferred to the silicon substrates through electron beam evaporation. The method not only provides nanoparticles with the desired properties but also allows the incorporation of GaN with silicon technologies, which is quite beneficial when we need to perform such tasks as ultraviolet (UV) detection, in which case we need materials that are sensitive and stable.

In a radically different approach, the group of Liu combined the sol‐gel process with high‐temperature ammoniation with gallium ethoxide as the starting material [198]. The sol‐gel method has provided excellent control over the chemical composition and particle size, and further ammoniation guarantees an effective transformation of the sol‐gel into GaN via high‐temperature ammoniation, in which Ga(OC_2_H_3_)_2_ can be used as a starting substance. This process is unique in that it is innovative, with a high level of efficiency that it introduces in the process of synthesizing GaN powder. Using the strengths of both sol‐gel chemistry and thermal processing, Liu's team manufactured GaN powders, which demonstrate uniformity and high purity, and are thus suitable for use in highly technological applications where consistency of the material is ranked as one of the primary requirements. Then, Kim et al. also came up with a totally different idea (Plasma synthesis) [199], as given in Figure 10d. The direct current non‐transferred arc plasma was used to obtain GaN nano powder. Their process begins with the raw material, gallium nitrate hydrate (Ga(NO_3_)_3_.*x*H_2_O), and the source of nitrogen, NH_3_ gas. They inject melamine powder directly into the plasma flame to avoid oxidation of gallium to Ga_2_O_3_.

### 9.2 Gallium Nitride Nanowires

**FIGURE 11:**
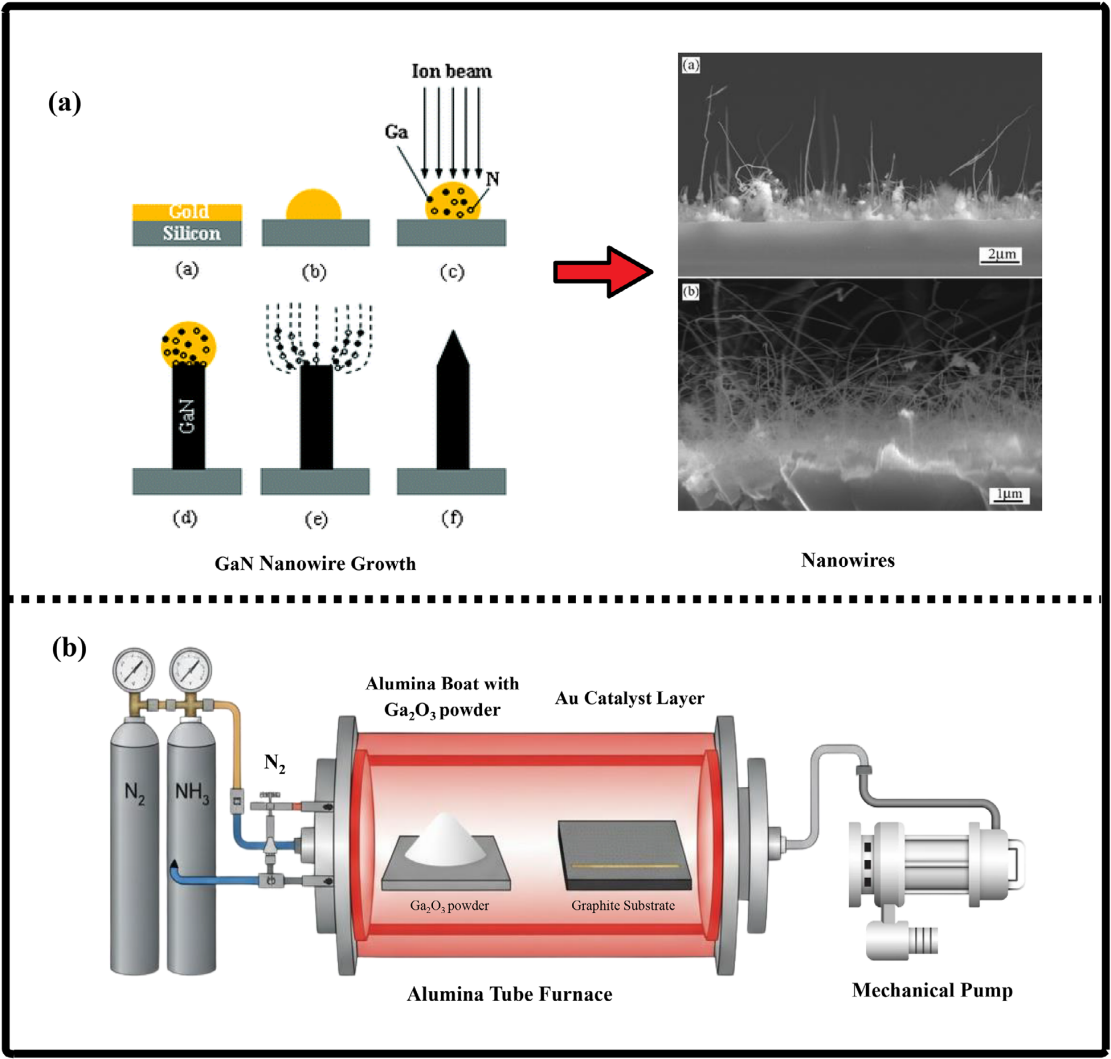
Synthesis of GaN Nanowires by (a) Plasma‐assisted method (Reproduced with permission from ref. [200] Copyright Royal Society of Chemistry 2013), (b) Scalable CVD method.

The existing synthesis methods are under constant improvement by the researchers to make gallium nitride (GaN) materials more effective, pure, and crystalline. These enhancements are essential because GaN of high quality is the basis of the improved optoelectronic and electronic usage. Recently, Wang and co‐workers came up with a simple and greener method of synthesizing the GaN nanowires [200] directly through the GaN powder, as given by Figure 11a. Their method is based on plasma‐assisted hot filament chemical vapor deposition, a process that, in addition to making the fabrication process simpler, also saves the usage of dangerous chemicals and complicated precursors. This innovation plays a major role in enabling the fabrication of GaN nanowire films with favorable optical and electrical characteristics, and thus, it is more easily available to be incorporated into devices like LEDs, lasers [201], and high‐frequency transistors.

At the same time, the research group of Kumaresan investigated a different approach, which is the direct growth of GaN nanowires upon graphene substrates [202], as depicted in Figure 11b. Graphene, a highly conductive material with high mechanical strength, has been suggested as a perfect platform on which GaN nanowires can be grown vertically. The potential of this hybrid form is vast in advancing the electronics devices of the coming generation, which may result in circuits that are much faster and more efficient, and more sensitive sensors. The smooth junction between the GaN and graphene may provide special architectures of devices that may exploit the optimal properties of the two substances.

During another creative practice, Sun and collaborators proposed a synthesis mechanism aimed at improving the electrochemical property of the GaN nanowires [203] especially in energy storage systems. Their interest is related to the attainment of stable and tunable lithium storage in GaN nanowires, but with impressive pseudocapacitance properties. This implies that the nanowires are capable of holding and discharging vast quantities of charge very quickly and are therefore very appealing to be used in next‐generation batteries and supercapacitors. After optimizing the synthesis conditions, the team of Sun was able to show that GaN nanowires cannot only be very stable but also vary in storage capacity, which preconditions their application in more innovative technologies in energy storage. All these various studies are pushing the limits of what is possible with GaN nanowires, and new possibilities in electronics, photonics, and energy storage have become possible.

### 9.3 Gallium Nitride Membranes and Films

**FIGURE 12:**
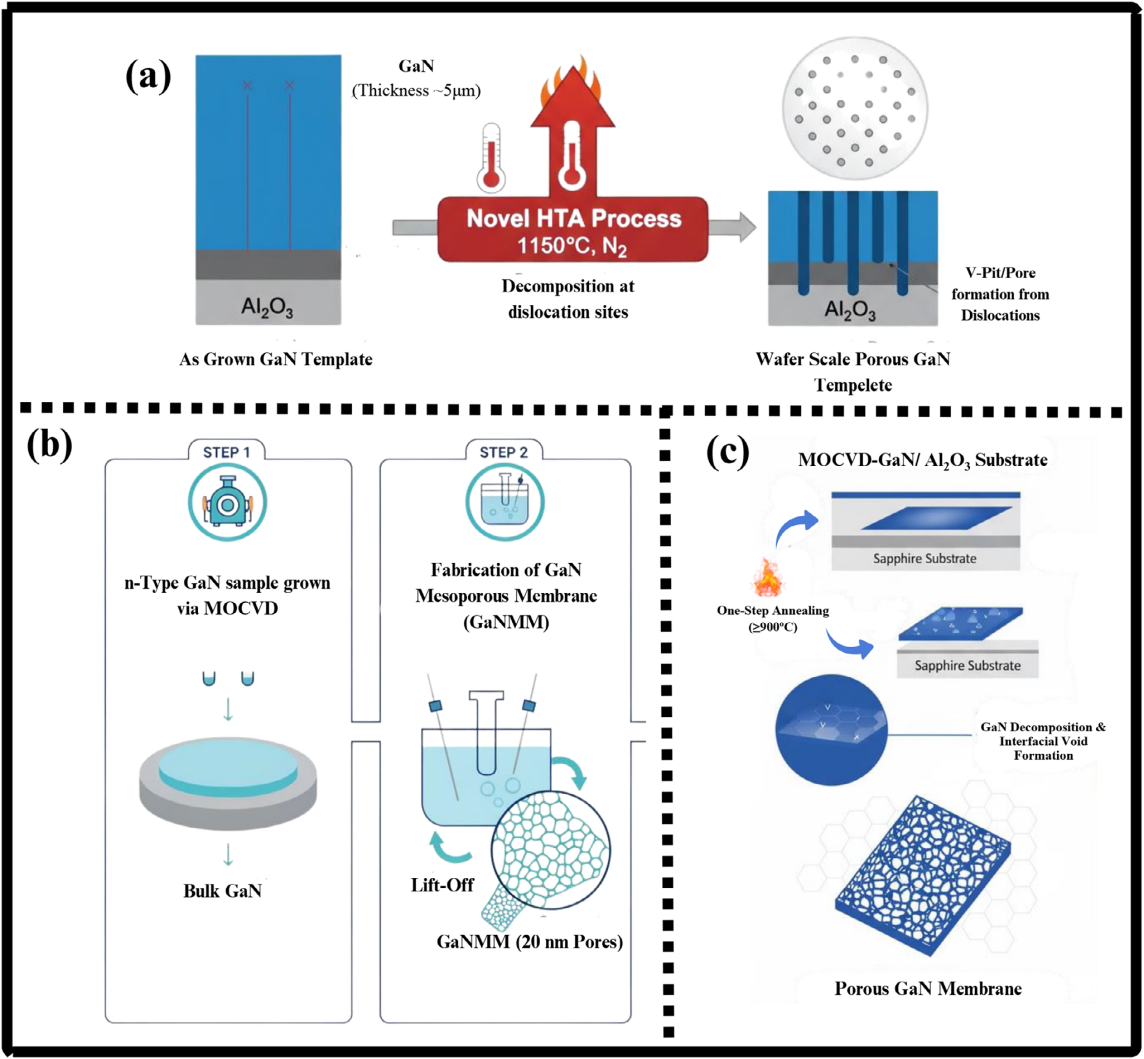
Synthesis of GaN (a) wafer scale porous GaN, (b) GaNMM grown via MOCVD, (c) porous GaN membrane on sapphire substrate.

In developing a new electrochemical etching method to synthesize single‐crystal gallium nitrate mesoporous membranes (GaNMM), Wang et al. introduced a breakthrough in the science [204]. By initially using the metal‐organic vapor phase etching (MOVPE) to produce high‐quality n‐type gallium nitride layers, they prepared the groundwork for a two‐step etching process, as given in Figure 12b. This allowed the lift‐off of single‐crystal GaNMM‐20 films to occur successfully, and they have structural integrity and a desirable porosity. The development of these membranes creates unprecedented opportunities for the scalable production of GaN sheets and films that are important elements in advanced optoelectronics and energy storage devices because they have better electronic and thermal properties.

Alternatively, Yu et al. proposed a simpler but very efficient high‐temperature annealing method to produce porous substrates of GaN on a scale of a wafer [205], as illustrated in Figure 12a. In addition to the ease of the fabrication process, the process enables the porosity and crystallinity of the resulting GaN structures to be precisely controlled. With these characteristics, which can be tuned by the researchers, the electronic, optical, and mechanical properties of GaN sheets and films can be optimized to apply in certain energy storage processes. Such parameters can also be increasingly optimized by using the high‐performance supercapacitors and batteries, whose surface area and conductivity of the electrode materials are crucial factors.

At the same time, the possibilities in this area were also extended by the research group of Zhang, who developed a one‐step high‐temperature annealing procedure to prepare single‐crystalline porous films of large area made of free‐standing GaN [129], as designed in Figure 12c. Their lean design not only maximizes their efficiency but also makes them scalable and reproducible enough to be used in industry. The resulting films of GaN are specifically applicable to integration into future energy storage devices, where their combination of mechanical strength, chemical stability, and tunable porosity can be used to benefit from high energy densities and enhanced cycling.

## 10 Electrochemical Performance of GaN

Gallium Nitride (GaN) is rapidly emerging as a front‐runner in the development of next‐generation supercapacitors, offering a compelling blend of high specific capacitance, robust rate capability, and exceptional long‐term cycling stability. The unique properties of the material due to its large bandgap, breakdown voltage, and large thermal conduction make GaN an exceptionally attractive candidate in the context of energy storage. At the nanoscale, by controlling the architecture by forming porous geometries of GaN or vertically aligned nanorod arrays or combinations with high‐surface‐area structures, the researchers can significantly raise the amount of electroactive interface they have available to store charge. Doping of GaN with other elements, such as magnesium, is not only able to tune its electronic characteristics but also adds more active sites, increasing its pseudocapacitive response even more. Further, the development of GaN‐based composites based on the association of GaN with metal oxides, conductive polymers, or carbonaceous materials takes advantage of the synergistic effect, leading to even greater specific capacitance and enhanced charge transport.

Among other things, excellent durability is one of the most distinguishing features of GaN‐based materials used in supercapacitors, which is what is of the utmost importance when it comes to the real‐life use of the device. Supercapacitors are frequently put through tens of thousands of fast cycles of charging and discharging, and in this case, GaN really pays off. Indicatively, electrodes made of porous GaN or hybrid gated nitrogen‐doped carbon in the form of honeycomb structures have shown that they were able to retain close to 100 % of their original capacitance even after 10 000 cycles. This retention is much higher than that of the traditional electrode materials, which highlights the strength of GaN. The structural strength of GaN and the chemical strength testify to the fact that, even in high current density, pure GaN electrodes prepared by electrochemical etching have maintained 99 % capacitance following 50 000 cycles. This has been achieved to a large extent by the high covalent bonds of use and the resistance of structural degradation of GaN, which, when put together, provide a defense against the physical and chemical strains that can be exhibited during the riding on aggressive riding.

High n‐type doping, typically achieved with silicon, improves electrical conductivity and decreases charge transfer resistance R_ct_, leading to higher power and energy densities [206]. On the other hand, p‐type doping, typically with magnesium, has increased resistivity and poorer hole mobility, resulting in poor electrochemical performance [207, 208]. The ESR is the sum of the GaN electrode's bulk resistance, the electrolyte resistance, and R_ct_. These factors significantly impact the performance of GaN‐based supercapacitors. One of the main ways to reduce total ESR is by increasing the carrier concentration, which has the effect of lowering bulk resistance and R_ct_. Another way to achieve high power density is through fast discharge, which enables the device to handle high current densities without a significant IR drop (voltage loss) [209].

To investigate the intrinsic electronic properties of the GaN electrodes, Li et al., [210] designed a new photoelectrochemical (PEC) photosensor made of GaN nanowires on a Si substrate, which had a record‐high responsivity of 247.8 mA/W and was extremely stable. Mott‐Schottky offered useful information by examining the linear portion of the fitted curve, where the slope indicated the relative magnitude of the donor concentration and the intercept represented the flat‐band potential. He found that the amount of the positive slope in the Mott‐Schottky curves closely correlates with the relative donor concentration in the material, with lower slope values indicating larger donor concentrations. Moreover, he investigated the electrical characteristics of the photoelectrode using electrochemical impedance spectroscopy (EIS), which indicates the efficiency with which charge carriers transfer in the photoelectrode. Previous research has consistently proven that a smaller arc radius in Nyquist plots corresponds to a lower charge transfer resistance (R_ct_) within the photoelectrode [211]. This investigation showed that the R_s_ values of the three kinds of p‐n GaN nanowires were almost comparable, confirming the accuracy of the fitting findings.

Not only is durability concerned with cycle life, but even electrodes that are made using GaN have the potential to maintain high capacitance under severe operating conditions. GaN composite including NiCoO_2_ can retain over 80 % of its initial capacitance even under extreme conditions such as high temperature or high current density. This extreme flexibility is not only something that can expand the scope of application of the GaN‐based supercapacitors but also highlights how flexible and reliable the material can be in the real‐life situation of energy storage needs. An overall analysis of these diverse material structures, as obtained in the literature and in performance charts, provides useful information on what to explore most of all in GaN‐based systems, and paves the way for future studies in that direction.

Specific capacitance, as the direct measure of the ability of materials to store charge, is critically dependent on the inherent qualities of GaN as well as the complexity of its construction design. Typical pure GaN electrodes have a specific capacitance of 21.22 to 52.58 cm^−1^ between electrodes, depending on the synthesis schemes and test conditions. Nevertheless, inclusion of pseudocapacitive substances, e.g., transition metal oxide, or inclusion of highly conducting additives can increase these numbers significantly. As an illustration, composites based on GaN/MnO_2_/MnON composites have achieved specific capacitances as high as 1915.5 mF cm^−2^, while GaN/Ga_2_O_3_ hybrids have reached 1301.2 mF cm^−2^. These advances are motivated by the synergizing processes involved: GaN is offering a stable double‐layer capacitance base, whereas the redox‐active components involved in the integration are offering faradaic charge storage, thus leading to an overall synergistic functionality. In more recent architectural designs, rGO‐GaN nanocomposites and honeycomb GaN networks also take advantage of large accessible surface areas and rapid ion and electron transport, resulting in very high specific capacitance values such as 454 F g^−1^ and 730 mF cm^−2^, respectively.

The values of energy density and power density are also of importance to practical applications as they define the quantity of energy that can be stored and the rate at which it can be provided. Electrodes based on GaN, especially in their composite or nanoscale format, have shown comparable energy and power densities, and sometimes even better than those of conventional carbon‐based supercapacitors. An example is the GaN honeycomb/nitrogen‐doped carbon hybrid with an energy density of 3.12 mWh cm^−3^ at a power density of 714.3 mW cm^−2^, while GaN/Ga_2_O_3_ hybrids deliver 0.58 Wh kg^−1^ (3.54 mWh cm^−3^) at 154 W kg^−1^ (0.94 W cm^−2^). These great numbers highlight the two‐fold benefit of the GaN‐based electrodes; not only could the electrodes accumulate a large volume of energy, but also allow a high charge and discharge rate, as listed in Table 7. The key to the success of such systems is the careful design of the electrodes: to increase ionic accessibility, to create interconnected networks of porous structures that allow rapid transport, and to combine the electric double‐layer with the pseudocapacitive mode of charge storage, which is multifaceted.

**TABLE 7:** Electrochemical performance of GaN‐based materials for supercapacitors.

Working electrode	Preparation method	Reaction conditions	Cyclic stability	Specific capacitance	Energy density and Power density	Refs.
GaN/GP	Chemical vapor deposition (CVD) method	CE: Pt Sheet; WE: graphite paper; R.E: Hg/Hg_2_SO_4_; Electrolyte: 1 M H_2_SO_4_	98 % (10 000)	237 mF cm^−2^ (0.1 mA cm^−2^)	0.30 mWh cm^−3^ at 1000 mW cm^−3^	[212]
GaN Crystals	Electrochemical etching	CE: Pt Sheet; WE: Stainless Steel cloth; R.E: Hg/Hg_2_SO_4_	99 % (50 000)	23.67 mF cm^−2^ 0.01 V s^−1^)	0.65 µWh cm^−2^ at 45 mW cm^−2^	[204]
Porous GaN Single Crystal	Calcination	CE: Pt Sheet; WE: Stainless Steel cloth; R.E: Hg/Hg_2_SO_4_; Electrolyte: 1 M H_2_SO_4_	99 % (10 000)	21.22 mF cm^−2^ (0.1 mA cm^−2^)	0.58 mWh cm^−2^ at 45 mW cm^−2^	[205]
Porou GaN crystals Membrane	One‐step ball milling process	W.E: stainless steel cloth; Electrolyte: EMImNTf_2_	86.2 % (10 000)	52.58 mF cm^−2^ (0.8 mA cm^−2^)	13.3 mWh cm^−2^ at 67.5 mW cm^−2^	[129]
rGO‐GaN nanocomposites	Chemical reduction	CE: Platinum sheet; Electrolyte: 1 M H_2_SO_4_	75 % (950, 5 A g^−1^)	454 F g^−1^ (10 mV s^−1^)	—	[19]
Layered GaN Single crystal	Photoelectrochemical etching	CE: Platinum sheet; R.E: Hg/Hg_2_SO_4_; W.E: stainless steel cloth; Electrolyte: 1 M H_2_SO_4_	—	3.12 mF cm^−2^ (0.1 mA cm^−2^)	—	[9]
GaN/MnO_2_/MnON	GaN nanosheets: PECVD, GaN/ MnO_2_: Hydrothermal, GaN/ MnO_2_/MnON: Calcination	CE: Platinum sheet; RE: Ag/AgCl; Electrolytes: 1 M Na_2_SO_4_ and 6 M KOH	95.5 % after 10 000 cycles	1915.5 mF cm^−2^ (0.1 mA cm^−2^)	0.76 mW h cm^−3^ at 125 mW cm^−3^	[213]
GaN/Ga_2_O_3_	Hydrothermal process with high temperature nitridation	CE: Platinum sheet; R.E: Calomel electrode; WE: GaN/CC; Electrolytes: 1 M Na_2_SO_4_	77.27 % (20 000)	1301.20 mF cm^−2^ (0.5 mA cm^−2^)	0.58 Wh kg^−1^ at 154 W kg^−1^	[214]
GaN honeycombs/nitrogen‐doped carbon	Carbothermal reduction	CE: Platinum sheet; R.E: Hg/Hg_2_SO_4_; Electrolyte: 1 M H_2_SO_4_	Nearly 100 % (10 000)	(730 mF cm^−2^), and C_v_ (251 F cm^−3^) (200 mF cm^−2^)	3.12 mWh cm^−3^ at 714.3 mW cm^−3^	[194]
g‐C_3_N_4_/GaN	Ultrasonic Mixing	CE: Platinum plate; RE: Saturated Calomel; Electrolyte: 6 M KOH	83 % (10 000 cycles)	200 F g^−1^ (2 A g^−1^)	1.5 µWh cm^−2^ at 327.9 mW cm^−2^	[215]
GaN/NiCoO_2_	Hydrothermal route followed by thermal annealing	CE: Platinum plate; RE: Saturated Calomel; Electrolyte: 6 M KOH	82 % (10 000, 8 mA cm^−2^)	90.6 mF cm^−2^ (1 mA cm^−2^)	15.3 µWh cm^−2^ at 44.0 mW cm^−2^	[26]
GaN Nanowires	Chemical vapor deposition (CVD) method	Graphite layer with GaN Nanowires as Anodes; Electrolyte: 1 M LiPF_6_	486 mAh g^−1^ for 400 cycles	86 mAh g^−1^	—	[203]
GaN/Mn_3_O_4_	Electrochemical Deposition	CE: Platinum plate RE: Saturated calomel electrode WE: GaN single crystal Electrolyte: 0.1 M Mn (CH_3_COO)_2_·4H_2_O and 0.1 M Na_2_SO_4_	81.3 % after 10 000 Cycles	448.6 mF cm^−2^ at 1 mA cm^−2^	5.3 µWh cm^−2^ and 34.0 mW cm^−2^	[184]

Ultimately, the remarkable performance of GaN‐based electrodes is rooted in the material's unique electronic structure, mechanical robustness, and its ability to establish strong, stable interfaces with a wide array of functional components. These attributes, combined with the flexibility to tailor composition and morphology at the nanoscale, position GaN as a cornerstone for the evolution of high‐performance supercapacitors. As research continues to unravel new combinations and architectures, GaN‐based materials are set to play a pivotal role in the advancement of energy storage technologies, promising devices that are not only powerful and efficient but also durable and adaptable to the ever‐changing demands of modern electronics and renewable energy systems.

### 10.1 Recent Advances in GaN‐Based Energy Storage Materials

Metal oxides are important in improving the performance of GaN‐based supercapacitors mainly because they are able to provide high pseudocapacitance with reversible faradaic reactions at the electrode surface. Though it has already been observed that gallium nitrate (GaN) has better electrical conductivity and mechanical stability, the addition of metal oxides like manganese dioxide (MnO_2_) increases the total capacitance because these oxides introduce surface redox reactions that allow better charge storage and release than when using only two layers of capacitance.

**FIGURE 13:**
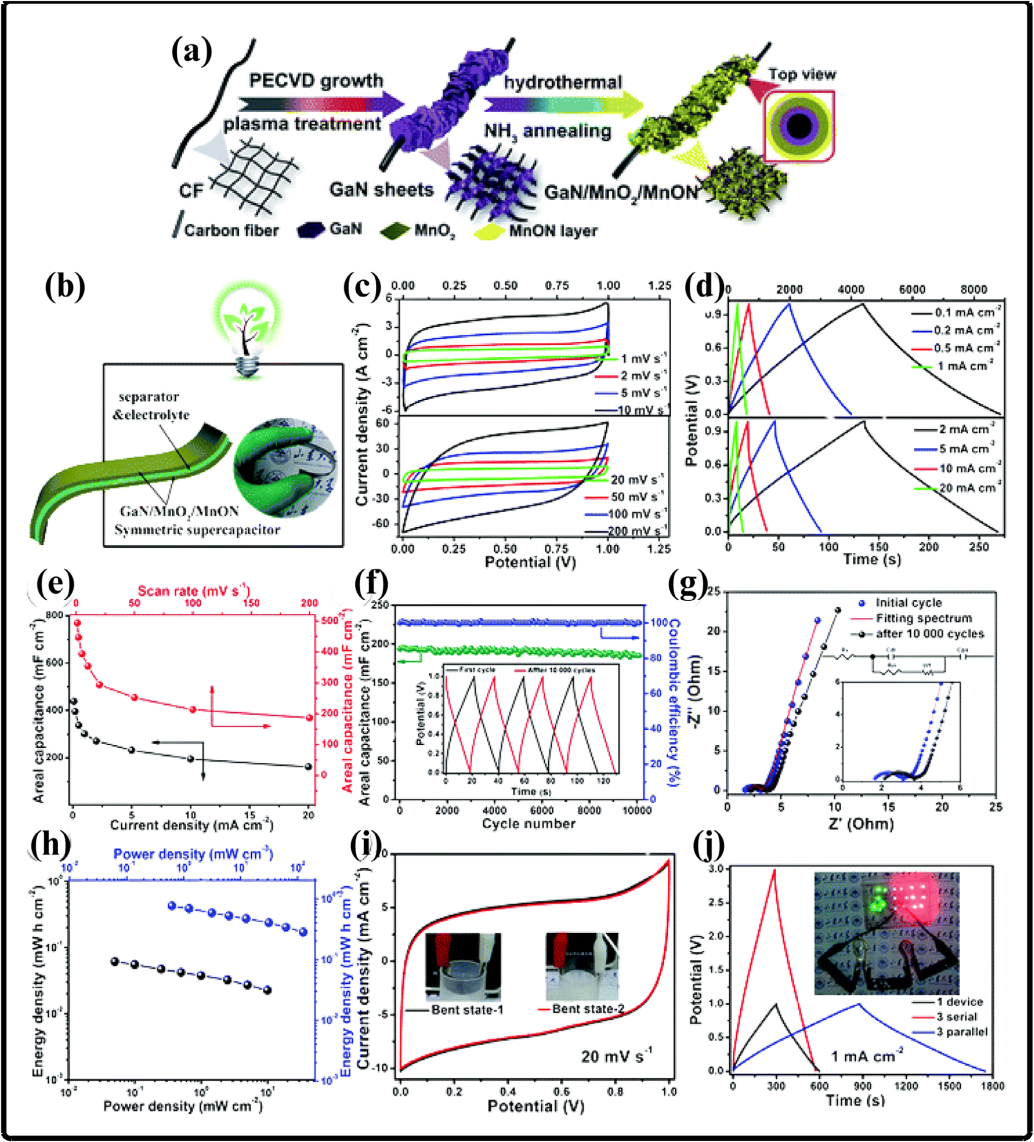
(a) Outlines how the GaN/MnO_2_/MnON complexes were grown, while (b) sketches out the design for the supercapacitor device, The inset photo shows the real, flexible supercapacitor they built, (c) presents CV analysis at several scan rates, and (d) covers GCD curves at various current densities, (e) Capacitance calculated from the CV curves, In (f) cyclic performance at 10 mA cm^−2^ again, and (g) shows the device's Nyquist plots before and after 10 000 cycles, (h) Ragone plot, (i) CV curves for the supercapacitor under different bending states at 20 mV s^−1^, (j) shows GCD curves for the devices, with an inset photo of a series of bright LEDs powered by them. (Reproduced with permission from ref. [213], Copyright Royal Society of Chemistry 2018).

Based on this combined effect, Wang et al. produced an enhanced, versatile sandwich‐type electrode architecture that comprises a systemic integration of GaN, MnO_2,_ and manganese oxynitride (MnON) [213]. Unlike traditional composites, where the layers are merely mixed or stacked up, they are designed with integrated functionality: every layer is designed to ensure that it is in direct interfacial contact with the others, directly creating a hierarchical structure with the adjacent layers. It starts with the direct development of GaN nanosheets on carbon fiber substrates by plasma‐enhanced chemical vapor deposition (PECVD), which leads to a smooth and highly conductive interface. This is then followed by the hydrothermal deposition of MnO_2_, which results in forming a uniform electroactive layer on the surface of GaN, as depicted in Figure 13a. Next, an annealing step of ammonia, leading to the formation of a coating made up of MnON that increases the redox activity and stability of the material. This high‐precision engineering forms a connected set of conductors by means of which the charge carriers are able to flow swiftly and effectively across the electrode. Notably, this architecture can also be used to solve one of the long‐term problems of metal oxide‐based electrodes, which is their propensity to mechanical degradation, such as swelling, contraction, or fracture during repeated charge–discharge cycles. The composite allows structural stability of the active materials even after a large number of cycles by ensuring close contact at the interfaces with the robust GaN scaffold, which directly leads to the longer lifespan of the devices and their increased working stability.

The research team used the three‐electrode and 2E electrochemical tests together in a combination to fully describe the properties of the material individually and also the performance of the entire device. This strict methodology allows understanding clearly how the synergistic effect of the high conductivity of GaN and the redox‐active chemistry of manganese oxides can surmount the traditional drawbacks of each constituent, that is, the low intrinsic conductivity and restricted redox activity, which has limited energy storage materials in the past. The very presence of MnO_2_ is an important contributor to the enhanced performance of the system. Because of its capacity to switch reversibly between several oxidation states (Mn^2+^, Mn^3+^, and Mn^4+^), manganese dioxide can react in a large number of redox reactions, with a high theoretical specific capacitance.

The working principle of the GaN/MnO_2_/MnON hybrid electrode is based on a fast and reversible redox reaction mechanism that takes place in the manganese oxide layers in a 6 m KOH electrolyte. GaN is electrochemically inert and does not take part in redox reactions. The charge storage behavior can be attributed to two dominating pseudocapacitive pathways. First, during discharge, potassium ions (K^+^) intercalate into the MnO_2_ tunnels, forming MnOOK, and later, during the charging process, are deintercalated, exemplified by the following equation:

(3) $$\mathrm{Mn}O_{2}+K^{+}+e^{-}⇌\mathrm{MnOOK}$$

Second, there's a proton‐coupled electron transfer, where water provides protons to MnO_2_, reducing it to MnOOH and releasing OH^−^ ions,

(4) $$\mathrm{Mn}O_{2}+H_{2}O+e^{-}⇌\mathrm{MnOOH}+OH^{-}$$

MnON, which forms right at the GaN/MnO_2_ interface, follows similar redox routes,

(5) $$\mathrm{MnON}+K^{+}+e^{-}⇌\mathrm{MnONK}$$

(6) $$\mathrm{MnON}+H_{2}O+e^{-}⇌\mathrm{MnONH}+OH^{-}$$

MnON exhibits a faster rate of reaction and increases the durability of the material by means of nitrogen doping, which increases the structural stability as well as electrical conductivity of the material. GaN does not directly participate in any redox chemistry; however, it is an extremely relevant component. It is also a channel of effective electron transport, which directs the charge carriers between the active MnO_2_ and MnON sites. Moreover, MnO_2_ is interfaced by a stable GaN, leading to the creation of a MnON buffer layer, which tolerates mechanical strains due to alterations in volume during the charge and discharge process. This interface averts electrode degradation during the long run usage.

This composite design has proven to be effective due to its performance metrics. The electrode has an exceptional areal capacitance of 1915.5 mF cm^−2^ (532.1 mAh cm^−2^) at the current density of 0.1 mA cm^−2^ in 6 M KOH, and a flexible two‐electrode device has an energy density of 0.76 mWh cm^−3^, more than seven times that of conventional supercapacitors. It is worth mentioning that the capacitance of the device remains at 95.5 % of the starting value following 10 000 cycles of charge/discharge, which proves the high stability of the device in cycling. There is also high resistance to repeated mechanical deformation of the supercapacitor, i.e., bending of the device, which does not cause a reduction in performance, so the supercapacitor can be used with flexible and wearable electronics where its ability to withstand mechanical deformations is important. The GaN/MnO_2_/MnON composite is one of the examples of how the careful choice of materials and their specific structural arrangements can resolve the drawbacks of traditional electrodes and allow creating efficient energy‐storage systems meeting the rigorous demands of next‐generation uses, including electric vehicles, renewable energy, and storage on the grid, as well as in portable consumer devices. New materials and structures of supercapacitors, including this one, should continue to be crucial in the future of energy storage as the world attempts to transition to sustainable and efficient energy solutions. Figure 13b is the illustration that the fabricated GaN/MnO_2_/MnON hybrid supercapacitor device can be used as a real‐life application, while the electrochemical performance of the GaN/MnO_2_/MnON hybrid supercapacitor device is shown in Figure 13c–j.

**FIGURE 14:**
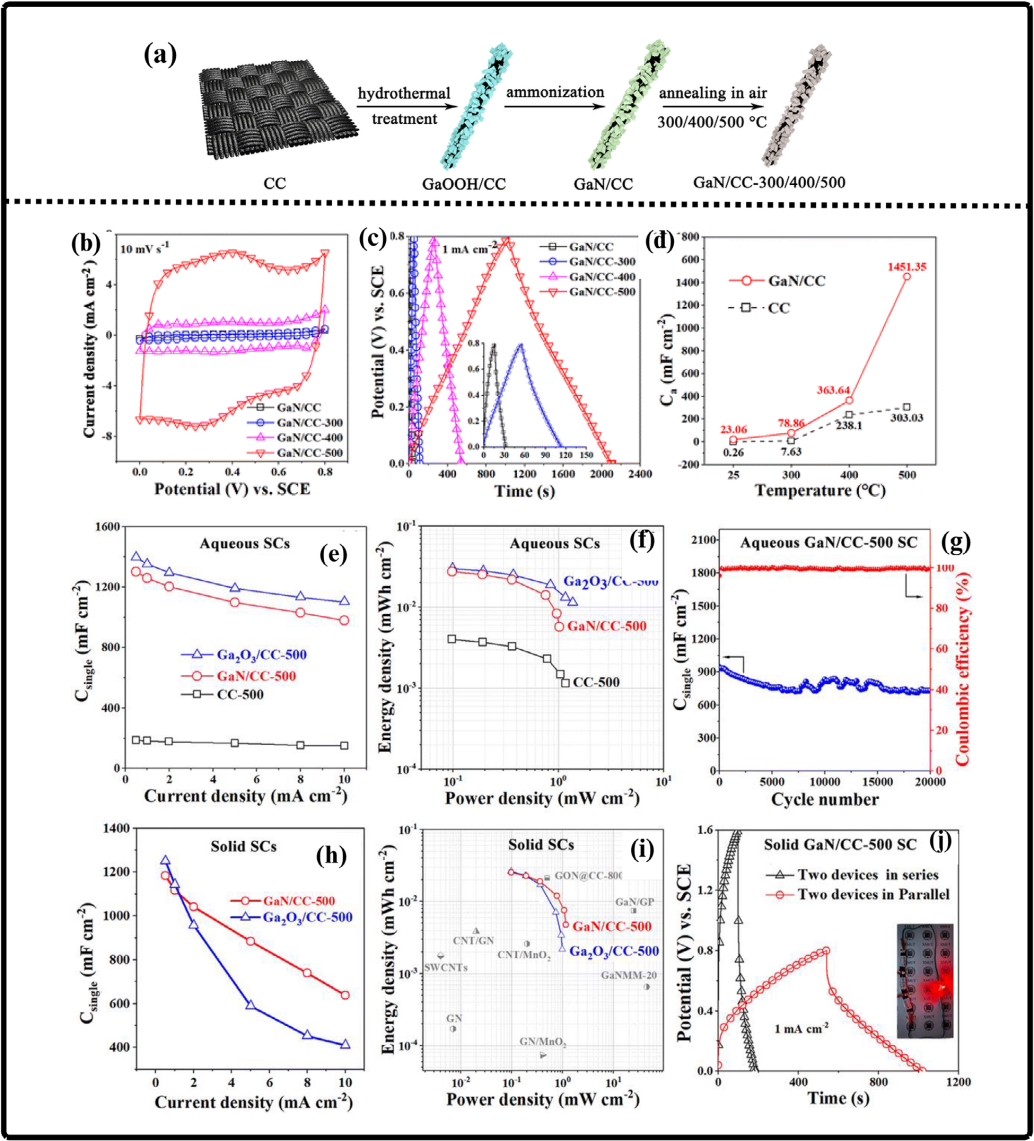
(a) Illustration of the fabrication for GaN/CC electrodes, (b) CV curves for GaN/CC electrodes, (c) GCD curves for GaN/CC electrodes, (d) Areal capacitance vs. temperature of the CC and GaN/CC electrodes computed from GCD data, Symmetric Supercapacitor device in 1 M H_2_SO_4_ aqueous electrolyte: (e) Capacitance for different SC at 500°C annealing temperature, (f) Ragone plots, (g) Cyclic Stability and Coulombic efficiency of GaN/CC‐500 Symmetric SC, Symmetric Supercapacitor device PVA‐H_2_SO_4_ solid gel electrolyte: (h) Capacitance for different SC at 500°C annealing temperature, (i) Ragone plots, and (f) GCD curves for two GaN/CC‐500 SC devices in series and in parallel, inset shows a red LED powered by three GaN/CC‐500 SCs in series combination (Reproduced with permission from ref. [214], Copyright Royal Society of Chemistry, 2022).

Transition metal oxide (TMOs) character in GaN‐based supercapacitors is essential to overcoming the intrinsic capacitance boundaries of metal nitrides (MN), as the oxide phase offers important active sites for electrochemical faradic redox reactions, and the nitride core ensures rapid electron transport [216]. This interactive method was confirmed by Hu et al., who fabricated a hybrid GaN/Ga_2_O_3_ microrod on carbon cloth (CC) via a hydrothermal process [214] combined with a high temperature (300°C–500°C) nitridation, followed by a critical air‐annealing stage to generate surface oxidation, as illustrated in Figure 14a. By increasing the post‐annealing temperature to 500°C, both specific capacitance and electron density of the composite electrode were improved. The combination of α‐Ga_2_O_3_ with GaN is the pivotal factor in exposing the high energy storage ability of the GaN‐based supercapacitor, efficiently transitioning the device from a low‐capacitance double‐layer (EDLC) system to a high‐performance pseudocapacitive SCs electrode. While the wurtzite GaN core roles predominantly as a conductive electron trench, the α‐Ga_2_O_3_ shell developed during air‐annealing assists as the fundamental electrochemical engine offering abundant active sites for faradaic redox reactions [217].

To check the impact of post‐annealing temperature on electrochemical performance, cyclic voltammetry (CV) and galvanostatic charge/discharge (GCD) analyses were conducted on GaN/CC electrodes in a three‐electrode system using a 1 M H_2_SO_4_ electrolyte. For comparison, they used annealed carbon cloth (CC) substrates, which showed that both the bare CC and composite electrodes have quasi‐rectangular CV curves and symmetric triangular GCD curves, demonstrating ideal capacitive behavior as shown in Figure 14b,c. A definite positive connection was observed between the annealing temperature and the specific areal capacitance, as they stated, the areal capacitance of the GaN/CC electrode at increasing the post‐annealing temperature can be significantly optimized, as illustrated in Figure 14d.

In the case of a real‐time device, they assembled a GaN/CC‐500 symmetric supercapacitor (SC), because GaN/CC‐500 has the highest specific capacitance and electron density as an electrode, which showed great potential in both 1 M H_2_SO_4_ aqueous electrolyte and a PVA‐H_2_SO_4_ solid gel electrolyte. In comparison to this SC, Hu and his team also assembled the symmetric Ga_2_O_3_/CC‐500 SC to test under the same conditions. For both GaN/CC‐500 and Ga_2_O_3_/CC‐500, all of the GCD curves at minimal current densities demonstrated ideal symmetric triangular shapes in aqueous and solid electrolytes. Charge discharge curves revealed that at higher current densities, aqueous GaN/CC SC has a higher IR drop and lower capacitance compared to the aqueous Ga_2_O_3_/CC‐500 SC, as illustrated by rate performance in Figure 14e. The capacitance of the solid GaN/CC‐500 SC fell from 1183.35 to 638.77 mF cm^−2^, with 53.98 % of the specific capacitance retention, while the capacitance retention of the solid Ga_2_O_3_/CC‐500 SC is only 32.8 % under the same conditions. In contrast, solid GaN/CC SC has a lower IR drop and higher capacitance than the solid Ga_2_O_3_/CC‐500 SC at a high current density, as confirmed in Figure 14h. Results suggest that when the current density was increased to 10 mA cm^−2^, the capacitance of the aqueous GaN/CC‐500 SC declined from 1301.2 to 978.95 mF cm^−2^. The aqueous GaN/CC‐500 SC showed a capacitive retention of 77.27 % after 20 000 charge–discharge cycles under 10 mA cm^−2^, in comparison with aqueous Ga_2_O_3_/CC‐500 SC, which retained a capacitance of 76.5 %. Ragone plots in Figure 14f,i follow the same trend. GaN/CC‐500 SC in solid electrolyte is more prominent than GaN/CC‐500 SC, which proves GaN/CC‐500 is advantageous over Ga_2_O_3_/CC‐500 SC as an electrode for solid‐state Supercapacitors. The microrods with the GaN/Ga_2_O_3_ hybrid structure demonstrated remarkable potential as an assembled symmetric solid SC with a capacitance of 169.2 F g^−1^ at 0.07 A g^−1^, transporting an energy density of 0.58 Wh kg^−1^, and owning 154 W kg^−1^ power density. Figure 14j shows that the indication of real‐time power delivered by three solid GaN/CC‐500 SCs linked in series and charged to 2.4 V can light up an industrial red LED for ∼690 s, indicating its importance as a capable energy storage material. The optimized electrochemical performance of GaN/CC‐500 is attributed to the hybrid construction of GaN/α‐Ga_2_O_3_, with the latter permitting a rapid transport of electrons within the microrods and delivering the active sites for redox reactions with the electrolyte due to the metal oxide.

The binary transition metal oxides (NiCoO_2_) in GaN‐based supercapacitors primarily serve as a high‐capacity pseudocapacitive material, providing abundant active sites and variable valence states for electrochemical reactions. However, to overcome the inherent issue of NCO aggregation and ensure stability at high temperatures, Lv et al. employed a strategy where p‐type NiCoO_2_ (NCO) nanosheets are grown in situ on an electrochemically etched n‐type porous GaN membrane [26]. Nickel cobalt oxide (NiCoO_2_), which was suitable for direct attachment onto porous GaN crystal to set up GaN‐based integrated electrode material. The porous GaN behaved as a conductive substrate favourable to the equal distribution of working electrode material, which prevented the issue of severe NiCoO_2_ clusters [218].

In this research, a porous GaN/NiCoO_2_ heterostructure electrode was fabricated. Subsequently, the device was assembled based on the porous heterostructure electrode with ionic liquids made by GaN and exhibits outstanding features in a high‐temperature environment. It is important to note that the new structural design, which is characterized by good bonding between the porous substrate (GaN) and NCO NSs, is favorable in the achievement of synergy. The pore channels are interconnected not only to minimize diffusion paths of the ions and electrons, but also to buffer the volume expansion during the intense shocks of the current to enhance the stability of the device. More importantly, the heterostructure of GaN/NCO leads to an increase in the specific surface area and active sites, which is important in intensifying the adsorption of ILs to boost energy capture. The NiCoO_2_ nanosheets have high active sites, which are highly electrochemically active. Conversely, the option of nonflammable, thermally stable, and diverse‐voltage ionic liquids as electrolytes [219], which is another vital direction of raising the stored energy of the GaN‐based devices without compromising the power density in the high‐temperature set‐ups, is preferable.

**FIGURE 15:**
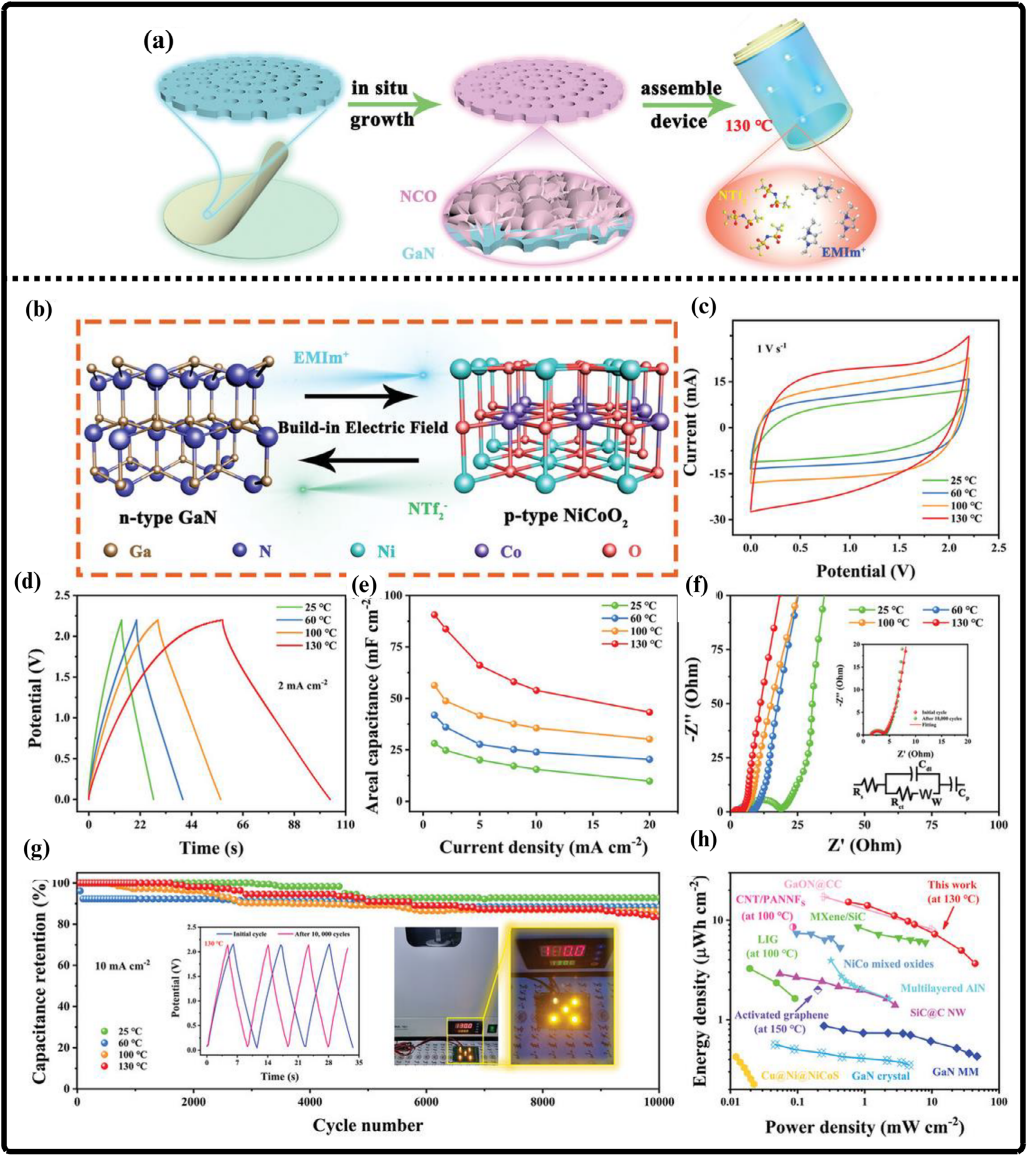
(a) Schematic synthesis process of the GaN/NiCoO_2_ heterostructures, (b) Schematic of n‐type GaN and p‐type NiCoO_2_, (c) CV curves, (d) GCD, (e) Areal capacitance at different current densities with varying temperatures, (f) EIS curves of GaN/NiCoO_2_ heterostructures with circuit fitting model, (g) Cyclic stability, (h) Ragone plots comparison with reported data (Reproduced from ref. [26] Copyright 2023, John Wiley & Sons).

The heterostructure was prepared through a systematic approach using a series of varying ratios of GaN based on a heterostructure, as demonstrated in Figure 15a, which indicates a connected p‐type NCO nanosheet group to grow on an n‐type porous GaN substrate. Concisely, porous GaN is etched out of sapphire (Al_2_O_3_) substrates. The Ni‐Co forerunners are sequentially prepared on the surface of the porous GaN through a single‐step hydrothermal process in a homogeneous manner. The heterostructure made of GaN generates an inherent electric field across the electrode material as represented in Figure 15b. This internal field plays a fundamental role in facilitating electrolyte ion adsorption, thereby improving electrochemical kinetics. To evaluate the realistic applicability of this architecture under harsh conditions, they assembled SCs developing GaN/NCO‐2 electrodes and ionic liquid (ILs) electrolytes. The performance of these devices was afterward calculated over a range of increasing temperatures to conclude their thermal stability and energy storage ability. In Figure 15c, the quasi‐rectangular CV curves exhibit characteristics standard of electric double layer capacitors (EDLCs), notably the absence of redox peaks (Pseudocapacitance), which confirms that the device maintains stable capacitive behavior across the temperature range of 25–130°C. The GaN/NiCoO_2_ device is characterized by a high stability level, as the CV curves do not lose their shape even when scan rates increase 800 times at 130°C. The respective GCD plots in Figure 15d are quasi‐triangular in shape, which is associated with CV curves, which also confirms ideal capacitive behavior (EDLC). Figure 15e indicates the computed areal‐specific capacitance; at 1 mA cm^−2^ and 130°C, the value reaches 90.6 mF cm^−2,^ which was considerably higher than the capacitance of 28.4 mF cm^−2^ recorded at 25°C. This improvement is contributed to by the thermal efficiency of the electrolyte, in this case, the decreased viscosity and increased conductivity of the ILs [132], and the high thermal stability of GaN [220] at elevated temperatures. Moreover, this mechanism is also supported by EIS measurements. Figure 15f indicates that the intercept of the high frequency changes to lower values as temperature increases, which means that the internal resistance (R_s_) of the active material and electrolyte is lower. At the same time, the reduction of the interfacial resistance indicates an increase in the speed of charge transfer. This ascertains that the ionic conductivity of the heterostructure‐based device based on GaN/NCO heterostructure‐based SC is significantly enhanced at 130°C. The plots in the low‐frequency region have vertical lines, which show great capacitive properties. Moreover, the lower Warburg impedance at high temperatures indicates that diffusion of ions by the electrode bulk is extremely facilitated. It is noteworthy that the device is incredibly resilient, maintaining 83.6 % of its original capacitance following 10 000 cycles at 130°C, as can be observed in Figure 15g. Highly stressed with high‐current heterostructure may occur multiple times with high temperature due to the strength of the structural material of the GaN/NCO. More stability is also observed in the insets of Figure 15g,f, the GCD curves and EIS spectra at the beginning of the cycle and the postcycling cycle show little variation. To demonstrate the possible application, the porous GaN‐based device could activate five light‐emitting diodes (LEDs) in a cross pattern (Figure 15g), which verified its possible application in a high‐temperature environment. Lastly, energy and power output are the most important measures to use in a modern‐day storage system. Figure 15h also showed that the SC device based on the heterostructure of GaN/NCO provides a peak energy density of 15.3 µWh cm^−2^ and a peak power density of 44 mW cm^−2^. This means that there is a high increase in energy capture without compromising on power density, even in high temperatures.

**FIGURE 16:**
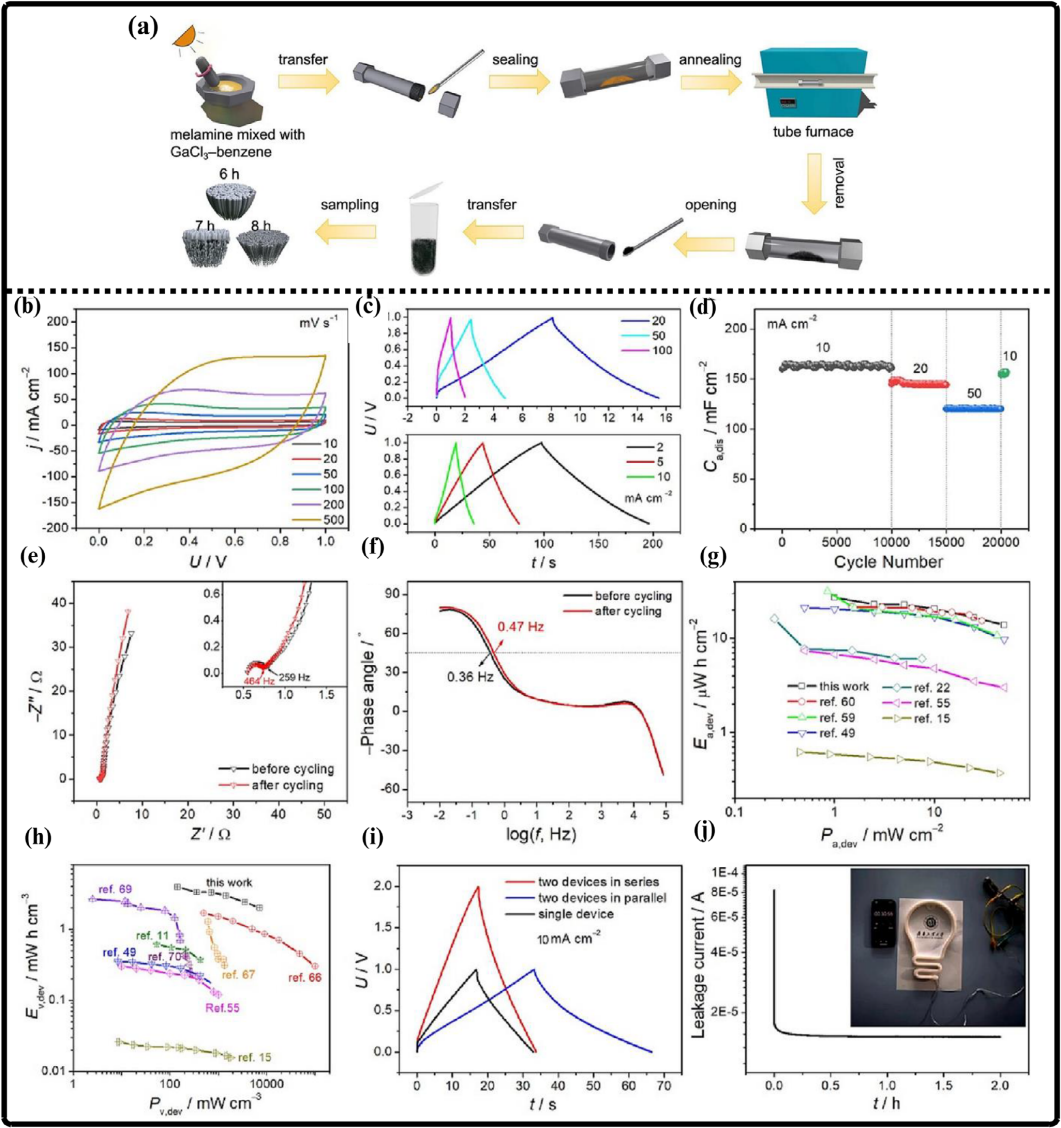
Schematic of Synthesis process of GaN/nitrogen‐doped carbon (GaN/N‐C), (b) CVs of GaN/N‐C symmetric Supercapacitor device, (c) GCD curves, (d) cyclic stability, (e) Nyquist plots, (f) Bode plots, (g,h) Ragone plots, (i) GCD curves of the devices in series and parallel, (i) leakage current plot with a picture of five devices in series lightening a neon LED taken at 30.56 s (Reproduced with permission from ref. [194] Copyright 2023 Elsevier).

Due to the benefits of multi‐scale architecture, Li and his associates devised a plan to synthesize hierarchically organized porous GaN microparticles/nitrogen‐doped carbon [194]. This was done through an altered carbothermal reduction in a closed system, as in Figure 16a [221], which uses the established thermal instability of GaN. It was done through a modified carbothermal reduction in an enclosed system (Figure 16a) [166], which takes advantage of the thermal instability of GaN. They showed that electrochemical performance can be enhanced by changing the annealing time, which is the direct determinant of important properties, including surface area, morphology, density of nitrogen defects, pore structure, and the weight ratio of GaN to carbon.

The electrochemical properties of the S‐C‐t and S‐O‐t series were initially evaluated in a three‐electrode system using active material mass loadings of 3–9 mg cm^−2^, where t denotes the annealing time. In terms of performance, the integrated CV area increased as t bumps up from 6 to 8, before decreasing at t = 9. Since a larger CV area at a fixed scan rate correlates with higher specific capacitance, S‐C‐8 exhibited exceptional performance. In the three‐electrode mode, GCD was tested at a current density of 1 mA cm^−2^; the capacitances were 15, 129, 275, 448, and 389 mF cm^−2^ for S‐O‐8, S‐C‐6, S‐C‐7, S‐C‐8, and S‐C‐9, respectively. As C_a_ is approximately 30 times that of S‐O‐8, underscoring the significant advantages of the hierarchically structured porous GaN/carbon architecture over the aggregated GaN/carbon counterpart.

In order to determine the usability of S‐C‐8 in practice, symmetric supercapacitor devices (S‐C‐8||S‐C‐8) were prepared with a large mass loading of 7.6 mg cm^−2^. The Cyclic Voltammetry (CV) curves have a quasi‐rectangular shape at scan rates up to 500 mV s^−1^, as in Figure 16b, which is characteristic of charge storage via EDLC. Similarly, the Galvanostatic Charge–Discharge (GCD) curves maintain a very symmetric triangle form over the current densities of 1–50 mA cm^−2^, as indicated in Figure 16c, and indicate a particularly good rate capability. That symmetric device presents an areal discharge capacitance of 100 at 100 mA cm^−2^, a capacitance retention of 51% compared to the capacitance retention present at 2 mA cm^−2^. Moreover, the device has impressive durability after 20 500 current cycles at 10–50 and again at 10 mA cm^−2^ current densities. The capacitance of the device is 157 mF cm^−2,^ which represents 98.1% retention of capacitance compared to the first cycle, as given in Figure 16d.

The Electrochemical Impedance Spectroscopy (EIS) was used to validate the stability of the device. Figure 16e Nyquist plots show that the reduction in R_ct_ (0.05 Ω) and the vertical low‐frequency tail increase after cycling, indicating an activation process that increases conductivity. Figure 16f is an analysis of the Bode phase angle that shows almost perfect capacitive behavior with a shift in the values to 78° to −80° after cycling. This closure to the perfect 90° limit [204], together with the typical frequency analysis at −45°, attests to the effective ion transfer as well as the quick reaction to frequencies in the device. The device has good energy‐power characteristics as shown in the Ragone plots of Figure 16g,h. It gives 27.2 µWh cm^−2^ at a power density of 1 mW cm^−2^. Moreover, the energy density is strong at 13.9 µWh cm^−2^ even at an extreme power production of 50 mW cm^−2^. Such findings are contrasted with the past literature of the Supercapacitor based on GaN in Figure 16g,h.

The series and parallel connections in Figure 16j were used to assess the applicability of the results to the real world, where doubling of voltages and capacitance was expected. The high‐power performance of the device was graphically evidenced: a three‐unit series of connections could light up a panel of 23 blue LEDs for > 1 min, and a five‐unit series of connections could light up a high‐demand neon LED for > 30 s. More importantly, the device is highly efficient in charge retention as shown by a low leakage current that levels at 15–16 µA following a decay period of 3.54 min. The low leakage current allows the S‐C‐8 architecture to be an excellent target in self‐powered systems powered by intermittent energy harvesters.

A breakthrough has been the mesoporous structure of gallium nitride (GaN) membranes that have turned GaN into a highly efficient electrode of a supercapacitor and not just a traditional semiconductor. Wang et al. accomplished this by producing a network of mesopores in the form of dendrites between 20 and 60 nm using a two‐step electrochemical etching that was carefully controlled [204]. This change is more than a simple change. They manage to surmount the intrinsic drawbacks of bulk GaN, such as low surface area and poor ion accessibility, by restructuring the inside of the material. Simultaneously, the procedure does not lose the good electrical characteristics of GaN.

**FIGURE 17:**
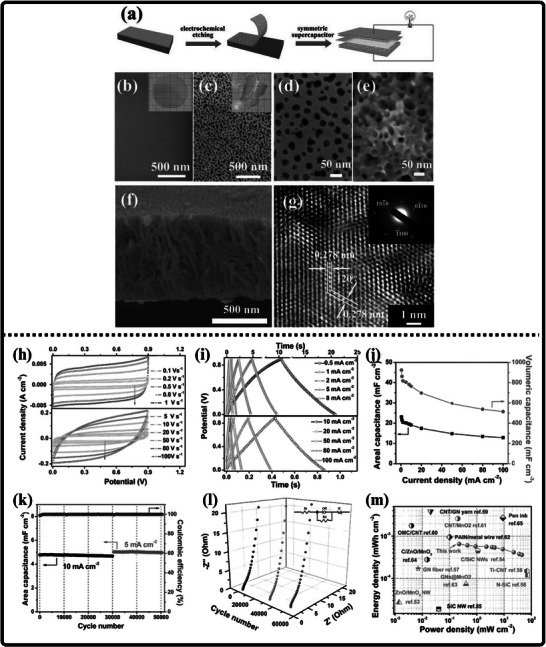
(a) Schematic showing how the supercapacitor is made. (b,c) show SEM images for GaNMM‐20 and GaNAG. SEM images of GaNMM‐20 are shown from the front (d), the opposite side (e), and as a cross section (f). In (g), HR‐TEM image of GaNMM‐20, with the inset highlighting the SAED pattern, for a two‐electrode SC device, (h) CV profiles, (i) GCD curves, (j) Capacitance relative to current density, (k) Capacitance relative to cycle number, (l) EIS curves, (m) Comparison with the literature. (Reproduced with permission from the reference [204] Copyright 2016 John Wiley & Sons).

The process begins with n‐type GaN grown by MOCVD. Controlled anodic etching is done in an acidic solution, where nucleation starts, and then these nucleation sites are further grown into a consistent system of branched pores. The whole process of fabrication, membrane lift off, till the assembly of the device can be scaled as shown in Figure 17a. The parameters of all the processes are optimized accurately to make sure that the single‐crystalline structure of hexagonal wurtzite is maintained. TEM images with high resolution have lattice fringes that are indisputable evidence of the presence of GaN (002) planes, and the lattice fringes of the selected‐area electron diffraction pattern are of single‐spot patterns that manifest the absence of grain boundaries that might hinder the transportation of electrons. The resulting GaNMM‐20 membrane with 20 nm pores has an inherent specific surface area of 23.2 m^−2^ g^−1^, which is about 20 times higher than that of bulk GaN, as confirmed by SEM and HR‐TEM, as illustrated in Figure 17b–g. Moreover, it is highly electrically conductive (44.19 S cm^−1^) as a result of the continuous crystal routes. This mesoporous structure provides three key advantages: First, it increases the electrode‐electrolyte interface area, enabling greater charge accumulation via electric double‐layer capacitance (EDLC). Second, dendritic pores establish direct, low‐tortuosity pathways that increase the diffusion of ions and permit a large decrease in the Warburg impedance in EIS. Third, single‐crystalline walls maintain their structural stability in cycling and prevent the wall from falling or deteriorating the structure.

The electrochemical performance of the membrane was determined by the intrinsic capacity of the material in a classical three‐electrode configuration using 1 M H_2_SO_4_. The mesoporous GaNMM‐20 electrode, with a scan rate of 0.01 V s^−1^ and an areal capacitance of 23.67 mF cm^2^, was attributed to the effective formation of the Helmholtz double layer on the increased surface area. Even with a 100 V s^−1^ scan rate, the capacitance had decreased to 4.35 mF cm^−2^, and the retention was large at 84, the highest ever seen with the traditional carbon‐based electrodes. This is explained by the optimum pore size at 20 nm, small to provide high surface area, big enough to provide easy ion movement, and the crystalline lattice of the lattice permits speedy electron movement. A current density of 10 mA cm^2^ on the electrode capacitance retained over 77 % of its original capacitance after 20 000 cycles. Its single crystal form is very mechanically strong, and mesopores have the capability of withstanding repeated ion implantations without any structural losses. The voltammetry plot curves are nearly rectangular at large scan rates, and hence the capacitive behavior is ideal with minimal polarization, and the shape of charge–discharge profiles is symmetric, triangular, and therefore indicates a low internal resistance and high efficiency.

Electrodes made of GaNMM show high functionality when connected together in a two‐electrode symmetric supercapacitor. The device provides an aerial capacitance of 12.1 mF cm^−2^ at a scan rate of 10 V s^−1^ within a potential range of 0.8 V. Due to the very low equivalent series resistance (1.2 Ω, measured by EIS), the power density is more than 100 kW L^−1^ while long‐term stable operation is maintained. Ragone plot has enumerated this device as one of the most effective performing devices because of the combination of high electrical conductivity of GaNMM, which enables quick charge discharging kinetics, and the mesoporous structure, which enables deep penetration of electrolyte, resulting in high performance. Figure 17h–m provides the entire electrochemical image of the GaNMM‐20 membrane.

The team combines a dendritic pore structure and a single‐crystal semiconductor to make the most of a synergistic effect that is impossible in polycrystalline or amorphous semiconductors. The mesopores serve as ion transport channels to diffuse H_2_SO_4,_ and crystalline GaN walls are efficient electrophilic conduction channels; together, these provide a fast charge storage mechanism. Not only is this work establishing a new standard of GaN‐based supercapacitors, but it also offers a model for improving semiconductor electrodes by using carefully designed mesostructured engineering. The consequences are also widespread, including not only flexible electronics and electric vehicles but also grid‐scale energy storage applications.

**FIGURE 18:**
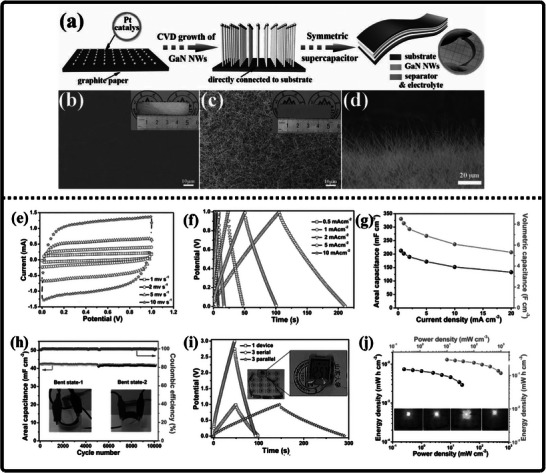
(a) Fabrication and assembly steps for the GaN/GP‐based flexible supercapacitor, followed by SEM images: (b) shows the GP before GaN nanowire growth, and (c) shows it after nanowires have formed. In (d), see a high‐magnification SEM image of GaN/GP‐2. For the GaN/GP‐2 electrode‐based supercapacitor device, electrochemical results include (e) CV curves at various scan rates, (f) GCD at different current densities, (g) volumetric and areal capacitances of a single electrode compared across current densities, and (l) cycling performance under different bending conditions at 5 mA cm^−2^. In (h), GCD curves compare different supercapacitor device configurations at 1 mA cm^−2^, with the inset showing optical images of three supercapacitors connected in series to power an electronic watch. Finally, (i) presents Ragone plots for the GaN/GP‐2 supercapacitor device powering multiple LEDs. (Reproduced with permission from ref. [212] Copyright 2016 John Wiley & Sons).

The use of gallium nitride (GaN) in advanced energy storage systems, especially supercapacitors, is being changed by gallium‐based composites [222]. GaN itself has the weakness of moderate electrical conductivity and mechanical rigidity. Its performance, though, can be greatly improved when used together with appropriate complementary materials. Shouzhi et al. have contributed significantly to the development of a flexible supercapacitor electrode by incorporating GaN nanowires and graphite paper [212]. The principle is quite simple but efficient: GaN, which is a material possessing high charge efficiency, is wrapped around a very conductive and adaptable substrate of carbon. This conglomeration offers good electrochemical functionality. To produce these electrodes, the authors employed the catalyst‐assisted chemical vapor deposition (CVD) in growing single‐crystalline GaN nanowires on graphite paper, which is depicted in the synthesis process in Figure 18a. This is essential since it ensures that the nanowires adhere well to the substrate to facilitate proper movement of the electrons and also increase the area of effective storage of the charge, that is, the nanowires possess a special morphology as provided in Figure 18b–d. They used a three‐electrode system in their electrochemical experiments; a counter electrode was platinum foil, a Hg/Hg_2_SO_4_ reference electrode, and a 1 M H_2_SO_4_ electrolyte. The GaN/graphite paper electrode could achieve a specific capacitance of 237 mF cm^−2^ under a current density of 0.1 mA cm^−2^. The performance of this high‐performance has been a result of the influence of the synergy of the pseudocapacitive characteristics of the GaN and the high conductivity of the graphite paper. After 10 000 cycles of charge–discharge, the capacitance of the electrode was 98% of its initial value, which shows that the nanowires were highly structurally stable.

The scientists wanted to test the practical viability of the fabricated flexible symmetric supercapacitors; thus, the team placed two electrodes of GaN/graphite paper on the solid‐state Hg_2_SO_4_/polyvinyl alcohol (PVA) electrolyte. This design in solid‐state enhances the safety, flexibility, and mechanical strength of the device in general. The energy density of 0.30 mWh cm^−3^ and power density 1000 mW cm^−3^ were outstanding, as well as the areal (53.2 mF cm^−2^) and volumetric (2.1 F cm^−3^) capacitance at 0.5 mA cm^−2^. The proper setup of the SC device, along with the electrochemical results, is given in Figure 18e–j. The electrodes were properly characterized during the process of fabrication, SEM imaging of the nanowires, and electrochemical testing in three and two‐electrode arrangements. The findings assured rapid charge storage dynamics and low internal resistance. GaN/graphite paper composites are one of the types of gallium‐carbon hybrid materials that have been studied as promising in terms of their use in flexible energy storage. This not only improves the electrochemical performance of GaN but also gives it the mechanical flexibility and durability needed in wearable and portable electronic technologies. The results show that with smart composite design, GaN can be stretched far beyond its old use as a component in optoelectronic equipment and can represent a formidable competitor to next‐generation energy storage systems.

**FIGURE 19:**
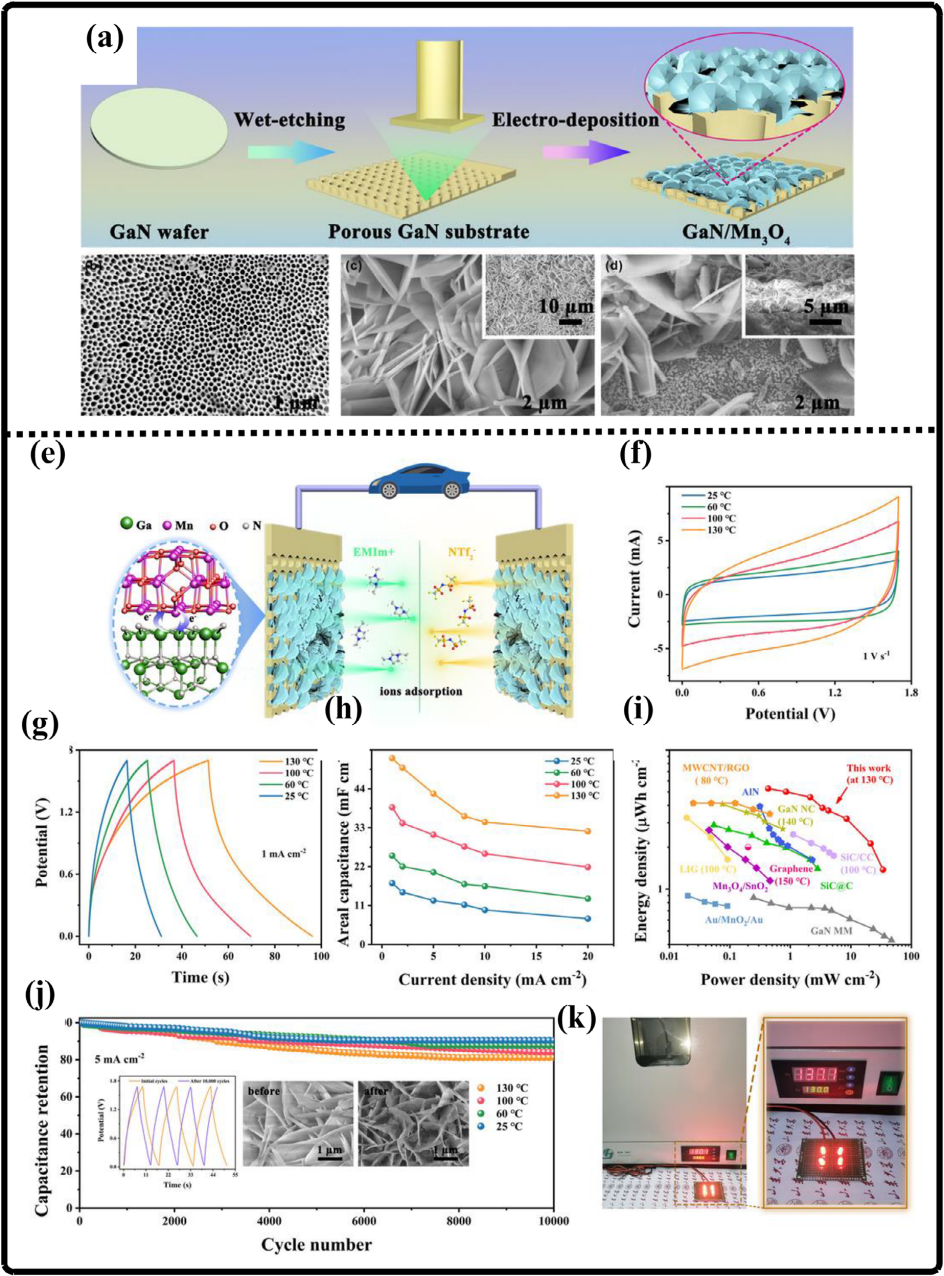
(a) Shows how GaN/Mn_3_O_4_ was prepared, (b) SEM image of the porous gallium nitride single crystal, (c) SEM of the GaN/Mn_3_O_4_ composite, (d) Cross sectional view, For the GaN‐based supercapacitor: (e) Schematic illustration of charge storage behavior in the GaN/Mn_3_O_4_ supercapacitor, (f) CV curves, (g) GCD profiles, (h) capacitance as a function of current density, (i) Ragone plot comparing with other reports in the two‐electrode system, (j) capacitance retention after multiple GCD cycles, (k) real‐time photos of an LED powered by the GaN/Mn_3_O_4_ supercapacitor (Reproduced with permission from ref. [184] Copyright 2025 John Wiley & Sons).

The development of metal oxide composites opens up some possibilities to improve the electrochemical performance of the gallium nitrate (GaN)‐based supercapacitors. GaN can be a desirable conductive base due to its chemical and mechanical stability, broad bandgap, and broad bandgap [137, 223, 224]. Nevertheless, the storage charge is limited. Through the integration of pseudocapacitive metal oxides on GaN, scientists can take advantage of the stability and conductivity of GaN and the high theoretical capacitance of metal oxides and rich redox properties. This is especially important in the case of supercapacitors that are needed to work reliably at high temperatures. The self‐supported GaN/Mn_3_O_4_ composite electrode proposed by Lv et al. [184], built specifically for high‐temperature energy storage as designed in Figure 19a, namely, tailored to the requirements of the high‐temperature energy storage application (see Figure 7a), is a representative example. They start with a porous single‐crystal substrate of GaN, and the chemical method known as wet etching is used to form a porous network that is highly porous and three‐dimensional network. This porous structure not only gives enhanced morphology but also enhances the accessible surface area available for the deposition of a material and electrochemical reactions. Moreover, the pores are networked in such a way that the electrolytes can reach deeper regions of the electrode, thereby enhancing the ion movement. The complex geometry of such morphology is depicted in the SEM image shown in Figure 19b–d.

After acquiring the porous scaffold of GaN, Mn‐based precursors of the oxide are sourced on the scaffold by using in situ electrochemical deposition. The process allows a close control of the coating process, such that the films of Mn(OH)_2_ (or analogs thereof) are conformal and uniform, over the complicated geometry of the GaN structure. The electrochemical conditions favor the selective growth of these precursors on the GaN surface, which forms close contact between interfaces that is critical to high‐rate charge transfer. Annealing subsequently transforms manganese hydroxide to electrochemically active Mn_3_O_4_. Annealing parameters are greatly fine‐tuned to obtain Mn_3_O_4_ in the form of nanosheets, which are highly attached to the underlying porous GaN. The interface is critical in ensuring that interfacial resistance is kept to a minimum, as well as the levels of mechanical stability within the structure. The obtained product, as illustrated by the SEM images, is an independent electrode made out of Mn_3_O_4_ nanosheets tightly bound to the porous GaN backbone to create a layered and mechanically strong design. The crystal structures of the major constituents, namely, porous GaN, the composite, and pure Mn_3_O_4_, are further confirmed by the XRD patterns.

The outstanding performance of this design is illustrated with the help of an electrochemical assessment in a three‐electrode set‐up. The combination of the two materials has a high specific capacitance of 448.6 mF cm^−2^ at a 1 mA cm^−2^ current density. It still has a current density of 223.1 mF cm^−2^ even at 50 mA cm^−2^. Stability in cycling is also impressive; the electrode maintains 84 percent of its original capacitance after 10 000 charge–discharge cycles at 8 mA cm^−2^. The GaN/Mn_3_O_4_ mixture does not deteriorate in a practical two‐electrode system using an ionic liquid electrolyte at 130°C, which yields 34 mW cm^−2^ power density and 5.31 µWh cm^−2^, and keeps 81.3 % of its capacitance after 10 000 cycles at 5 mA cm^−2^, as shown in Figure 19j. Redox‐active Mn_3_O_4_ nanosheets increase the charge storage capacity, kinetics of electron/ion transport, and thermal stability through the synergistic effect between porous and conductive GaN and Mn_3_O_4_. This paper presents an encouraging path towards high‐energy storage systems that can be sustained under conditions of high temperatures.

Carbon‐based materials have been proven as the key to the enhanced performance of the gallium nitride (GaN)‐based supercapacitors, being significant to their overall electrochemical properties [225]. Being a wide‐bandgap semiconductor, GaN is restricted by its moderate electrical conductivity, as well as by a limited number of electrochemically active sites, which limit its usability in energy storage applications. To address these drawbacks, scientists have been exploring more and more carbon additives, which not only help to cancel the inherent disadvantages of GaN but also provide the synergistic effects that promote the overall work of the device. One of the carbon materials that has excelled in terms of electrical conductivity and structure is reduced graphene oxide (rGO). Nongthombam et al. use rGO in the form of a conductive network, as well as a high‐surface‐area scaffold, to successfully alter the behavior and functional integration of GaN nanoparticles [19]. The rGO has a three‐dimensional structure, which allows rapid transport of electrons and thus lowers the charge transfer resistance, which is the traditional limitation of the electrochemical activity of GaN. The efficacy of this hybrid structure is quantitative, and electrochemical impedance spectroscopy (EIS) demonstrates that the composite has an unusually low charge transfer resistance of just 2.36 Ω, indicating the success of this hybrid structure.

Surface area of rGO extends to 2630 m^2^ g^−1^, which not only enhances its properties as an electrical conductor but also reduces the aggregation of the GaN nanoparticles, thus solving a major problem that reduces the active surface area and the overall performance, but also enables more electroactive sites to be involved in the charge storage process. The scanning electron microscopy (SEM) analysis shows that the thin nanoparticles of GaN are evenly spread and well attached to the rGO sheets. This structure facilitates high ion diffusion because it offers clear spaces to allow the diffusion of electrolyte ions to react with the active sites, which is vital in rapid charge and discharge reactions. Moreover, the carbon structure improves the mechanical strength of the composite to maintain structural integrity in repeated electrochemical cycling. To illustrate the high resistance to degradation of the rGO scaffold, the most successful one, GaN5%@rGO‐GaN, can withstand 75 % of its original capacitance after 950 charge–discharge cycles, and this indicates the physical and electrochemical stability provided by the rGO scaffold.

**FIGURE 20:**
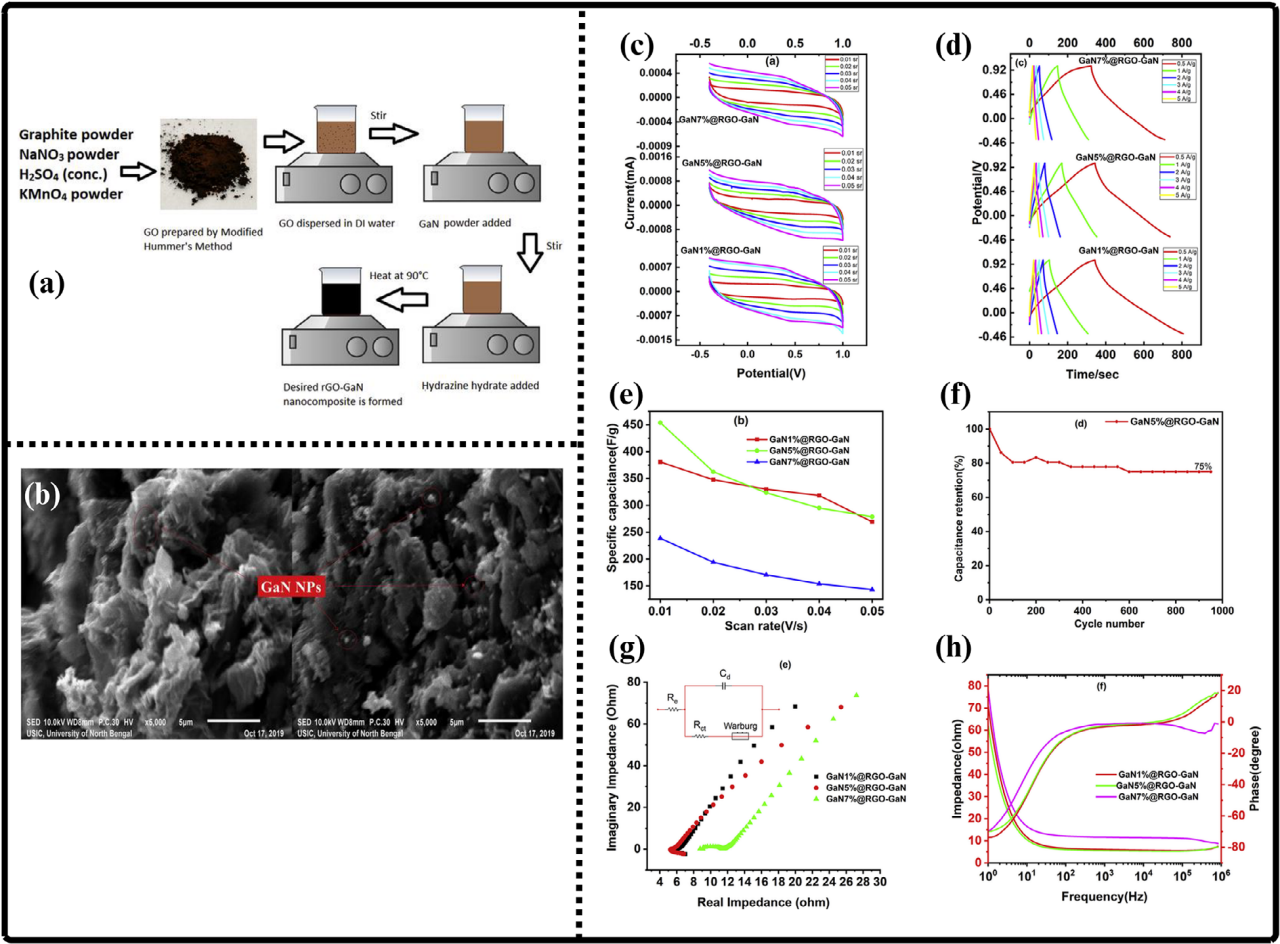
(a) Preparation GaN/rGO composite (b) Scanning Electron Microscope images of GaN/rGO (c) Cyclic Voltammetry, (d) Charge Discharge data, (e) Capacitance, (f) Cyclic stability of GaN5%@rGO‐GaN for 950 cycles, (g) Impedance Spectra (Nyquist), and (h) Bode plot of composites (Reproduced with permission from ref. [19] Copyright 2020 Elsevier).

The formation of these rGO/GaN nanocomposites is easy and reproducible, and it requires only a single‐step reduction process. This synthesis starts with the Method of Modified Hummer, a long‐standing procedure of transforming graphite into graphene oxide (GO) by oxidation using NaNO_3_, concentrated H_2_SO_4_, and KMnO_4_. The resulting GO is extremely functionalized with oxygen‐related groups that not only increase its hydrophilicity and dispersibility in water but also act as the location sites of GaN nanoparticles. GO is then combined with different percentages of GaN powder (between 1 % to 20 %) and then chemically reduced using hydrazine hydrate at 80°C. This step of reduction also transforms GO to rGO and immobilizes the GaN nanoparticles in the conductive matrix, thus providing close contact between interfaces and ensuring effective pathways of charge transfer. It is also a simple and effective process, as demonstrated in Figure 20a, and it can be used in large‐scale production. The successful integration of GaN into the rGO matrix and the quality of the complex are confirmed by the post‐synthesis characterization of SEM, X‐ray diffraction (XRD), Fourier‐transform infrared spectroscopy (FTIR), X‐ray photoelectron spectroscopy (XPS), and UV–vis spectroscopy, while morphology was confirmed by SEM as shown in Figure 20b. Such analyses ensure maintenance of the intended crystalline structure, functional group presence, and good interfacial interactions between the two components, which are important in attaining the best electrochemical performance.

Electrochemical characteristics of rGO/GaN nanocomposites were determined in a standard three‐electrode setup using a 1 M H_2_SO_4_ electrolyte. Cyclic voltammetry (CV) results show that the GaN5% rGO‐GaN sample has a high specific capacitance of about 454 F g^−1^ at a scan rate of 10 mV s^−1^, as given in Figure 20e. Moreover, this sample shows lower charge transfer resistance and series resistance, as confirmed from EIS analysis, as illustrated in Figure 20g. This is a good compromise between the amount of GaN and the conductive matrix, which proves the concept of compositional tuning in the optimal performance of devices. Combined effects of rGO and GaN result in GaN‐rGO forming a high electric double‐layer capacitance (EDLC) because, owing to its accessible surface area, it has high capacitance caused by a basic susceptibility to surface redox reactions, whereas GaN contributes pseudocapacitance, which is the capacitance generated by surface redox reactions. This compensatory process is also justified by galvanostatic charge–discharge (GCD) tests, which report given capacitance values of between 116.8 and 146.1 F g^−1^ with a current density of 1 A g^−1^. The symmetrical GCD curves are evidence of effective and reversible charge storage with a small resistive loss, which proves high energy efficiency.

EIS analysis also confirms this data, with the Nyquist plots nearly vertical at low frequencies and the semicircles within the area of the semicircles in the high frequencies. These properties consist of low charge transfer resistance and near ideal capacitive characteristics, which demonstrate that the composite can efficiently carry electrons and ions. The enhancement of electrochemical kinetics and stability herein was also directly attributed to the close interfacial interaction and physical contact between rGO and GaN.

The use of rGO in GaN electrodes greatly improves the electrical conductivity and mechanical stability, and takes the benefits of each element by integrating with the others through the synergies in their structure. The resultant nanocomposites have high capacitance, a fast charge–discharge rate, and an excellent cycling stability, making them prime national supercapacitor electrodes of the next generation. These developments not only serve to widen the possibilities that GaN‐based energy storage systems have but also serve to show that carbon‐based nanocomposites have larger applications in future high‐performance electrochemical systems.

### 10.2 Electrochemical Performance of GaN and Composites with Other Nitride‐Based Semiconductors

The electrochemical characteristics of nitrides in Table 8 show the structural and compositional versatility of the nitrides and the consequent change in capacitance, density of energy, and stability. Nitride materials like Co_3_N, Ni_3_N, VN, NbN, TiN, and GaN are known to possess unique charge‐storage properties, which are determined by their electron density, oxidation states, and electrolyte reactivity on the surface. Ni_3_N is the nitride with the greatest gravimetric capacitance (842 F g^−1^ at 10 mV s^−1^) and large energy output (120 Wh kg^−1^; 3390 W kg^−1^). This outstanding activity is attributed to its metallic conductivity and reversible Faradaic Ni^2+^/Ni^3+^ transitions. Nonetheless, it has low cycle retention (81 % at 250 cycles), which reveals its poor structural stability under repeated ion intercalation. Co_3_N also provides a high areal capacitance (449.2 mF cm^−2^) and retention of 92 % capacity after 10 000 cycles in alkaline media, which indicates that cobalt nitrides provide better kinetics sustained, but are susceptible to oxidation when in contact with atmospheric moisture.

**TABLE 8:** Electrochemical performance of GaN and other nitride‐based supercapacitors.

Electrodes	Electrolyte	Specific capacitance	Cycling stability	Energy density/Power density	Refs.
Co_3_N	6 M KOH	449.2 mF cm^−2^ (2 mA cm^−2^)	92.3% (10 000)	12.1 W h kg^−1^ and 204.6 W kg^−1^	[226]
VN	PVA/KOH	3.34 F cm^−2^ at 5 mA cm^−2^	96 % (10 000)	0.185 mWh cm^−2^ and 22.4 mW cm^−2^	[227]
TiN	1 M KCl	17 mF cm^−2^ (0.17 mA cm^−2^)	90 % (10 000)	23 mWh cm^−3^ and 7.4 mW cm^−3^	[228]
CrN	0.5 M H_2_SO_4_	31.3 mF cm^−2^ (1 mA cm^−2^)	94.0 % (20 000)	14.4 mWh cm^−3^ and 0.3 mW cm^−3^	[229]
MoN	1 M KOH	8.8 F cm^−2^ (5 mA cm^−2^)	100 % (10 000)	—	[230]
W_2_N	1 M H_2_SO_4_	163 F g^−1^ (0.5 mA cm^−2^)	90.46 % (10 000)	12.29 Wh kg^−1^ and 9.36 W kg^−1^	[231]
HfN	0.5 M H_2_SO_4_	5.6 m F^−2^ at 10 mV s^−1^	91.2 % (4000)	—	[232]
GaN crystals	EMImNTf_2_	52.58 mF cm^−2^ (0.8 mA cm^−2^)	86.2 % (10 000)	13.3 mWh cm^−2^ at 67.5 mW cm^−2^	[129]
NbN	0.5 M H_2_SO_4_	707.1 F cm^−3^ at 2 mV s^−1^	92.2 % (20 000)	—	[233]
Ni_3_N	6 M KOH	842 F g^−1^ at 10 mV s^−1^	81% (250)	120 W h kg^−1^ and 3390 W kg^−1^	[234]
Porous GaN	1 M H_2_SO_4_	21.22 mF cm^−2^ (0.1 mA cm^−2^)	99 % (10 000)	0.58 mWh cm^−2^ and 45 mW cm^−2^	[205]

Vanadium nitride (VN) exhibits high pseudocapacitive performance (3.34 F cm^−2^) and 96 % stability, which indicates its better conductivity and surface redox action. Nevertheless, the reliance on polymer gel electrolytes (PVA/KOH) restricts the voltage operation and enhances resistance to diffusion. Niobium nitride (NbN) has a very high volumetric capacitance (707.1 F cm^−3^) and retention of 92 % in 20 000 cycles is made possible by its dense crystal structure as well as chemical inertness with acidic electrolytes. These findings make NbN one of the most volumetrically efficient nitrides currently reported. Titanium nitride (TiN) has moderate areal capacitance (17 mF cm^−2^), high‐rate capability in neutral electrolytes (1 m KCl), but poor energy density (23 mWh cm^−3^) due to its highly electrostatic charge storage mechanism and low active surface. Chromium nitride (CrN) and Hafnium nitride (HfN) have low capacitance and yet highly stable electrodes, with more than 90 % remaining in use after 20 000 cycles, implying that passivation on the surface inhibits charge exchange but provides long‐term stability.

In the same dataset, the gallium nitrate (GaN) and its porous analogue can be found in a separate niche. In combination with ionic liquids, Transition‐metal nitrides using EMImNTf_2_ show 52.58 mF cm^−2^ at 0.8 mA cm^−2^ and 13.3 mWh cm^−2^ energy density, which is higher than much of the areal energy output of transition‐metal nitrides. The broad bandgap and thermal stability of GaN allow it to be used at longer potential windows, although the low carrier density and active surface indicate a limited capacitance. Porous GaN, however, has excellent retention of cycling (99 % in 10 000 cycles) as a result of its increased surface area and defect‐assisted charge storage, although at reduced areal capacitance (21.22 mF cm^−2^).

Focusing on key properties of nitride‐based electrodes from Table 8, it is clear that there is a stability‐capacitance trade‐off inherent with nitride electrodes: semiconducting nitrides, like GaN, are stable in aggressive electrolytes, but metallic nitrides, e.g., Ni_3_N and VN, offer high capacitance but degrade structurally. The high electrolyte dependence is observed–acidic and ionic‐liquid stabilize GaN surfaces, whereas the alkaline systems favor enhanced redox activity of transition‐metal nitrides but increase dissolution. The difference in behavior between these two discloses that the strength of GaN is not in the raw capacitance but in the electrochemical resilience and flexibility that make it a strategic platform for designing a hybrid supercapacitor.

Table 9 summarizes the electrochemical properties of composite and hybrid materials, which underscore the transformative effect of structural coupling between nitrides and conductive or redox‐active matrices. Composites based on GaN are the ones that exhibit significant performance improvement. GaN/MnO_2_/MnON tertiary hybrid attains 1915.5 mF cm^−2^ at 0.1 mA cm^−2^, with 95.5 % stability and 0.76 mWh cm^−3^ energy density. This is due to synergistic charge storage of the Mn redox couple (Mn^3+^/Mn^4+^) and the conductive backbone of GaN. However, the demand to use two electrolytes (Na_2_SO_4_ + KOH) makes it complex and can lead to interface degradation during long cycles. Equally, GaN/Ga_2_O_3_ heterostructures achieve very high capacitance (1301 mF cm^−2^) but intermediate retention (77 % after 20 000 cycles), probably because of strain at the interfaces and dissolution of oxides. These results show that high levels of redox activity, though raising initial capacitance, reduce mechanical and chemical stability.

**TABLE 9:** Electrochemical performance of GaN composites and other nitride‐based composites supercapacitors.

Electrodes	Electrolyte	Capacitance	Cycling stability	Energy density/Power density	Refs.
VN/CNT	0.5 M Na_2_SO_4_	178 mF cm^−2^ (1.1 mA cm^−2^)	82 % (10 000)	0.54 mWh cm^−3^ and 0.4 mW cm^−3^	[235]
Mo_2_N/rGO	SiWA‐H_3_PO_4_‐PVA	15.4 F cm^−3^ at 0.1 A cm^−3^	85.7 % (4000)	1.05 mWh cm^−3^ and 0.035 W cm^−3^	[236]
Nb_4_N_5_/rGO	PVA/H_2_SO_4_ gel	19 F cm^−3^ (at 0.1 A cm^−3^)	89 % (4000)	0.98 mW h cm^−3^ and 0.029 W cm^−3^	[237]
TiN@carbon	1 M KOH and 1 M Na_2_SO_4_	167 F g^−1^ at 1 A g^−1^	70 % (6000)	—	[238]
TiN*_x_* O*_y_*/MnO_2_	1 M H_2_SO_4_	95.5 mF cm^−2^ at 0.5 mA cm^−2^	93.88 % (10 000)	1.24 µWh cm^−2^ and 9.14 mW cm^−2^	[239]
MoN*_x_*/TiN	1 M LiOH	121.5 mF cm^−2^ (0.3 mA cm^−2^)	93.58 % (1000)	—	[240]
Co(OH)_2_//VN	2 M KOH	88.3 F g^−1^ at 0.2 A g^−1^	86 % (4000)	22 Wh kg^−1^ and 0.16 kW kg^−1^	[241]
Nb_4_N_5_/NC	1 M H_2_SO_4_ and 1 M Na_2_SO_4_	116.45 mF cm^−2^ (0.5 mA cm^−2^)	100 % (2000)	4.66 mWh cm^−3^ and 24.56 W cm^−3^	[242]
C/Ni_3_N	1 M Li_2_SO_4_	324 mF cm^−1^ at 0.5 mA cm^−2^	88.3 % (20 000)	15.26 µWh cm^−2^ and 811 µW cm^−2^	[243]
GaN/GP	1 M H_2_SO_4_	237 mF cm^−2^ (0.1 mA cm^−2^)	98 % (10 000)	0.30 mWh cm^−3^ and 1000 mW cm^−3^	[204]
TiN@MnO_2_	0.5 M Na_2_SO_4_	386 F g^−1^ at 10 mV s^−1^	111.7 % (4000)	—	[244]
GaN/MnO_2_/MnON	1 M Na_2_SO_4_ and 6 M KOH	1915.5 mF cm^−2^ (0.1 mA cm^−2^)	95.5 % (10 000)	0.76 mW h cm^−3^ and 125 mW cm^−3^	[213]
GaN/Ga_2_O_3_	1 M Na_2_SO_4_	1301.20 mF cm^−2^ (0.5 mA cm^−2^)	77.27 % (20 000)	0.58 Wh kg^−1^ and 154 W kg^−1^	[214]

A good example of a balanced hybrid design is the graphene paper with GaN, which attains 237 mF cm^−2^ capacitance, 98 % retention, and power density ∼1000 mW cm^−3^ in 1 M H_2_SO_4_, and good interfacial compatibility between the highly conductive carbon and the GaN. It is a stable and high‐speed ionic architecture with wide‐band semiconductor stability rates and is considered to prove the functionality of flexible graphene scaffolds in overcoming the inherent resistivity of GaN. Similar trends are observed in the case of non‐GaN composites. VN/CNT and Nb_4_N_5_/rGO systems combine metallic nitrides with carbon nanotubes or reduced graphene oxide systems, offering areal capacitances of 178 mF cm^−2^ and volumetric capacitances of 19 F cm^−3^, respectively. While retaining a stability of > 80 % over thousands of cycles. Mo_2_N/rGO has a balanced volumetric energy (1.05 mWh cm^−3^) and power density (0.035 W cm^−3^) that proves conductive carbon matrices to be efficient in dispersing nitride nanoparticles to suppress aggregation and retain the continuity of electrons through rapid charge discharge.

Core‐shell systems like TiN@MnO_2_ and TiN*_x_* O*_y_*/MnO_2_ also underscore the advantage of this architecture. In this case, TiN offers a conductive skeleton, and MnO_2_ offers surface redox reactions, resulting in capacitances up to 95 mF cm^−2^ and stability of over 93 %. Conversely, C/Ni_3_N and Co(OH)_2_//VN asymmetric cells provide large energy densities (15.26 µWh cm^−2^ and 22 Wh kg^−1^, respectively) but exhibit partial capacity decay as a result of volume expansion of the active material. Importantly, this table shows that all carbon hybridization is beneficial, with graphene, CNT, and N‐doped carbons enhancing the conductivity and accessibility to electrolytes and structural strain buffering, resulting in 10–20 % higher retention than the non‐hybrid versions. Moreover, electrolyte optimization will continue to be the center of attention: acidic and neutral electrolytes stabilize the GaN interfaces, and alkaline environments maximize pseudocapacitive reactions in VN and Ni_3_N composites.

The data about the electrochemical properties of these composites establishes that, through hybridization, GaN is converted into a multifunctional energy‐storage scaffold that can compete with high‐performance transition‐metal nitrides in terms of capacitance and maintain excellent stability. It is the interaction between surface chemistry, interfacial bonding, and electrolyte environment that is thus critical: excess Faradaic contribution is ideal on capacity, but poor on durability, whereas balanced heterointerfaces (e.g., GaN/GP) achieve both power and longevity. Such results make GaN‐based heterostructures a bridge material: between the chemical stability of wide‐band semiconductors and the high charge‐storage kinetics of metallic nitrides, thereby providing a general platform for next‐generation supercapacitors.

## 11 Device‐Level Integration and Architectures

Introduction of gallium nitride (GaN) into the levels of architectures in devices enables a filler of its inherent semiconductor characteristics to effective energy storage mechanisms. Due to its broad bandgap, large electron mobility, and outstanding chemical stability, GaN provides a solid foundation to supercapacitor technologies, but it should be limited by its low conductivity, which can be offset by the formation of composites. Thin film, nanostructured layers of GaN can easily be incorporated in flexible, solid‐state, and optoelectronic energy‐storage devices because of easy deposition [245, 246]. GaN has the potential to form mechanically deformable devices that are electrically stable in flexible micro‐supercapacitors (MSCs) due to the mechanical resilience of GaN and its ability to be used together with carbon‐based scaffolds [247, 248]. In solid‐state designs, GaN thermal and chemical stability allows solid‐electrolyte integration, reducing leakage and enhancing reliability with portable or miniaturized designs. The characteristics also support its future multifunctional photonic supercapacitors, such that the optoelectronic response of GaN can be used in light‐assisted charge generation and high capacitance [249, 250].

GaN thin films also expand to an on‐chip and transparent system of energy storage. Their optical transparency in the visible region and their interchangeability with semiconductor manufacturing processes have rendered them very suitable for integration with microelectromechanical systems (MEMS) [251, 252], display smart displays and transparent endeavors. Surface area and carrier pathways can be finetuned by controlling local film thickness and morphology, which is critical to high areal capacitance and rapid charge transport. The rGO‐GaN composites are small crystallites at the nanoscale (0.243 – 0.245 nm) that demonstrate how atomic‐scale accuracy can maximize the electrochemical efficiency of a thin‐film [253]. The possible uses of GaN‐based electrodes can be found in the experimental areas of wearables, autonomous sensor systems, and hybrid optoelectronic energy systems.

GaN‐carbon and polymer‐based flexible composites are especially well‐positioned to support wearable electronics with their capability to absorb mechanical stress on the material, and with this metal being semiconducting in nature, it can be co‐engineered into a self‐powered sensing system, with energy harvesting and gas sensing modules being capable of coexisting on the same architecture [177]. The powerful UV absorption of GaN in optoelectronic hybrids allows charge to be loaded sporadically by photo, which allows routes to self‐charging or solar‐coupled supercapacitors. Synergistic material combinations bring about the functional improvement of such integrated systems. Conductive carbons contain high interfacial areas and fast electron routes [254], whereas the conducting polymers like PANI and PPy contain reversible reductive oxidative reactions that increase specific capacitance and energy density [255]. Hybrid designs are representative of the scaling design principles that should be used in next‐generation GaN‐based flexible and solid‐state supercapacitors.

### 11.1 Challenges and Limitations

Regardless of its potential attributes, there are a number of inherent and external challenges to the practical implementation of gallium nitride (GaN)‐based materials in supercapacitors. The production of GaN and its nanocomposites is still complicated and energy‐intensive, with the majority of the growth processes being based on high‐temperature vacuum growth techniques, including metal‐organic chemical vapor deposition (MOCVD) and molecular beam epitaxy (MBE) [256, 257]. These methods need expensive raw materials, closed incubators, and sophisticated apparatuses, which render mass production a challenge. Even though the chemical reduction process has allowed the production of reduced graphene oxide/gallium nitrate nanocomposites with better electrochemical properties, ensuring stoichiometric accuracy, interfacial uniformity, and scale‐up is a significant challenge.

The very low surface area of bulk GaN also contributes to the limitation of charge storage ability since the electrode‐electrolyte interface is also limited. Although nanoscale structuring and thin‐film deposition raise the available surface area, the values nevertheless remain lower than those provided by carbon‐based materials. In turn, GaN is frequently used together with high‐surface‐area materials: rGO, conducting polymers, or metal oxides because it has limited porosity and increased electrochemical reactivity. Industrial translation is limited by the unavailability of standardized, scalable fabrication methods of these hybrid architectures: most lab‐level syntheses have been multi‐step, with a critical component of phase placement, and cannot be readily reproduced in mass‐scale manufacturing.

Another severe constraint of the GaN‐based supercapacitors is interface instability and lattice mismatch. The strain caused by the mismatch between the GaN films and the metallic current collectors tends to bring about dislocations, adhesion faults, and resistance at the interface, which impairs the efficiency of charge‐transfer at longer cycle periods [167]. More so, defect distributions along interfaces can be changed by ion implantation processes (and, most notably, Si or Mg dopants, which change the defect distribution in band bending and electronic coupling. Long‐term stability in electrochemical behavior and mechanical stability of the electrochemical and mechanical properties of the GaN/conductor and GaN/composite junctions is therefore necessary by ensuring the structural coherence and chemical compatibility of the interface.

The strategies of future improvement include electronic‐structure engineering and data‐based optimization of materials. Doping or alloying of bandgaps in GaN can offer a useful strategy to increase the conductivity and photoelectrochemical responsiveness of the material. DFT simulations of controlled introduction of dopants like fluorine, germanium, or carbon have been demonstrated to induce new states in the projected density of states (PDOS), shifting the levels of energy at and around the Fermi energy and facilitating more efficient charge transfer [164]. Diffusion and charge transport are also affected by these lattice constants and defect formation energies (indirectly) as a result of these electronic changes.

Other avenues of performance improvement include defect passivation and optimization of the heterostructure. Plasma activation, etching, and oxidation are all surface treatments that can deactivate trap states related to vacancies of nitrogen and gallium and enhance carrier mobility and inhibit recombination. The combination of artificial intelligence (AI) and computational models is becoming one of the groundbreaking methods to speed up the process of searching for materials based on GaN. DFT‐derived datasets can be trained with machine learning algorithms to be able to predict dopant energetics, surface adsorption phenomena, and interface stability with high accuracy, and thus a wide range of novel material compositions and synthesis conditions can be screened quickly.

### 11.2 Challenges at Industrial Level

The process of transferring gallium nitride (GaN)‐based supercapacitors from laboratory research to industrial deployment is hindered by a number of bottlenecks. GaN is reported to possess desirable intrinsic properties, including high power density, thermal stability, and fast charge–discharge rates; however, limitations in fabrication scalability, high material costs, and complex processing requirements restrict its practical industrialization [7, 258].

Engineered nanostructures, such as porous membranes, nanowires, and hierarchically structured crystal architectures, are often employed in the fabrication of high‐performance GaN electrodes. Although these nanostructures are effective in enhancing electrochemical performance, they are typically difficult to reproduce consistently at the wafer scale. As a result, a significant disparity exists between small‐area experimental demonstrations and the large‐area, high‐yield production required for industrial implementation [26, 204].

#### 11.2.1 Large‐Area Fabrication and Scalability

Achieving consistent structural control over large substrate areas represents one of the most critical challenges to industrialization. Studies on porous GaN fabrication indicate that variations in etching and processing parameters, including applied bias, electrolyte composition, and illumination conditions, can lead to heterogeneous pore morphology, mechanical fragility, and non‐uniform electrochemical performance. These factors severely limit reproducibility and scalability when transitioning from laboratory‐scale samples to wafer‐level manufacturing [160].

In addition to morphological variability, substrate‐related challenges further complicate large‐area fabrication. GaN is commonly grown epitaxially on non‐native substrates such as sapphire or silicon, resulting in lattice and thermal mismatch stresses [259, 260]. These mismatches generate high defect densities and residual strain within the GaN layers, which can lead to wafer bowing, film cracking, and reduced device yield during large‐scale production [261, 262].

From a synthesis perspective, established growth techniques such as metal‐organic chemical vapor deposition (MOCVD) are widely used to produce high‐quality GaN thin films. However, these methods operate at high temperatures and under tightly controlled conditions, increasing process complexity and limiting throughput. Alternative approaches aimed at improving scalability, such as electrochemical solution growth (ESG), enable lower‐temperature and atmospheric‐pressure growth. Despite these advantages, achieving consistently high electronic and structural quality remains challenging [263, 264].

#### 11.2.2 Cost Reduction and Market Viability

Another major barrier to industrial deployment is the high cost of GaN‐based materials, which is frequently cited as a reason for the continued dominance of silicon‐based technologies in the semiconductor industry [265]. In supercapacitor applications, porous GaN electrodes are considerably more expensive to fabricate than conventional carbon‐based electrode materials, such as activated carbon, graphene, or carbon nanotubes, which benefit from well‐established and low‐cost manufacturing routes. Economic viability is closely linked to economies of scale. Industry analysts suggest that substantial cost reductions can only be achieved through the transition from smaller wafer formats to large‐scale manufacturing on 8‐inch and, eventually, 12‐inch wafers [266]. Such scaling has the potential to significantly reduce the cost gap between GaN and conventional semiconductor materials [263]. In addition, retrofitting existing silicon fabrication facilities for GaN processing has been proposed as a cost‐effective alternative to constructing new production plants, thereby lowering capital investment requirements.

#### 11.2.3 Processing and Supply Chain Constraints

Beyond fabrication and cost considerations, GaN‐based supercapacitors face additional processing and integration challenges. The development of reliable etching protocols and low‐resistance Ohmic contacts is essential for stable device operation. Manufacturing‐oriented studies indicate that these processes often require customized optimization, which limits compatibility with existing CMOS manufacturing infrastructures and increases overall production complexity [267].

Industrial readiness is also influenced by supply chain and workforce limitations. The global GaN supply chain is vulnerable to disruptions and geopolitical risks, particularly those associated with gallium availability. Furthermore, the limited availability of skilled personnel and the absence of standardized design and qualification protocols can slow the pace of industrial adoption [268, 269].

#### 11.2.4 Environmental and Sustainability Considerations

As GaN‐based energy storage technologies approach commercialization, increasing attention is being directed toward their environmental impact. Reviews of gallium‐based electrode materials emphasize the need to develop environmentally friendly synthesis routes and efficient recycling strategies to minimize environmental burden and improve long‐term sustainability. Addressing these challenges will be critical for the responsible large‐scale deployment of GaN‐based supercapacitors [270]. The summary of challenges to industrialization including fabrication, cost, integration and market for GaN‐based supercapacitors is given in Table 10.

**TABLE 10:** Summary of challenges to industrialization of GaN‐based Supercapacitors.

Challenge category	Specific bottlenecks	Potential solutions / Trends
**Fabrication**	Non‐uniform pore distribution; lattice‐mismatch‐induced stress; complex and process‐sensitive etching protocols.	Photo‐electrochemical (PEC) etching frameworks; transition to larger wafer sizes (8–12 inches) to improve uniformity and yield.
**Cost**	High substrate costs and reliance on expensive metal‐organic precursors, contributing to poor cost competitiveness.	Electrochemical solution growth (ESG) methods; retrofitting existing silicon fabrication facilities; cost reduction through economies of scale.
**Integration**	Difficulties in CMOS integration; variability in Ohmic contact resistance and process repeatability.	Advanced packaging strategies; room‐temperature bonding using conductive materials; improved process standardization.
**Market**	Geopolitical supply risks; lack of long‐term reliability and qualification data under real operating conditions.	Multi‐fab sourcing strategies; extensive field testing; hybrid integration with Li‐ion systems to accelerate market adoption.

## 12 Future Insights

**FIGURE 21:**
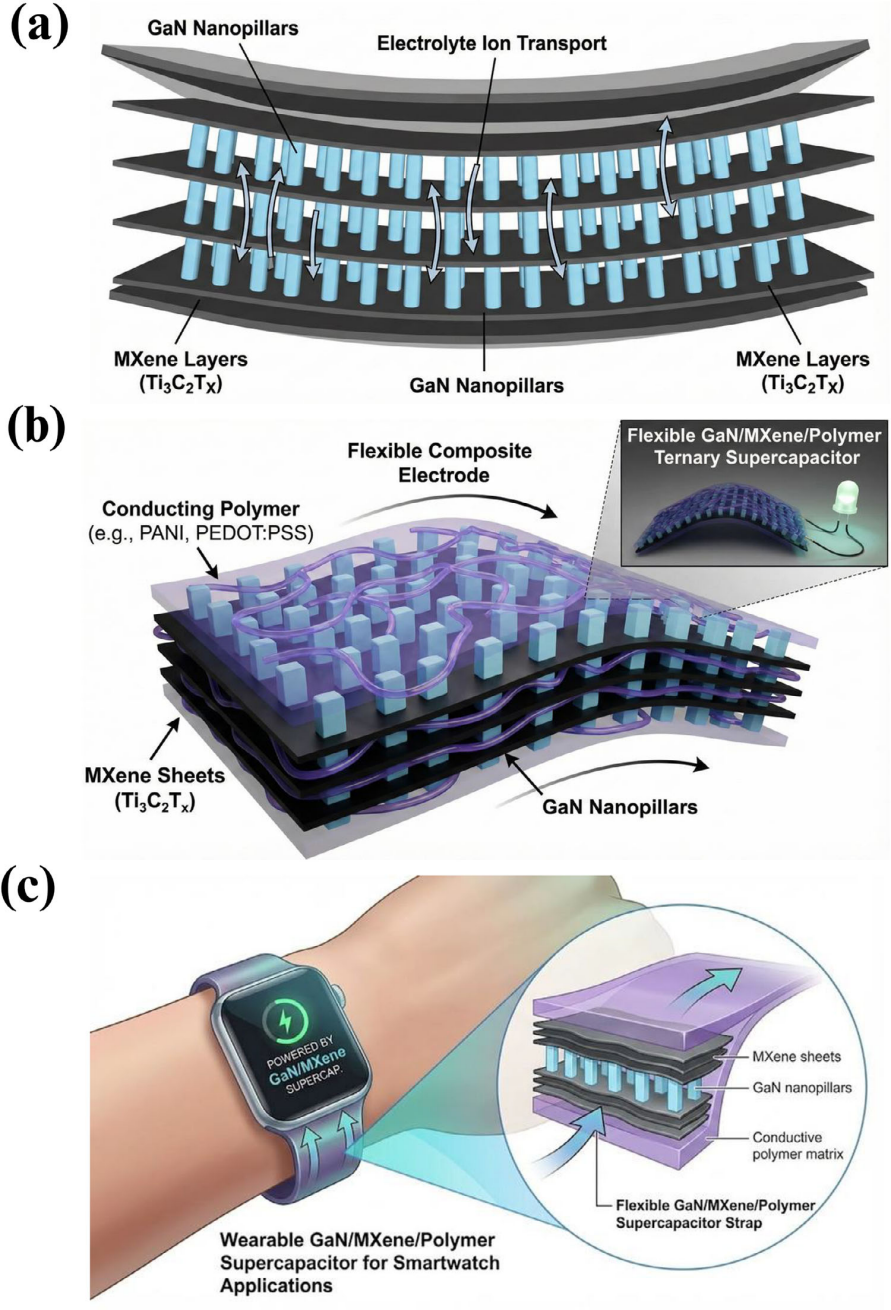
(a) An Illustration of GaN Nanopillars between MXenes Sheets, (b) Schematic of GaN/MXenes/Polymers for flexible device applications, (c) An illustration of a GaN/MXenes/Polymer‐based supercapacitor flexible watch for wearable technology.

GaN‐based supercapacitors have become the leaders of a new energy storage revolution and are being developed at an accelerated pace as the research gains more focus. The development of the field is rapid, as the increasing need for more quickly charging devices with high energy densities and high durability promotes its development. The need to develop further enhanced and better property‐based options for areas of application like electric vehicles (EVs), and grid grid‐scale energy storage, and portable electronics, because traditional technology is lacking in performance metrics. Researchers are trying to use extensive features of gallium nitride (GaN), such as its high thermal stability at 600°C and large bandgap, with the possibility to operate at high voltages, so that products with better performance metrics could be developed. However, the challenge of moderate inherent electrical conductivity of GaN reduces its energy storage capabilities. To overcome this, researchers are coming up with composite materials by incorporating GaN with the use of MXenes [271, 272, 273], a high electrical conductor and tunable surface chemistries, or conductive polymers that offer mechanical flexibility and other charge storage locations. When combining with MXenes, GaN will form nanopillars between the layers of MXenes to overcome the restacking issue, which can be further used for flexible applications like watches. Moreover, inclusion of some polymer like PANI, PPy, PEDOT, etc can add up to more flexibility of the device as shown by Figure 21a,b, while Figure 21c is an illustration of a GaN‐based supercapacitor for future flexible wearable technology, such as Smart watches and bands.

These developments are not a mere improvement process, but they are rather a paradigm shift in material design. Preliminary research suggests that it is possible to achieve energy densities of over 50 Wh kg^−1^ and power densities of over 10 kW kg^−1^ directly to meet the fast‐charging needs of EVs' next generation and the high‐power needs of new electronic systems.

Besides composites, strategic doping of GaN with other elements, e.g., silicon or magnesium, is becoming an efficient approach to managing the concentrations of charge carriers and to fine‐tune the electrochemical performance. When the dopants of high surface area (over 500 m^2^ g^−1^) are incorporated, there is a challenge to retain the crystalline lattice, as this would lead to a deterioration in performance. And at the nanoscale, the management of such properties is essential because the effects of small structural defects on conductivity and stability are non‐linearly disproportionate. To achieve a reproducible, uniform, and controlled manner of creating dopants, researchers are developing precision methods of synthesis. Another useful material that is relevant here is graphene with its outstanding electrical and mechanical properties. When mixed with GaN, it makes composite anodes that have high operating voltages, high cycling stability, faster charge–discharge kinetics, and high environmental resistance qualities, which are highly desirable to supercapacitors and the general battery technology sector. This reciprocity is paving the way to new hybrid devices that can fill the gap between batteries and supercapacitors, which may allow energy storage systems that would have the most beneficial features of both systems.

The conversion of these material developments into real working devices presents great engineering problems. GaN‐on‐polymer substrates are proving to have the highest potential in the area of flexible electronics, especially in future wearable systems and flexible sensors [101]. Current prototypes are still amazingly resilient, with more than 95 % of their capacitance remaining after 5000 bending cycles at radii as small as 2 mm, demonstrating their mechanical strength and ability to be integrated into textiles or curved surfaces. Monolithic GaN power modules are also a promising solution to large‐scale energy storage, as in the case of grid‐level buffering of renewable energy. The modules can be used as they have good operation at high and low temperatures −50°C to 300°C, hence these modules are good in any harsh environment where temperature stability is essential.

Another potential frontier is that of self‐charging systems. Researchers are thinking about integrated energy solutions to the Internet of Things (IoT) by combining GaN supercapacitors with perovskite solar cells—famous due to their very high‐power conversion efficiencies—or by using triboelectric nanogenerators that generate energy by tapping into ambient mechanical power. Such systems might be unattended, putting remote sensors and devices in areas that are not reachable without the need to replace batteries or maintain them, thus saving a lot of money and harming the environment. Although these developments have been made, some important obstacles still lie before GaN‐based supercapacitors can be brought to large‐scale commercial applications. The lack of uniform ways of assessing the long‐term aging and reliability of the GaN‐electrolyte systems is among the most important issues. The industry desperately needs faster aging tests that would be able to mimic years or decades of operational use in weeks or months, so that manufacturers and end‐users can comfortably determine the long life of the device and its reliability.

A significant roadblock is the process of scaling demonstrations done in laboratories to large‐scale manufacturing facilities. Intensive research is currently underway to create large‐scale fabrication of GaN films by processes like plasma‐enhanced atomic layer deposition (PEALD) [274], which, though still expensive, is anticipated to lower the cost of production by up to 50 % of that of the currently predominant metal‐organic chemical vapor deposition (MOCVD) and hold material quality and throughput. In order to commercialize it successfully, uniformity in large substrates and accurate control of defects will be required.

New advanced characterization techniques, such as in situ transmission electron microscopy (TEM) and density functional theory (DFT) calculations, are highly featured on the basic research level. Because the atom‐level dynamics with such a technique at the interfaces between the electrolyte and GaN can be recorded under operating conditions, especially at large currents of over 5 V, the processes at interfaces can be of much importance to the overall functionality of the device. The result of this study will be what defines the future of the emerging generation materials that have the best properties of charge storage and charging discharging. Thus, the fabrication of new substances, the accuracy of device manufacturing, the volume of production, and the overall knowledge of the principles are leading the way to the emergence of GaN‐based supercapacitors to compete and even exceed the traditional possibilities of energy storage. As these challenges are slowly solved, a generation of supercapacitors would serve as the most important elements to enable electric cars, renewable energy, wearable electronics, and self‐sufficient Internet of Things (IoT) devices, which would re‐establish the boundaries of the present energy storage potential.

## 13 Conclusion

GaN‐based materials are already the most promising materials for the next generation of supercapacitor electrodes. They possess a considerable advantage in the form of a large bandgap, high mobilities of electrons, heat, and chemical resistance. Scientists have gone to the extreme of developing porous structures, nanocomposites like GaN/MnO_2_/MnON, GaN/Ga_2_O_3_, and GaN/NiCoO_2_, structures, and heterostructures. These engineered materials are synergistic, and they work together to promote electrochemical performance based on high specific capacitance, long life cycles, and high energy and power densities. They store charge rapidly, transport rapidly, and they are structurally stable even though they may have numerous applications or extreme conditions. Of course, one has to overcome several difficulties as well: one has to raise the accessible surface area, optimize the electrical conductivity, and acquire these materials in large quantities at a low cost. Nevertheless, the studies to date result in it being obvious, GaN is multifunctional and strong. GaN‐based electrodes are also poised to dominate more sophisticated supercapacitor technology as new concepts of material designs come into being and manufacturing at a scale becomes accessible. They are prepared to meet the demanding nature of future energy storage, and things in the field are not about to decelerate.

## Author Contributions

**Farasat Haider**: writing – original draft, visualization, conceptualization, project administration, **Batool Esmat**: writing – original draft, validation, **Ali Raza Kashif**: investigation, conceptualization, **Muhammad Shahid Khan**: validation, Resources, **Akif Safeen**: writing – review & editing, Formal Analysis, **Basit Ali Khan**: validation, visualization, **Karim Khan**: writing – review & editing, Formal Analysis, and **Basit Ali**: supervision, funding acquisition, investigation, project administration.

## Conflicts of Interest

The authors confirm that there are no financial or personal conflicts that could have affected the objectivity or integrity of the research presented in this manuscript.

## Data Availability

The data that support the findings of this study are available from the corresponding author upon reasonable request.
